# Genome-wide identification and expression profiling analysis of maize AP2/ERF superfamily genes reveal essential roles in abiotic stress tolerance

**DOI:** 10.1186/s12864-022-08345-7

**Published:** 2022-02-12

**Authors:** Jing Zhang, Jiayao Liao, Qiqi Ling, Yan Xi, Yexiong Qian

**Affiliations:** Anhui Provincial Key Lab. of the Conservation and Exploitation of Biological Resources, Anhui Normal University, Wuhu, 241000 China

**Keywords:** AP2/ERF transcription factor, Expression patterns, Gene duplication, Abiotic stress, Regulatory network, *Zea mays L*

## Abstract

**Background:**

As one of the largest transcription factor families in plants, the APETALA2/Ethylene-Responsive Factor (AP2/ERF) superfamily is involved in various biological processes and plays significant roles in plant growth, development and responses to various stresses. Although identification and characterization of AP2/ERF superfamily genes have been accomplished in many plant species, very little is known regarding the structure and function of *AP2/ERF* genes in maize.

**Results:**

In this study, a total of 214 genes encoding ZmAP2/ERF proteins with complete AP2/ERF domain were eventually identified according to the AGPv4 version of the maize B73 genome. Based on the number of AP2/ERF domain and similarities of amino acid sequences among AP2/ERF proteins from *Arabidopsis*, rice and maize, all 214 putative ZmAP2/ERF proteins were categorized into three distinct families, including the AP2 family (44), the ERF family (166) and the RAV family (4), respectively. Among them, the ERF family was further subdivided into two diverse subfamilies, including the DREB and ERF subfamilies with 61 and 105 members, respectively. Further, based on phylogenetic analysis, the members of DREB and ERF subfamilies were subdivided into four (Group I-IV) and eight (Group V-XII) groups, respectively. The characteristics of exon-intron structure of these putative *ZmAP2/ERF* genes and conserved protein motifs of their encoded ZmAP2/ERF proteins were also presented respectively, which was in accordance with the results of group classification. Promoter analysis suggested that *ZmAP2/ERF* genes shared many stress- and hormone-related cis-regulatory elements. Gene duplication and synteny analysis revealed that tandem or segmental duplication and purifying selection might play significant roles in evolution and functional differentiation of AP2/ERF superfamily genes among three various gramineous species (maize, rice and sorghum). Using RNA-seq data, transcriptome analysis indicated that the majority of *ZmAP2/ERF* genes displayed differential expression patterns at different developmental stages of maize. In addition, the following analyses of co-expression network among *ZmAP2/ERF* genes and protein protein interaction between ZmAP2 and ZmERF proteins further enabled us to understand the regulatory relationship among members of the AP2/ERF superfamily in maize. Furthermore, by quantitative real-time PCR analysis, twenty-seven selected *ZmAP2/ERF* genes were further confirmed to respond to three different abiotic stresses, suggesting their potential roles in various abiotic stress responses. Collectively, these results revealed that these *ZmAP2/ERF* genes play essential roles in abiotic stress tolerance.

**Conclusions:**

Taken together, the present study will serve to present an important theoretical basis for further exploring the function and regulatory mechanism of *ZmAP2/ERF* genes in the growth, development, and adaptation to abiotic stresses in maize.

**Supplementary Information:**

The online version contains supplementary material available at 10.1186/s12864-022-08345-7.

## Background

Various biotic and abiotic stresses including high salinity, drought, extreme temperature, flood, submergence and pathogen attack, exert negative impacts on the growth and development of plants. Currently, abiotic stresses have been confirmed to be one of the main factors responsible for the loss of crops worldwide and can lead to the decline of yields of most major crop plants by up to 50% [1, 2]. To survive, grow and reproduce under abiotic stresses, plants have evolved a complex regulatory mechanism through activation or suppression of a range of defense-responsive genes with multiple diverse functions. Transcription factors (TFs), which can specifically bind to cis-acting elements in promoters of specific target genes to regulate gene expression [3], play critical roles in related regulatory networks or signaling pathways responding to abiotic stresses [4].

In plants, the APETALA2/Ethylene-Responsive Factor (AP2/ERF) superfamily is one of the largest families of plant TFs [5] and the AP2/ERF superfamily proteins contain at least one highly conserved AP2/ERF DNA-binding domain comprising 50–60 amino acid residues. It can be divided into three distinct families according to the number and similarity of AP2/ERF DNA-binding domain, namely the AP2 family, the ERF family (including two subfamilies ERF and DREB) and the RAV family [6, 7]. The AP2 family proteins possess two repeated AP2 domains, the ERF family proteins only share a single AP2 domain and a conserved WLG-motif, while the RAV family proteins contain an AP2 domain as well as a B3-like domain, a plant-specific DNA-binding domain that is conserved within other transcription factors [8]. Additionally, members with AP2-like domain, but lacking the extra motif were regularly identified as soloist [9].

In plants, the AP2/ERF TFs are the critical factors involved in regulating plant growth and development and responding to environmental stimuli including biotic and abiotic stresses. The members of AP2 family have been illustrated to play a major role in regulating the development of vegetative and reproductive organs [10], including the floral homeotic proteins APETALA2(AP2) [11] and AINTEGUMENTA [12, 13], which can control cell number and growth to be involved in the lateral organ development [14, 15]. Previous studies have demonstrated that the RAV family genes play an important role in responses to ethylene, brassinosteroid and abiotic stresses [8, 16, 17]. The ERF family can be further divided into two subfamilies: the ERF and DREB subfamilies [9, 18]. The ERF subfamily proteins can bind to a conserved AGCCGCC sequence known as ethylene-responsive (ERE) element, which not only participates in regulating gene expression in plant abiotic stress responses, but is also closely associated with the regulation of plant resistance genes [19], while the DREB subfamily proteins interact with the cis-acting DRE/C-repeat (DRE/CRT) element with a core motif of A/GCCGAC in promoters of stress-induced genes to regulate gene expression and also respond to plant hormones such as ethylene, ABA and so on [20, 21]. Furthermore, diverse functions of some AP2/ERF superfamily genes particularly involved in plant abiotic stress responses have been revealed. In previous studies, it has been demonstrated that two transcription factor genes *AtDREB2A* and *AtDREB2B* can be induced by heat stress, which is of great significance for the study of plant heat resistance mechanism. Overexpression of the *AtDREB2A* gene can transcriptionally induce heat stress-related transcription factor gene *AtHsfA3*, and thereby regulate the expression levels of some heat stress-induced genes to improve heat stress tolerance in *Arabidopsis* [22]. What’s more, overexpression of the *ZmDREB2A* gene in maize has been also revealed to enhance the heat stress tolerance [23]. In *M. truncatula,* the transcription factor gene *MtCBF4* has been verified to participate in response to abiotic stresses including cold, drought, salt, and ABA [24]. Recently, *CaAIEF1* has been illuminated to have a positive response to ABA signaling and drought stress in pepper [25]. Therefore, increasing studies have suggested that the AP2/ERF subfamily genes are widely involved in responses to abiotic stresses in plants.

Maize (*Zea mays L*.), as one of the most important model monocot species for functional genomics analysis, is very important to the study of molecular biology of plants. However, studies on the molecular mechanism of *ZmAP2/ERF* genes in maize responding to abiotic stresses remain severely limited. Genome-wide identification and characterization of maize AP2/ERF superfamily genes can contribute to better exploring molecular mechanisms of resistance to biotic or abiotic stresses. The high-quality sequencing of the maize genome has been completed, which give us an excellent chance to identify and dissect the AP2/ERF superfamily genes in maize. In this study, a comprehensive analysis of *ZmAP2/ERF* genes was conducted, including their phylogenetic relationship, gene structure, conserved motifs, cis-elements, chromosome distribution, gene duplication and evolutionary mechanism as well as expression profiles and possible regulatory network analysis in genome-wide level, which will lay a firm foundation for further exploring the function and regulatory mechanism of *ZmAP2/ERF* genes in the growth, development and adaptation to abiotic stresses in maize.

## Results

### Identification and analysis of AP2/ERF superfamily genes in maize

In this study, candidate genes of the AP2/ERF superfamily were firstly obtained through the maize genomic database. Then, all protein sequences of these putative *ZmAP2/ERF* genes were further confirmed by SMART search and NCBI-CDD web server for the existence of AP2/ERF domain. Finally, a total of 214 putative *ZmAP2/ERF* genes were identified to encode proteins containing complete AP2/ERF domain(s). Subsequently, according to the number of AP2/ERF domain and the similarity of amino acid sequences, all 214 putative ZmAP2/ERF proteins were categorized into three distinct families, including the AP2 family (44), the ERF family (166) and the RAV family (4). Among them, 36 genes were identified to encode proteins with two AP2/ERF domains in maize, whereas only 34 genes were classified into the AP2 family owing to the remaining two genes (*ZmERF8* and *ZmERF9*) encoding proteins containing two AP2 domains, which were more homologous to the ERF family and thus classified into the ERF family. Similarly, 174 genes were determined to encode proteins with a single AP2/ERF domain, whereas only 164 genes were divided into the ERF family. The remaining ten genes (*ZmAP2–2, ZmAP2–6, ZmAP2–14, ZmAP2–19, ZmAP2–25, ZmAP2–26, ZmAP2–27, ZmAP2–28, ZmAP2–40* and *ZmAP2–44*), also encoded an AP2/ERF domain, which was distinct from the ERF family and instead more associated with the AP2 family members. For this reason, they were clustered into the AP2 family. The total of 166 genes identified as the ERF family could be further subdivided into two subfamilies including the ERF and DREB subfamilies based on the similarity of amino acid sequences. Among them, 61 genes were predicted to encode the members of the DREB subfamily, while another 105 genes were assigned to the ERF subfamily. The RAV family only contained 4 putative genes, which were predicted to encode the proteins with one AP2/ERF domain as well as one B3 domain. These *ZmAP2/ERF* genes were uniformly named on the basis of classification of the AP2/ERF superfamily and chromosome locations of their corresponding genes (Table S1). Then, the online server PROTPARAM (http://web.expay.org/protparam/) was utilized to analyze the physicochemical properties of each putative ZmAP2/ERF protein. As shown in Table S1, their sequences were highly diverse with respect to the length (124-706AA) and molecular weight (13,231.6–73,216.7 kDa) of these identified proteins. What’s more, molecular weight of the AP2 family proteins was generally greater than that of the ERF or RAV family proteins, and gene length and coding region length of the AP2 family members were also generally longer than that for the ERF and RAV family members.

### Multiple sequence alignment and characterization of maize AP2/ERF proteins

To evaluate the phylogenetic relationship among the ZmAP2/ERF superfamily proteins, multiple sequence alignment analysis was performed on the basis of amino acid sequences of conserved AP2/ERF domains. The alignment among the ERF family members suggested that the proteins of the DREB subfamily were highly conserved in maize (Fig. S1A & B). All the maize DREB subfamily proteins contain WLG (27th to 29th amino acid residues) elements and most of these proteins also contain YRG (2nd to 4th amino acid residues) elements (Fig. S1B). These results showed that there were eighteen amino acid residues, namely Gly-4, Arg-6, Arg-8, Trp-13, Val-14, Glu-16, Arg-18, Pro-20, Arg-25, Trp-27, Leu-28, Gly-29, Ala-37, Ala-38, Ala-39, Alu-40, Asp-42 and Asn-56, which was completely conserved among 61 proteins from the DREB subfamily. Moreover, more than 95% of the DREB subfamily proteins contain conserved Val-5, Gly-11 and Leu-55 residues. All the DREB subfamily proteins also contain Valine in the Val-14 position, which may play a crucial role in the binding of DREB to the DRE/CRT cis-element [26] (Fig. S1B). In the ERF subfamily, the WLG (27th to 29th amino acid residues) element was also revealed. However, three transcription factors (ZmERF111, ZmERF112 and ZmERF14) contain WIG (27th to 29th amino acid residues), while one transcription factor ZmERF15 contains WVG (27th to 29th amino acid residues) (Fig. S1A). The alignment indicated that all the ERF subfamily proteins from maize contained the residues Gly-14, Trp-27, Gly-29, Ala-37and Try-41, and more than 95% members of them contained Arg-6, Arg-8, Gly-11, Glu-16, Ile-17, Arg-25, Leu-28, Thr-30, Ala-38, Asp-42, Gly-50, Asn-56, Phe-57 residues (Fig. S1A). The sequence alignment results among maize ERF family members were consistent with the previous study, which demonstrated that the Ala-37 on the α-helix and conserved Arg and Trp residues in the β-sheet might play a critical role in DNA binding of the AP2/ERF domain [27]. However, three proteins (ZmERF8, ZmERF9 and ZmERF145) were confirmed to show low homology with the consensus sequences of N-terminal regions of AP2/ERF domains that corresponds to the second β-sheet.

The multiple sequence alignment among the AP2 family members showed that thirty-four AP2 proteins with two AP2/ERF domains and another ten AP2 proteins (namely ZmAP2–2, ZmAP2–6, ZmAP2–14, ZmAP2–19, ZmAP2–25, ZmAP2–26, ZmAP2–27, ZmAP2–28, ZmAP2–40, ZmAP2–44) containing one AP2/ERF domain but lacking WLG elements (27th to 29th amino acid residues) were assigned to the AP2 family in maize (Fig. S1C). In addition, there were four other proteins, namely ZmRAV-1, ZmRAV-2, ZmRAV-3 and ZmRAV-4, which were detected to contain one single AP2/ERF domain as well as one B3 domain (Fig. S1D). Thus, these four proteins were clustered into the RAV family in maize.

### Phylogenetic analysis of maize AP2/ERF proteins

**Fig. 1:**
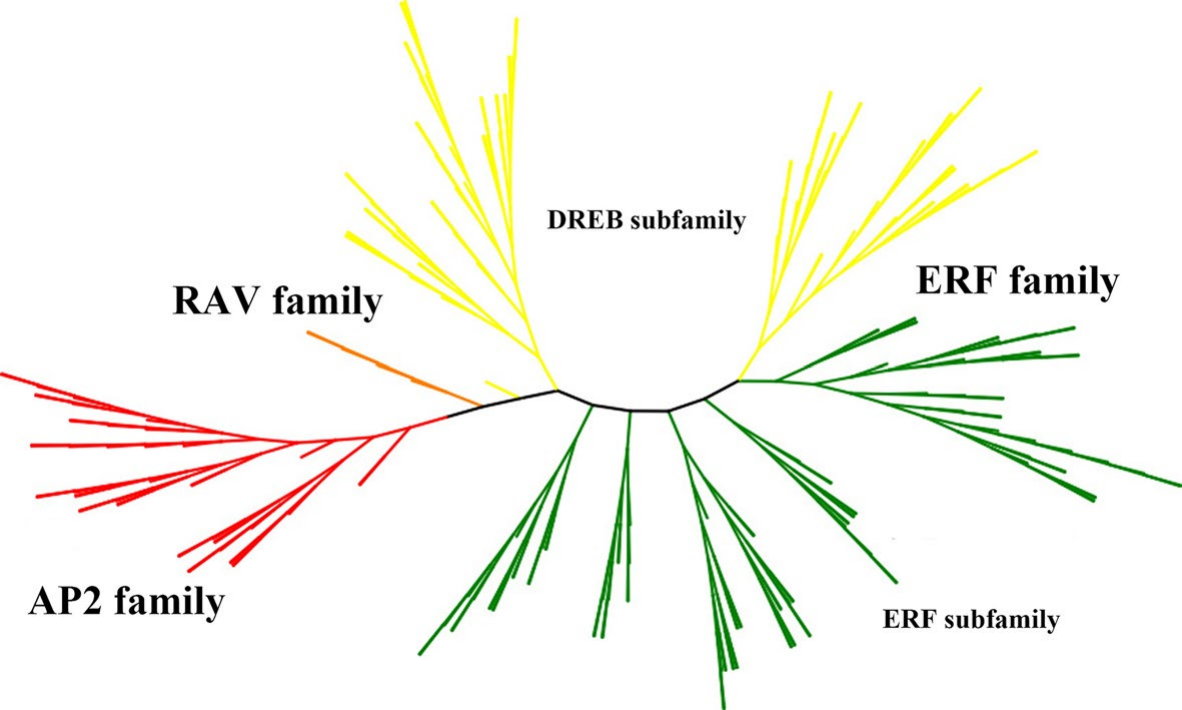
Phylogenetic analysis among AP2/ERF proteins of maize, rice and *Arabidopsis*. The phylogenetic tree was constructed using MEGA 7.0 and the numbers are bootstrap values based on 1000 iterations. The tree was divided into four clades, which contained ERF (DREB and ERF subfamily), AP2 and RAV family and the branch lines for each clade are colored consistently

**Fig. 2:**
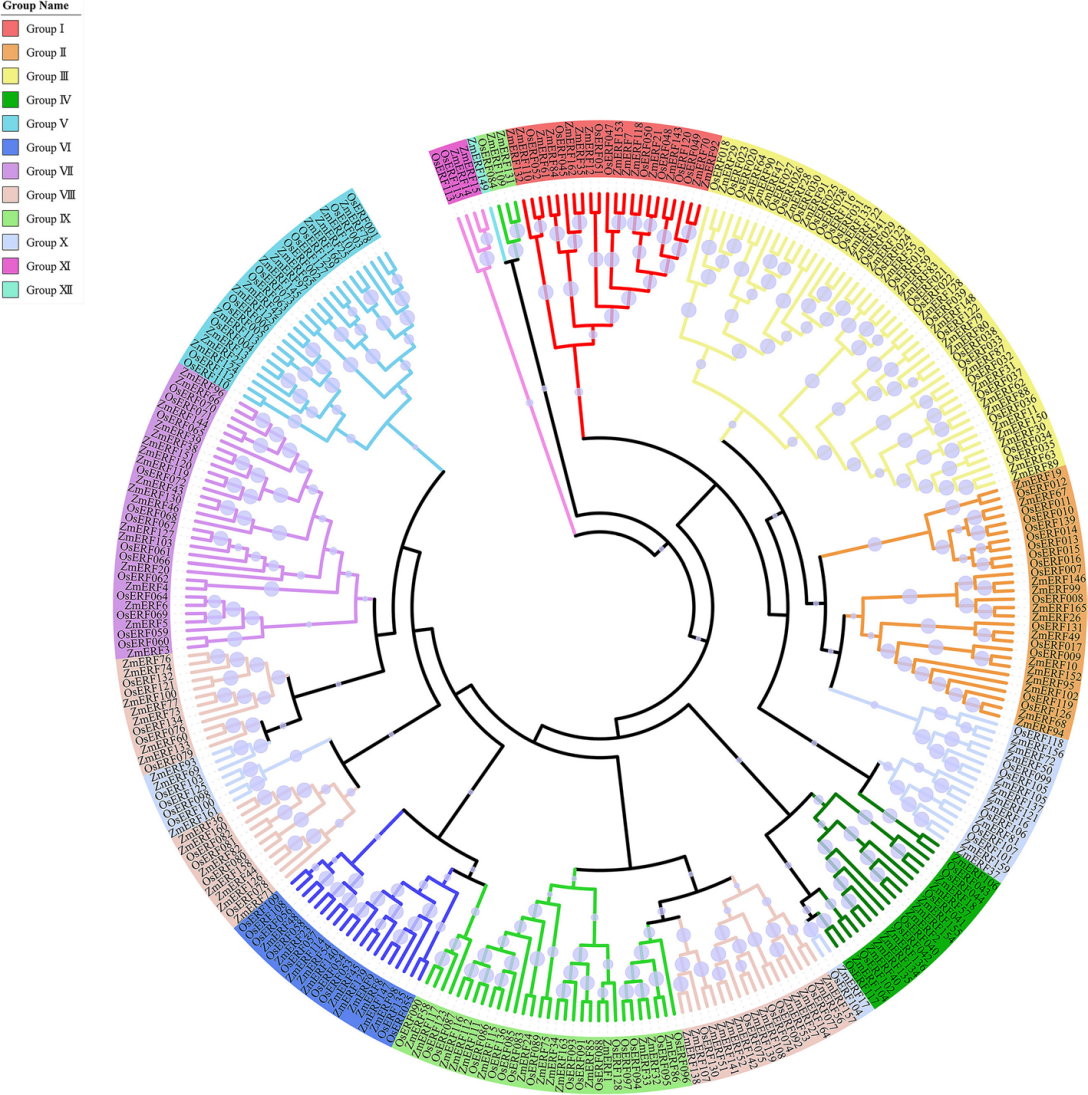
Unrooted phylogenetic tree of ERF transcription factors in maize and rice. Deduced full-length amino acid sequences from maize and rice were used to construct the phylogenetic tree using MEGA 7.0 software by a neighbor-joining method with bootstrap replicates of 1000. Twelve groups are highlighted in different colors. Bootstrap values were showed by dots with different size

To clarify the phylogenetic relationship among maize AP2/ERF superfamily proteins and obtain more detailed classification of the ERF family proteins, two unrooted phylogenetic trees including all of the AP2/ERF superfamily proteins in maize and partially representative AP2/ERF proteins published in *Arabidopsis* and rice, and the ERF family proteins from maize and rice were constructed, respectively. Using the full-length sequences of all ZmAP2/ERF proteins and representative AP2/ERF proteins from *Arabidopsis* (31) and rice (8), we firstly performed multiple sequence alignment using the MEGA 7.0 program and then constructed an unrooted phylogenetic tree through the Neighbor-Joining method (Fig. 1). Likewise, another unrooted phylogenetic tree of the ERF family proteins from maize and rice was also constructed with the same method as described above for further detailed classification (Fig. 2).

Based on the alignment of full-length protein sequences from 31 AtAP2/ERFs, 8 OsAP2/ERFs, and 214 ZmAP2/ERFs, these proteins were categorized into three major families, namely the AP2 family, the ERF family (including the ERF and DREB subfamilies) and the RAV family (Fig. 1), which was in accord with the above classification. Further, the phylogenetic tree of the ERF proteins from maize and rice was also constructed and shown in Fig. 2. According to an innovative classification for *Arabidopsis* and rice proposed by the previous study [9], the ERF family with two diverse subfamilies including the ERF and DREB subfamilies was further subdivided into 12 groups on the basis of the existing domains instead of the AP2/ERF DNA-binding domains. The DERF subfamily was consisted of four groups (Group I-IV), with each group containing respective 14, 13, 25 and 10 members, while the remaining eight groups (Group V-XII) were clustered into the ERF subfamily, including 13, 11, 18, 27, 18, 15, 2 and 1 members, respectively.

### Gene structure analysis of maize AP2/ERF genes

To analyze the exon-intron organization of individual *ZmAP2/ERF* genes, the genetic structure mapping of these *ZmAP2/ERF* genes was performed by the online software: GSDS (Gene Structure Display Server) (http://gsds.cbi.pku.edu.cn/). The *ZmAP2/ERF* genes were classified into three major families (the ERF, RAV and AP2 families) clearly based on the unrooted tree. As shown in Fig. S2, the members within the same family possess the similar exon-intron structure. In the ERF family, most of these *ZmERF* genes have no intron in their open reading frame regions. As shown in Fig. S2A, thirty-four *ZmERF* genes have 1–3 introns. Most of them belong to the Groups V, VII and X, with the position of intron being conserved in each group. The AP2 family genes containing at least three exons have more introns than *ZmERF* and *ZmRAV* genes (Fig. S2B). In contrast, the RAV family genes have no intron except *ZmRAV2* containing one intron (Fig. S2B). These results further validated the reliability of phylogenetic trees. Noticeably, genetic diversity among maize AP2/ERF superfamily genes indicated that a large differentiation might have taken place in the course of evolution of the maize genome. Furthermore, to further confirm the reliability of the AP2/ERF superfamily classification, we have performed the comparison analysis of gene structure of representative AP2/ERF genes of each different group of the AP2/ERF superfamily in maize and their orthologs in *Arabidopsis*, rice and sorghum. The results further revealed that the AP2/ERF superfamily genes share similar characteristics of gene structure either in dicots or in monocots (Fig. S3), suggesting their functional similarity among the same families from different species.

### Analysis of conserved motifs of maize AP2/ERF proteins

In addition to the AP2/ERF domain, the ZmAP2/ERF proteins also contain some group-specific conserved motifs, which may play an important role in protein-protein interactions, nuclear localization and transcriptional activities [28, 29]. Therefore, to further uncover the characteristic regions of ZmAP2/ERF proteins, the conserved motifs of 214 ZmAP2/ERF proteins were investigated using MEME web. A total of 25 conserved motifs were revealed and designated as Motif 1–25 (Fig. S4 and Table S2). Among them, the Motif − 1, 2, 3, 4, 5 and 13 were confirmed to correspond to the AP2/ERF domain, while the remaining 19 motifs were exhibited to be located in the regions outside the AP2/ERF domain, which were considered to play an important role in nuclear localization or transcription regulation (Fig. S4). It was worth noting that similar motifs outside the AP2/ERF domain were shared by the proteins within the same group. For example, the Motif-6 was exhibited in five AP2 family members. The Motif-22 specifically existed in each RAV family member and the Motif-14 was present in the Group I as well as the Motif-25, 12, 23, 15 and 21 were detected in the Groups III, IV, VI, VII and VII members, respectively. Few motifs were revealed in more than one group. For example, the Motif-17 was shared by almost all the proteins of the Group IV except for ZmERF70 in the GroupI. In addition, the Motif-20 was only found in two groups (the Groups V and IX). The Motif-10 was mainly revealed in the Groups II, III, V and X. The Motif-24 was mainly detected in the Group VII. This phenomenon might be resulted from smaller rearrangement during recombination and evolution. These results were consistent with the classification of maize AP2/ERF proteins as mentioned above. Taken together, the existence of characteristic conserved motifs of the AP2/ERF superfamily proteins further confirmed the classification of ZmAP2/ERF proteins in maize and that the proteins within the same group generally share the similar composition of conserved motifs, which might perform the similar functions.

### The cis-elements in the promoters of maize AP2/ERF genes

In order to further investigate the potential regulatory function among *ZmAP2/ERF* genes in response to abiotic or biotic stresses, the 2 Kb promoter sequences of *ZmAP2/ERF* genes were analyzed to detect cis-regulatory elements (CREs). A total of nineteen stress- and hormone-related cis-regulatory elements were revealed in promoter regions of *ZmAP2/ERF* genes including thirteen hormone-related elements (ABRE, G-Box, CGTCA-motif, TGACG-motif, TCA-element, TGA-box, TGA-element, AuxRR-core, GARE-motif, P-box, TATC-box, ERE and ARE) and six stress-related elements (TC-rich repeats, W box, MBS, LTR, WUN-motif and CCAAT-box) (Fig. S5). The ABRE (abscisic acid responsiveness) element was identified in 173 *ZmAP2/ERF* genes, while the CGTCA-motif (MeJA-responsiveness) and TGACG-motif (MeJA-responsiveness) were revealed in 175 *ZmAP2/ERF* genes, respectively. Furthermore, there were 151, 100, 41, 62, 96, 55, 103, 104, 52 and 84 *ZmAP2/ERF* genes containing G-Box (abscisic acid and light responsiveness), TGA-element and TGA-box (auxin-responsive element), TC-rich repeats (defense and stress responsiveness), TCA-element (salicylic acid responsiveness), W-box (wounding and pathogen responsiveness), and GARE-motif (gibberellin-responsive element), LTR (low-temperature responsiveness), MBS (drought inducing element), WUN-motif (injury inducing element), and CCAAT-box (high temperature response element), respectively. These results clearly suggested that the maize AP2/ERF superfamily genes might participate in regulating abiotic or biotic stress responses as well as hormone signaling transduction pathways.

### Analyses of chromosomal location, gene duplication and genome synteny

**Fig. 3:**
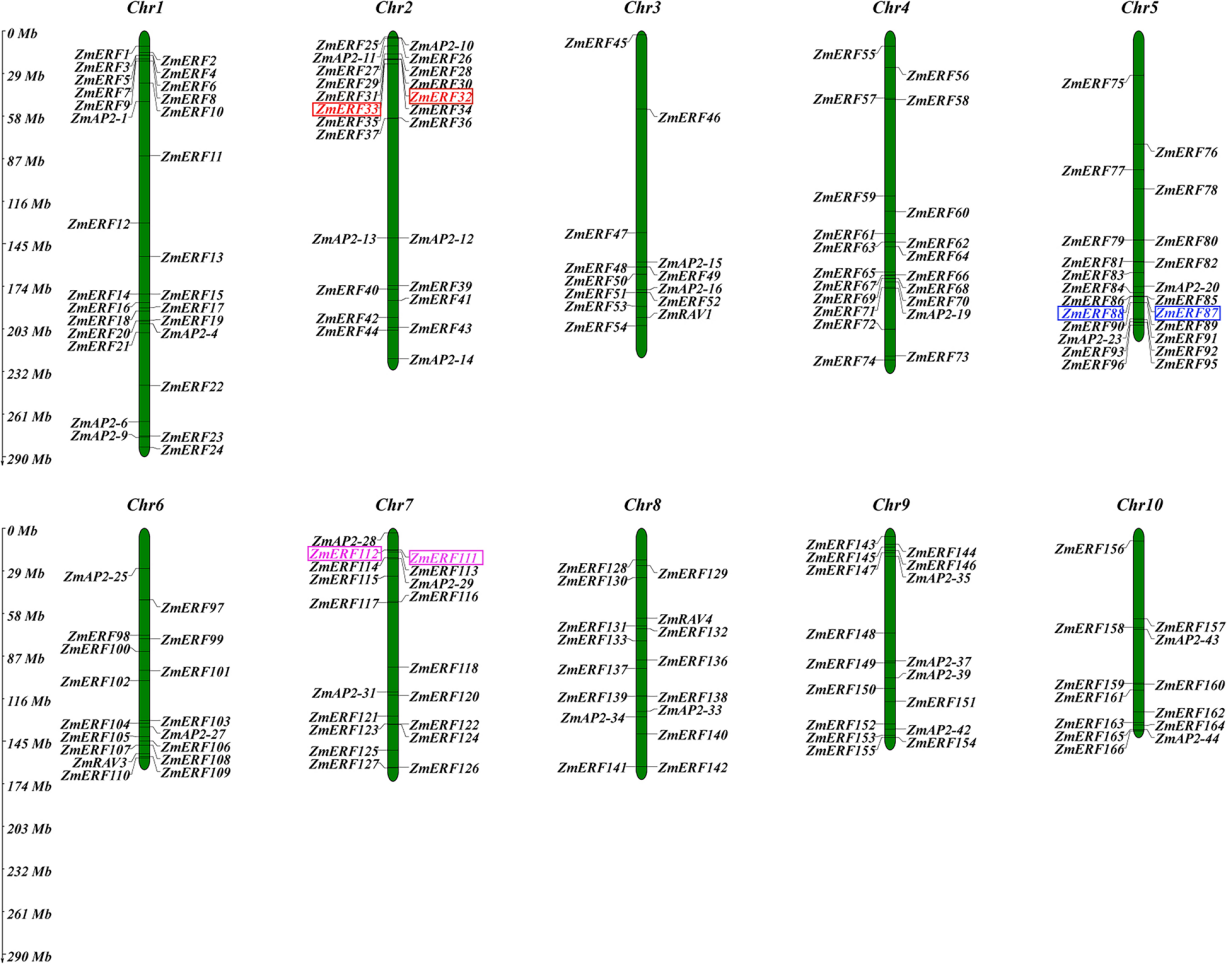
Chromosomal localization of maize *AP2/ERF* genes. Distribution of *ZmAP2/ERF* genes in maize chromosomes. 214 *ZmAP2/ERF* genes were mapped on the ten maize chromosomes. The chromosome numbers are indicated at the top of each vertical green bar. The gene names on each chromosome correspond to the approximate locations of each *ZmAP2/ERF* genes. The tandem duplicated gene pairs are indicated within boxes. The scale on the left is in megabases

To generate the graphics of chromosomal positions of *ZmAP2/ERF* genes, the physical locations of *ZmAP2/ERF* genes were investigated by analysis of genomic distribution on chromosomes. The result revealed that all 214 *ZmAP2/ERF* genes were unevenly distributed on all 10 chromosomes in maize (Fig. 3). These genes were distributed in all regions on a single chromosome (i.e. at telomeric ends, near centromere and other regions) individually or in clusters. The Chromosome 1 possesses the largest number (33) of *ZmAP2/ERF* genes, whereas the Chromosomes 3 and 10 shares the smallest number (13) of *ZmAP2/ERF* genes, and the remaining chromosomes were exhibited to contain about 19–25 *ZmAP2/ERF* genes, respectively. Moreover, there were some *ZmAP2/ERF* genes with similar conserved sequences distributed in the same chromosome. This phenomenon has been elucidated in other species, such as *A. thaliana* [18] and Chinese cabbage genomes [30], which were believed to be homologous fragments resulted from ancestral polyploidy events.

**Fig. 4:**
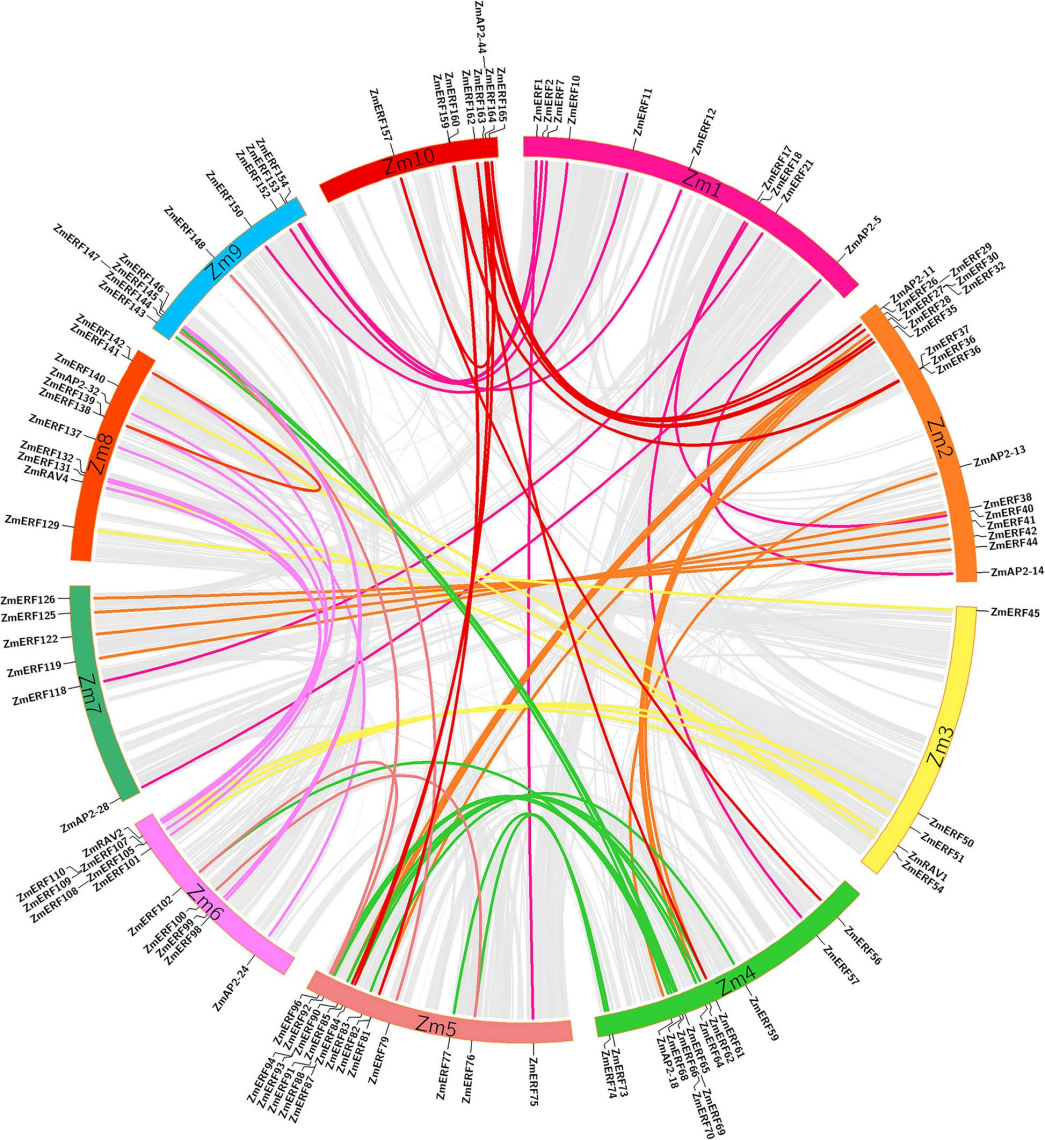
Distribution of segmentally duplicated *ZmAP2/ERF* genes on maize chromosomes. Grey lines indicate collinear blocks in whole maize genome, and the colorful lines indicate duplicated *ZmAP2/ERF* gene pairs

**Fig. 5:**
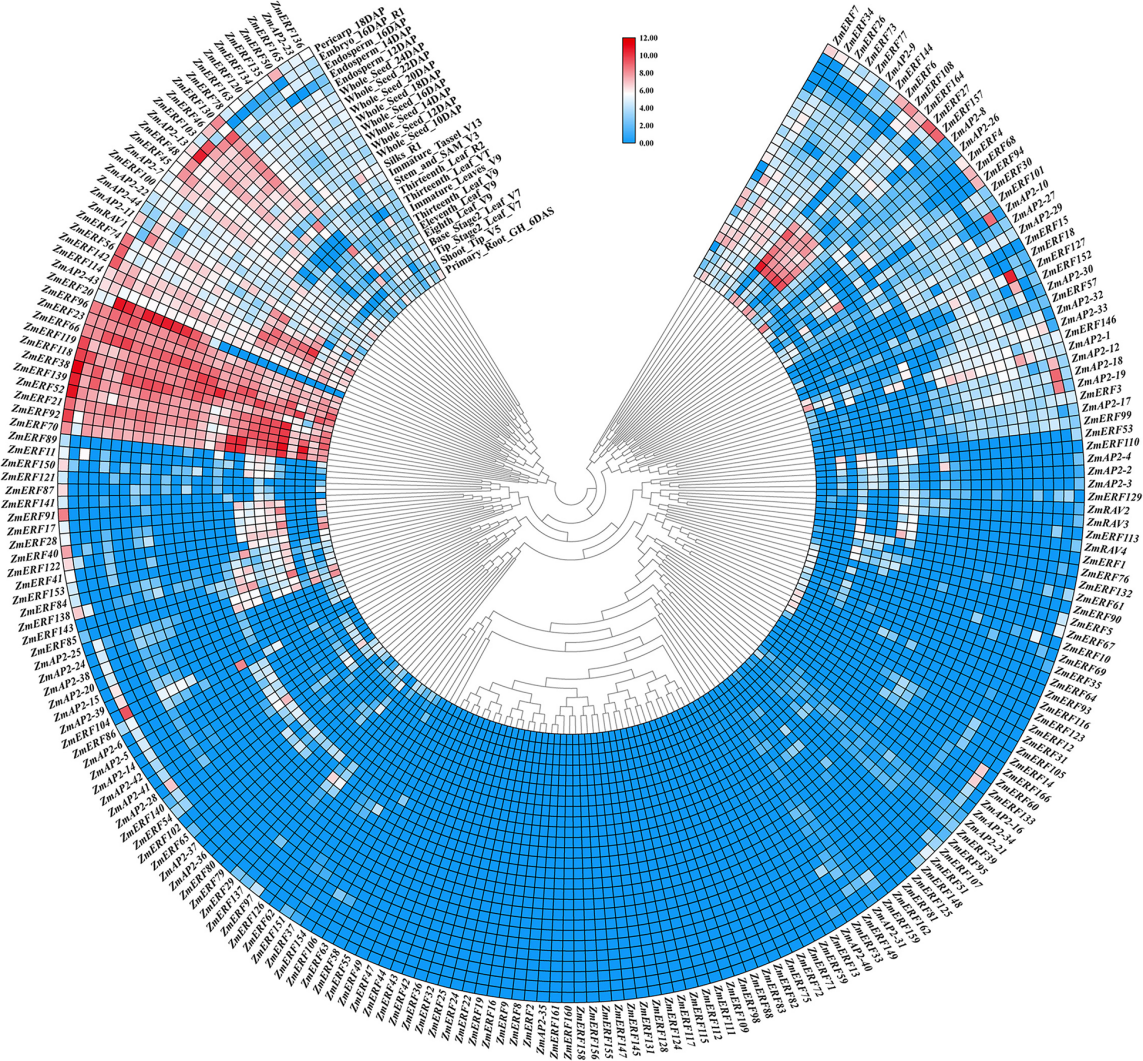
Expression profiles of *ZmAP2/ERE* genes in various tissues. Twenty-six tissues from different developmental stages including Primary_Root_GH_6DAS, Shoot_Tip_V5,Tip_Stage2_Leaf_V7,Base_Stage2_Leaf_V7, Eighth_Leaf_V9, Eleventh_Leaf_V9, Thirteenth_Leaf_V9,Immature_Leaves_V9,Thirteenth_Leaf_VT,Thirteenth_Leaf_R2,Stem_and_SAM_V3,Immature_Tassel_V13,Silks_R1,Whole_Seed_10DAP,Whole_Seed_12DAP,Whole_Seed_14DAP,Whole_Seed_16DAP,Whole_Seed_18DAP, Whole_Seed_20DAP, Whole_Seed_22DAP, Whole_Seed_24DAP,Endosperm_12DAP,Endosperm_14DAP,Endosperm_16DAP,Embryo_16DAP_R1, Pericarp_18DAP were investigated. Scale bars represent log2 of the RPKM values

Furthermore, due to important effect on functional differentiation and gene expansion, gene duplication events among *ZmAP2/ERF* genes in the maize genome were also investigated. According to the previous studies, chromosomal regions within 200 Kb range including two or more genes were designated as tandem duplication [31]. In this study, three pairs of tandem duplicated genes were revealed and distributed on the Chromosomes 2, 5 and 7, respectively (Fig. 3). The length of regions between these tandems duplicated genes varied from 43.7 Kb to 166.7 Kb. One tandem duplicated gene pair (*ZmERF87* and *ZmERF88*) distributed on the Chromosome 5 was assigned to the Group IX, while two other gene pairs were classified into the Group III together. Furthermore, seventy-nine putative segmental duplicated gene pairs among *ZmAP2/ERF* genes in the maize genome were further confirmed through the genome-wide synteny analysis as shown in Fig. 4. In addition, the orthologous relationships of *AP2/ERF* genes between maize and two other gramineous species (rice and sorghum) were also investigated to explore the origin and evolutionary relationships among *AP2/ERF* genes using the same method (Fig. 5A and B), respectively. Through the genome-wide syntenic analyses, 80% (153) and 86% (163) of *ZmAP2/ERF* genes in maize were confirmed to share at least one or several putative orthologs in rice and sorghum, respectively. The extensive synteny among maize, rice and sorghum supported their close evolutionary relationship among three gramineous species. What’s more, most *ZmAP2/ERF* genes were revealed to exhibit syntenic bias towards particular chromosomes in rice and sorghum (Fig. S6), suggesting that the chromosomal rearrangement events such as inversion and duplication might primarily affect the organization and distribution of *AP2/ERF* genes in these genomes. Therefore, these comparative mapping figures offered valuable information for comprehending the evolution of *AP2/ERF* genes among different gramineous species including maize (Fig. 4 & S6). Moreover, these results will contribute to selecting candidate *ZmAP2/ERF* genes and thereby facilitate genetic improvement for the members of this superfamily. For instance, the gene FZP (LOC_Os07g47330) has been demonstrated to be essential for floral meristem in rice [32]. Thus, it is likely that its orthologous genes (*ZmERF44* and *ZmERF126*) may also be involved in similar function in maize.

Besides, no synonymous substitutions (*Ka*), synonymous substitutions (*Ks*) and *Ka*/*Ks* ratio of each duplicated *ZmAP2/ERF* gene pair were calculated to unravel selective constraints on duplicated *ZmAP2/ERF* genes. Generally, a duplicated gene pair with a *Ka*/*Ks* ratio > 1 is confirmed to be evolved under positive selection, *Ka*/*Ks* = 1 stands for neutral selection, while *Ka*/*Ks* < 1 mean negative or purifying selection. For segmental duplicated gene pairs in maize *AP2/ERF* genes, the *Ka/Ks* ratio ranged from 0.20 to 0.92 with an average of 0.48, whereas the *Ka/Ks* ratio for tandem duplicated gene pairs in maize *AP2/ERF* genes varied from 0.43 to 0.57 with an average of 0.49 (Tables S3, S4). These results showed that all the *Ka*/*Ks* ratios of these duplicated *ZmAP2/ERF* gene pairs were estimated to be less than one, indicating that these genes might be under strong purifying selection pressure and experience substitution elimination and enormous selective constraint by natural selection during the process of evolution. Furthermore, it was supposed that the duplication event of these *ZmAP2/ERF* tandem and segmental duplicated genes might take place at ~ 47 and ~ 39 Mya (Tables S3, S4), respectively. Even though the *ZmAP2/ERF* gene pairs of tandem duplication (*Ka*/*Ks* = 0.49) and segmental duplication (*Ka*/*Ks* = 0.48) events were not under similar evolutionary positive selection pressure, both of them showed that these duplication events might occur simultaneously. In addition, we also calculated the *Ka*/*Ks* ratios of orthologous gene pairs between maize and two other gramineous species (Tables S5, S6). The average *Ka*/*Ks* value was maximum between maize and rice (0.43), followed by sorghum (0.42), indicating that it’s seemed that both rice-maize and sorghum-maize orthologous gene pairs have experienced extensive intense purifying selection. The divergence time for rice and sorghum was about 47 and 31 Mya, respectively (Tables S5, S6). Thus, it can be inferred that tandem or segmental duplication events might exert a main impact on functional differentiation and evolution of the AP2/ERF superfamilies in gramineous species.

### Expression profiles of maize AP2/ERF genes in different tissues

Based on maize transcriptome data released by the previous study [33], the expression profiles of 214 *ZmAP2/ERF* genes in 26 tissues were accomplished to further investigate their potential function during the process of growth and development in maize. The results showed the expression levels among *ZmAP2/ERF* genes were highly varied (Fig. 5). Among these 214 *ZmAP2/ERF* genes, the expression levels were examined in at least one tissue for 173 genes, whereas 41 genes were not detected in any tissue. There were 33 genes expressed in all tested tissues, even though some of them did not show high expression levels. To further investigate the transcription profiles of *ZmAP2/ERF* genes, their expression patterns were clustered together across all the tested tissues as shown in Fig. 5. Among these *ZmAP2/ERF* genes, 11 genes were expressed at relatively high levels in all tissues and stages investigated, including the members of the Group I (*ZmERF70*, *ZmERF92*, *ZmERF21* and *ZmERF118*), the Group VI *(ZmERF23*), the Group VII (*ZmERF38*, *ZmERF119*, *ZmERF66* and *ZmERF96*) and the Group VII (*ZmERF52* and *ZmERF139*), implying that they were involved in maize growth and development regulation. What’s more, some important candidate genes expressed highly or specifically in some tissues were also identified. For instance, *ZmAP2–10* and *ZmERF127* were detected to be predominantly expressed in embryo and endosperm at 16 days after pollination, respectively, while *ZmERF154* was exhibited to show a specific high expression level in silks compared with other tissues. Furthermore, sixteen genes, namely *ZmAP2–1*, *ZmAP2–10*, *ZmAP2–12*, *ZmAP2–13*, *ZmAP2–17*, *ZmAP2–18*, *ZmAP2–19, ZmERF3*, *ZmERF14*, *ZmERF15*, *ZmERF18*, *ZmERF20*, *ZmERF57*, *ZmERF104*, *ZmERF127 and ZmERF149*, showed preferential expression during pollination, which may be involved in regulating the development of entire seed. Moreover, 18 *ZmAP2/ERF* genes, namely *ZmAP2–2*, *ZmAP2–3, ZmAP2–4*, *ZmERF6*, *ZmERF17*, *ZmERF28*, *ZmERF41*, *ZmERF61*, *ZmERF64*, *ZmERF76*, *ZmERF84*, *ZmERF89*, *ZmERF90*, *ZmERF91*, *ZmERF110*, *ZmERF122*, *ZmERF143* and *ZmERF153*, were also identified to be predominantly expressed in the course of leaf development.

### Weighted gene co-expression network and protein protein interaction network analysis

**Fig. 6:**
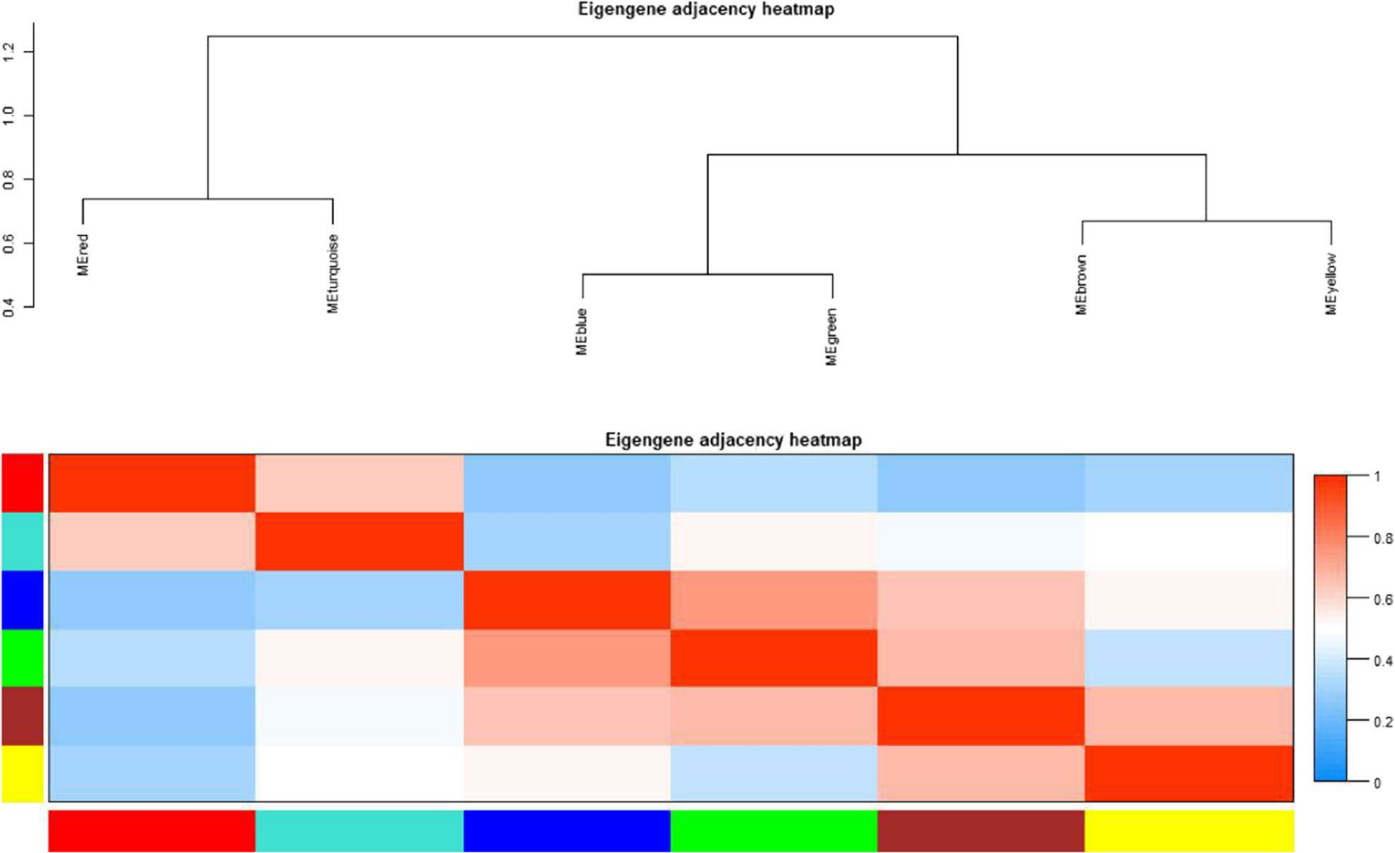
Co-expression network analysis of *ZmAP2/ERF* genes. Elgengene adjacency heatmap. A high degree of correlation between modules is indicated by red. (For interpretation of the references to color in this figure legend, the reader is referred to the web version of this article)

Based on the analysis of expression profiles, we further explored gene association patterns among members of the AP2/ERF superfamily in maize and identified highly associated co-expressed genes. The normalized FPKM (Fragments per thousand base transcripts per million labeled readings) expression values of *ZmAP2*s (32 genes), *ZmRAV*s (4 genes) and *ZmERF*s (158 genes) were used as input data to construct the co-expression network, while they were in different tissues. The results showed that there were six modules divided with different colors, for which contained various *ZmAP2/ERF* genes, respectively (Fig. 6). In these modules, the turquoise module (20 genes) is larger than any other module, followed by the blue module (19 genes), the brown module (15 genes), the yellow module (12 genes), the green module (8 genes) and the red module (7 genes). The correlation among these modules was shown in Fig. 6. Due to the similarity of expression patterns between two modules in differentiated tissues, the green module and the blue module are closely related than other modules. Each module was revealed to show special genes and expression patterns, which suggested different interactions among members of the AP2/ERF superfamily, and might be responsible for regulating various growth and developmental processes in maize.

**Fig. 7:**
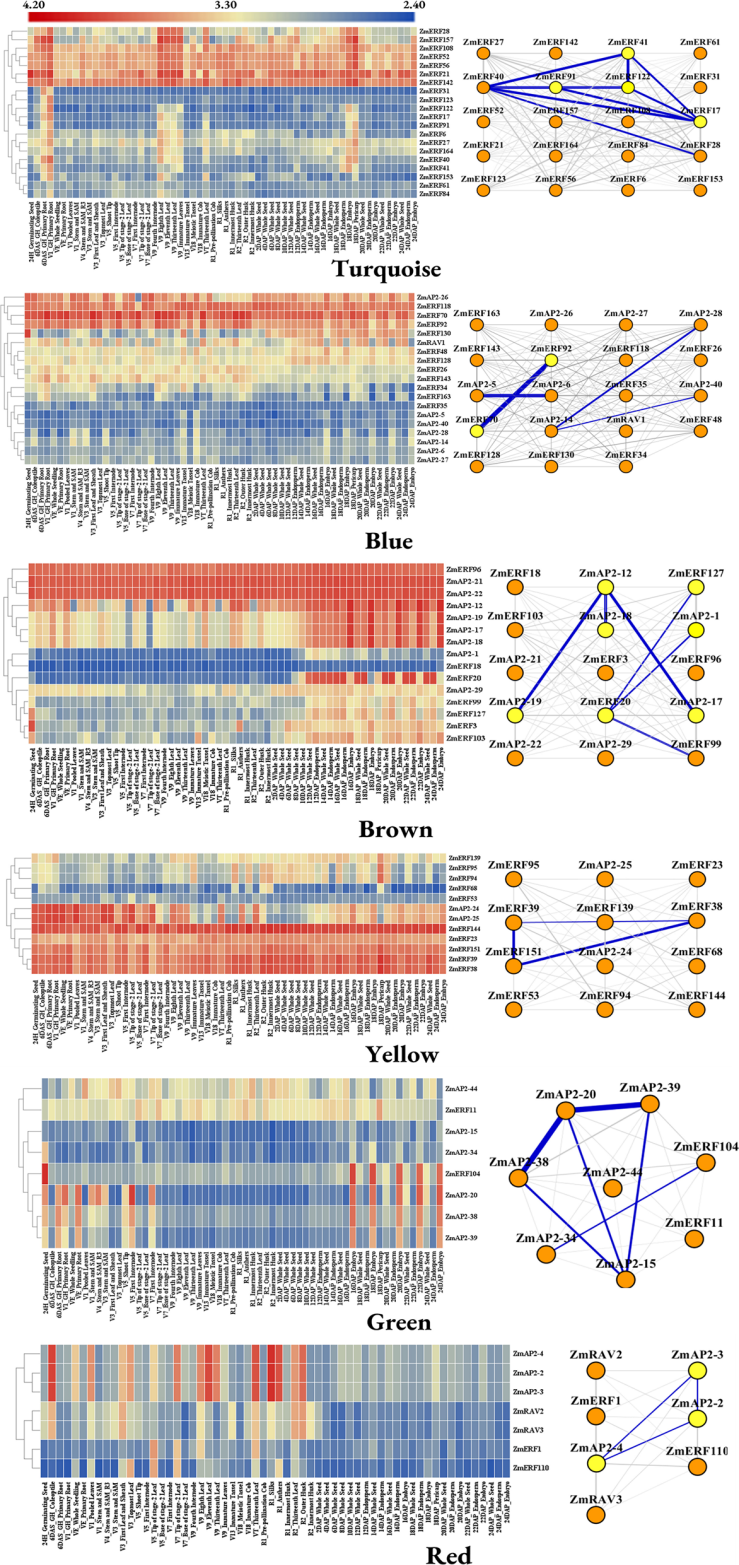
Heat map and topological map of gene expression profile in the module. **A** Turquoise module (**B**) blue module (**C**) brown module (**D**) yellow module (**E**) green module (**F**) red module. In the heat map, blue indicates low expression and red indicates high expression. The black line in the topological graph indicates the interaction between genes, and the thicker the line is, the deeper the relationship is; the ellipse represents genes, and the color represents the score of genes

Previous studies have shown that many genes encoding AP2/ERF transcription factors were generally responsive to various abiotic stresses during the plant growth and development [34]. As shown in Fig. 7, gene association patterns for each module in multiple tissues and at different stages of growth were further revealed. In the turquoise module, there were four genes (*ZmERF17*, *ZmERF41*, *ZmERF91* and *ZmERF122*) revealed to be highly interlinked and specifically involved in leaf development. Five genes (*ZmERF21*, *ZmERF52*, *ZmERF56*, *ZmERF108* and *ZmERF142*) in the module were expressed at higher levels than other genes, which implied that they might play a role in the whole process of growth and development in maize. In the blue module, *ZmERF70* was shown to have a strong co-expression relationship with *ZmERF92*, which shared a similar relationship between *ZmAP2–5* and *ZmAP2–6*. Nearly half of the genes in the brown module were expressed at higher levels in endosperm, embryo and seed, but lower in other tissues. In the yellow module, five genes (*ZmERF23*, *ZmERF38*, *ZmERF39* and *ZmERF151*) was revealed to be active in transcription, and among them, the expression level of *ZmERF144* was the highest. The green module contained only two genes encoding ERF transcriptional factors (*ZmERF11* and *ZmERF104*), and six related genes encoding AP2 transcriptional factors, including *ZmAP2–15*, *ZmAP2–20*, *ZmAP2–34*, *ZmAP2–38*, *ZmAP2–39* and *ZmAP2–44*. Half of the genes in the green module were highly expressed exclusively in embryo. The expression patterns of *ZmAP2–2*, *ZmAP2–3* and *ZmAP2–4* were closely related in the red module and shown to share high expression levels in coleoptile, leaf and husk.

**Fig. 8:**
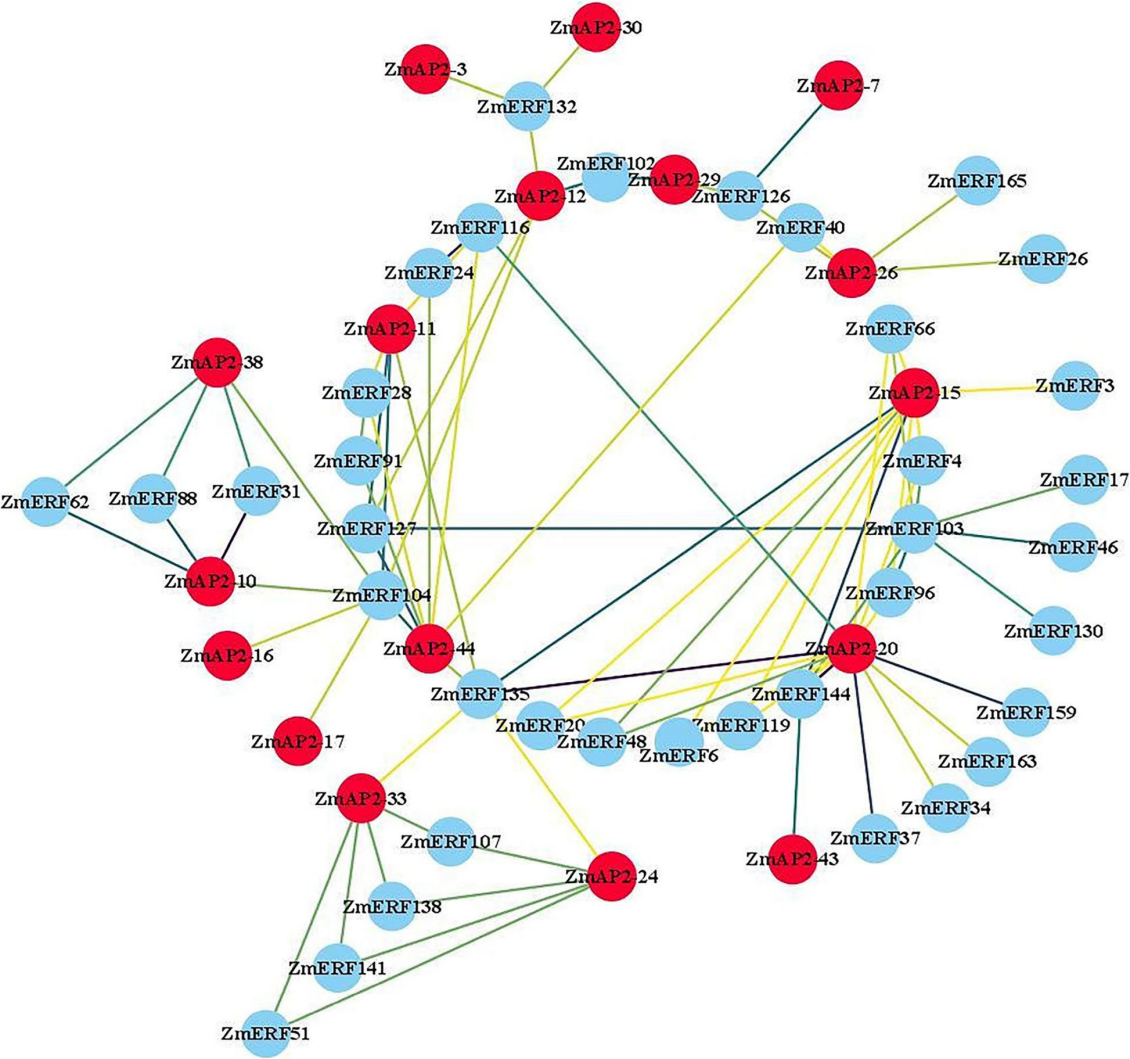
Protein Protein Interaction network between ZmAP2 and ZmERF proteins. Blue and red color nodes represent ZmERF and ZmAP2 proteins, and the deeper lines represent the higher interaction, the lighter the color represents the lower interaction. (For interpretation of the references to color in this figure legend, the reader is referred to the web version of this article)

Previous studies have demonstrated that the AP2, ERF and RAV proteins individually play important roles in plant organ development [12, 35], hormone-signaling responses [36], biotic and abiotic stress responses, respectively [17]. In this study, a PPI network analysis was also performed to reveal the interaction between ZmAP2 and ZmERF proteins (Fig. 8). The result showed that a closely related PPI network was constructed between 17 ZmAP2 and 37 ZmERF proteins, suggesting that these proteins might be involved in some regulatory networks associated with ZmAP2s and ZmERFs complex. Among them, there were two proteins, namely ZmAP2–24 and ZmAP2–33 revealed to interact with ZmERF51, ZmERF107, ZmERF135, ZmERF138 and ZmERF141. Likewise, another ZmAP2–10 and ZmAP2–38 were revealed to interact with ZmERF31, ZmERF62, ZmERF88 and ZmERF104. Interestingly, more than eight ZmERF factors were also revealed to interact with ZmAP2–11, ZmAP2–15, ZmAP2–20 and ZmAP2–44. In addition to interaction with ZmAP2 proteins, some ZmERF proteins such as ZmERF103 were also found to interact with ZmERF17, ZmERF46, ZmERF127 and ZmERF130. These results indicated that they might be involved in crosstalk of diverse signal transduction pathways through the interaction between ZmAP2 and ZmERF proteins.

### Expression profiles of maize AP2/ERF genes under heat stress treatment

**Fig. 9:**
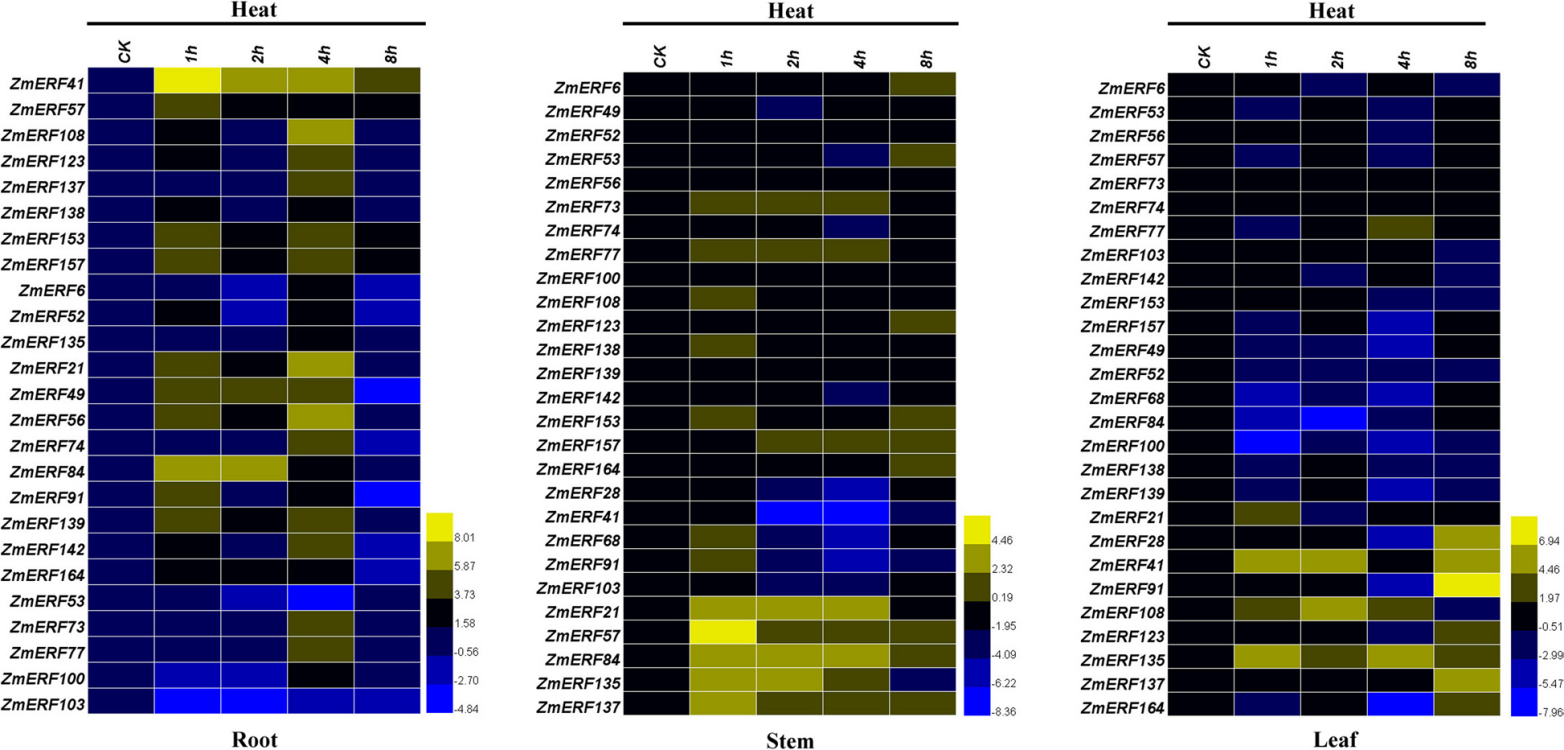
Expression profiling of the 27 selected *ZmAP2/ERF* genes under heat stress treatment in roots, stems and leaves. QRT-PCR data was normalized using maize *ZmActin1* gene and are shown relative to CK (normal condition). CK (0 h), the control condition and Heat (1 h, 2 h, 4 h and 8 h), the heat stress conditions. Color bar in the right indicates value of gene expression potential.

Previous studies have shown that the DREB subfamily genes played a critical role in responding to abiotic stresses, while those of the ERF subfamily participated in the regulation of responses to biotic or abiotic stresses [6]. Therefore, in this study, a total of 27 genes were randomly selected from these two subfamilies to reveal their expression patterns in maize after three diverse abiotic stress treatments. Firstly, the expression levels of these 27 *ZmAP2/ERF* genes in roots, stems and leaves of maize sampled at five different time points (0, 1, 2, 4 and 8 h) after heat stress treatment were investigated through quantitative real-time PCR, respectively (Fig. 9). At least three biological replicates were accomplished to make sure the reliability of quantitative analysis.

After heat stress treatment, these 27 investigated genes were confirmed to show differential accumulation. Interestingly, the majority of these *ZmAP2/ERF* genes exhibited differential up-regulated expression patterns in three different tissues under heat stress treatment compared with those under normal condition (CK), indicating that these genes may play important roles in the response to heat stress. Among them, only six genes (*ZmERF41*, *ZmERF49*, *ZmERF52, ZmERF56, ZmERF100* and *ZmERF103*) were revealed to show down-regulated expression levels in three diverse tissues under heat stress treatment, respectively. Although most of these 27 *ZmAP2/ERF* genes shared the same up-regulated expression patterns in roots, stems and leaves, they were revealed to exhibit significantly differential expression levels between different tissues at different time points after heat stress treatment (Fig. 9). For instance, in roots, there were at least nineteen *ZmAP2/ERF* genes that were significantly up-regulated at more than 10-fold greater levels after heat treatment, whereas only three *ZmAP2/ERF* genes in stems and eight *ZmAP2/ERF* genes in leaves were significantly up-regulated at more than 10-fold greater levels after heat treatment compared with those in roots, respectively. Furthermore, under heat treatment, most of these genes exhibited more rapid and much stronger responses in roots than those in stems or leaves. For example, in roots, eleven *ZmAP2/ERF* genes were rapidly induced and peaked at more than 10-fold greater levels at 1 h after heat treatment, whereas only three *ZmAP2/ERF* genes in stems and four *ZmAP2/ERF* genes in leaves were revealed to exhibit the same expression patterns compared with those in roots, respectively. Taken together, this result suggested that the *ZmAP2/ERF* genes in roots might participate in plant responses to heat stress more strongly or rapidly than that in stems and leaves when respond to heat stress treatment.

### Expression profiles of maize AP2/ERF genes under drought stress treatment

**Fig. 10:**
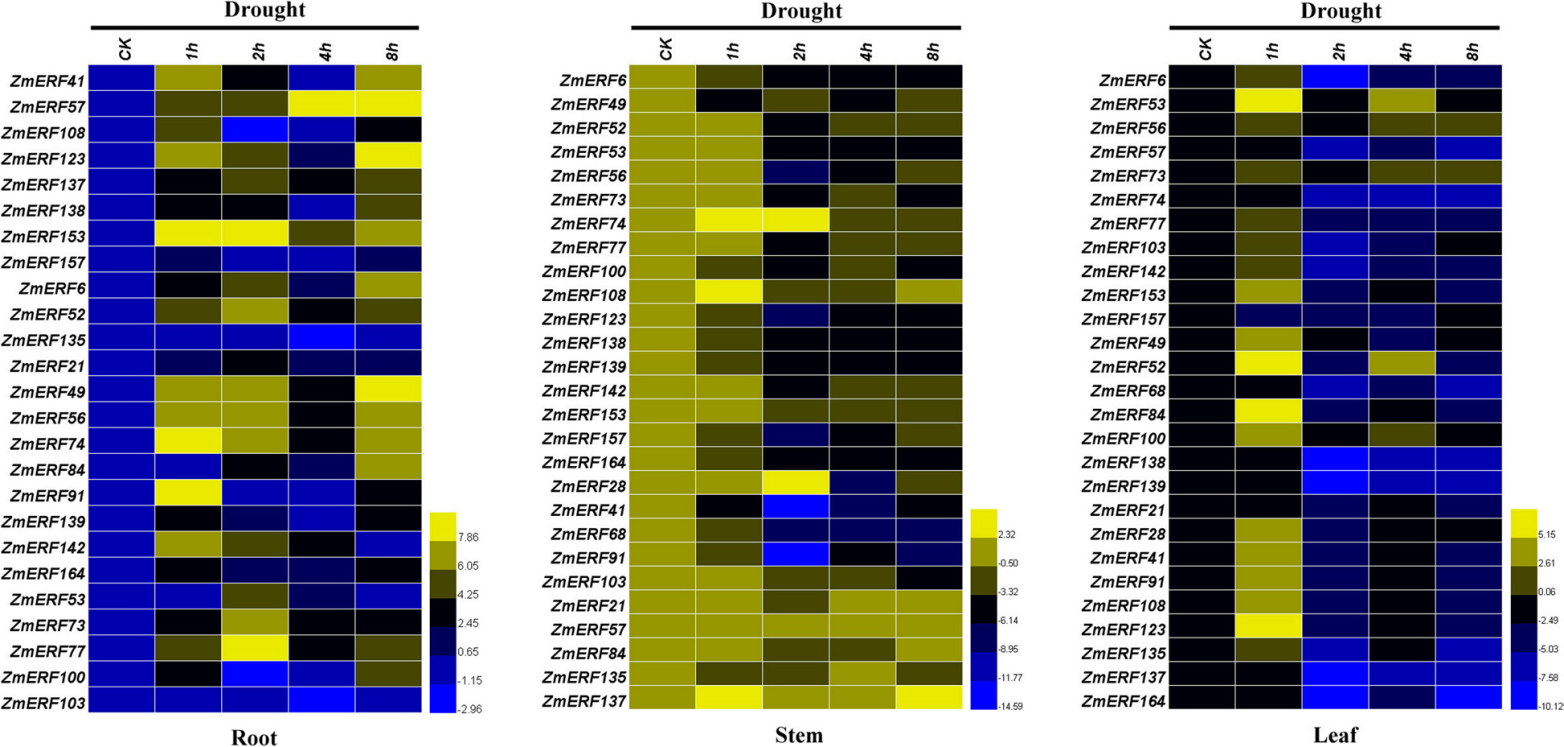
Expression profiling of the 27 selected *ZmAP2/ERF* genes under drought stress treatment in roots, stems and leaves. QRT-PCR data was normalized using maize *ZmActin1* gene and are shown relative to CK (normal condition). CK (0 h), the control condition and Drought (1 h, 2 h, 4 h and 8 h), the drought stress conditions. Color bar in the right indicates value of gene expression potential.

To further explore drought stress-responsiveness of these selected *ZmAP2/ERF* genes in maize, the seedling roots, stems and leaves were sampled at five different time points (0, 1, 2, 4 and 8 h) after drought stress treatment and carried out for the quantitative real-time PCR analysis, respectively (Fig. 10). Surprisingly, under drought stress treatment, the expression profiling of these selected *ZmAP2/ERF* genes in maize exhibited a similar result in three different tissues compared with that under heat stress treatment. The majority of these *ZmAP2/ERF* genes exhibited differential up-regulated expression patterns in three different tissues under drought stress treatment compared with those under normal condition (CK), indicating that these genes may play important roles in the response to drought stress. Especially, in roots, all these *ZmAP2/ERF* genes were shown to exhibit high expression levels after drought treatment, and the majority of them were significantly up-regulated and rapidly peaked at more than 30-fold greater levels after drought treatment. However, in stems or leaves, the expression levels of only a few *ZmAP2/ERF* genes were significantly up-regulated and peaked at more than 10-fold greater levels after drought treatment, indicating that these genes exhibited more rapid and much stronger responses in roots than those in stems or leaves when respond to drought stress treatment. Taken together, these results suggested that the *ZmAP2/ERF* genes can be induced to exhibit differential expression patterns in various tissues after drought treatment and the majority of them were immediately responsive to drought stress, especially in roots, indicating that these genes might be involved in plant responses to drought stress more strongly or rapidly than that in stems and leaves.

### Expression profiles of maize AP2/ERF genes under salt stress treatment

**Fig. 11:**
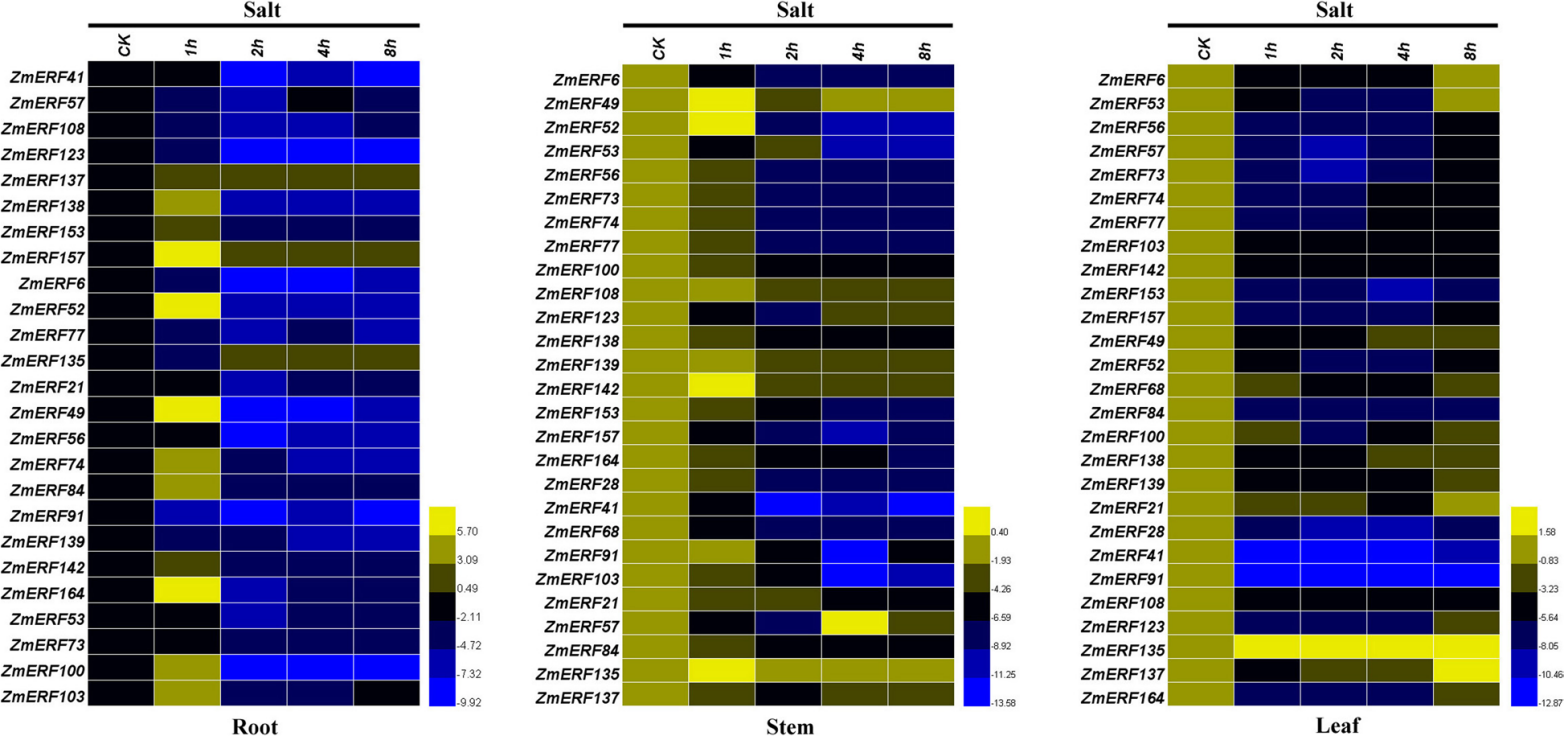
Expression profiling of the 27 selected *ZmAP2/ERF* genes under salt stress treatment in roots, stems and leaves. QRT-PCR data was normalized using maize *ZmActin1* gene and are shown relative to CK (normal condition). CK (0 h), the control condition and Salt (1 h, 2 h, 4 h and 8 h), the salt stress conditions. Color bar in the right indicates value of gene expression potential.

To further confirm molecular function of *ZmAP2/ERF* genes in response to salt stress, the expression levels of these 27 selected *ZmAP2/ERF* genes in maize roots, stems and leaves at 0, 1, 2, 4, 8 h after salt treatment (200 mM NaCl) were investigated through qRT-PCR (Fig. 11). However, under salt stress treatment, the expression levels of only nine ZmAP2/ERF genes in roots were significantly up-regulated and rapidly peaked at more than 10-fold greater levels at 1 h after drought treatment, whereas the majority of these genes were revealed to exhibit significantly down-regulated expression levels. Likewise, in maize stems and leaves, the expression levels of the majority of these genes were significantly suppressed by salt stress at all time points. In stems, the expression levels of only five genes (*ZmERF49, ZmERF52, ZmERF135, ZmERF139* and *ZmERF142*) were slightly up-regulated at 1 h and then significantly down-regulated at other time points after salt treatment compared with those under normal condition (CK). The remaining genes were significantly down-regulated at all time points after alt treatment. Also, in leaves, the expression levels of only four genes (*ZmERF6, ZmERF21, ZmERF53* and *ZmERF137*) were slightly up-regulated at 8 h after salt treatment, whereas the expression levels of the majority of the remaining genes were significantly down-regulated at all time points after alt treatment in comparison with the control in leaves. Taken together, these results showed that the *ZmAP2/ERF* genes in maize were responsive to salt stress, indicating that these genes might be widely involved in salt stress response.

## Discussion

The AP2/ERF superfamily proteins as plant-specific TFs have been demonstrated to be involved in regulating multiple biological processes including biotic and abiotic stress responses [13, 37–40]. The comprehensive investigations of the AP2/ERF superfamily genes have been accomplished in various plant species. However, few studies have been performed to investigate the AP2/ERF superfamily genes in maize. Although several recent studies have provided some information about AP2/ERF genes in maize, including the identification of 184 AP2/ERF genes in maize waterlogging stress response [41], 76 ERF subfamily genes [42] and 18 DREB subfamily genes in maize [43], the characterization of the majority of these genes in these studies was mainly resulted from the previous AGPv3 version of the maize genome. Because of the complexity of maize genome and the constant updating of maize database, a comprehensive of genome-wide identification and characterization of AP2/ERF superfamily in maize still remain to be further elucidated using a new version of the maize genome. In this study, the AP2/ERF superfamily genes in maize were comprehensively investigated using the AGPv4 version genome of the maize genome, resulting in the identification of 214 genes with complete AP2/ERF domain. Even though the maize genome size (2300Mbp) is much larger than those of *Arabidopsis* (125Mbp) and rice (389Mbp), only 214 maize *AP2/ERF* genes were identified and characterized in this study. The number of *ZmAP2/ERF* genes is similar to that of *Arabidopsis* (147) [18] or rice (152) [9]. There are many possible explanations for this phenomenon, including a large gene loss during the maize genome duplication or less gene duplications in the maize genome. Moreover, this further indicated that the evolution of the AP2/ERF superfamily genes is highly conserved among various gramineous species.

In this study, the AP2/ERF proteins were revealed to be mainly classified into three distinct families (the AP2, ERF and RAV families). Meanwhile, on the basis of the phylogenetic tree analysis of ERF transcription factors in maize and rice as mentioned above (Fig. 2), the maize ERF family proteins were further categorized into 12 groups according to the previous study [9], namely: the DREB (I-IV) and ERF (V-XII) groups. In total, two maize ERFs (ZmERF14 and ZmERF15) were clustered into the Group XI with the Group XI proteins of rice. The presence of most Groups (I-X) in three species (maize, *Arabidopsis* and rice) indicated that most of these proteins might pre-date the species divergence. Likewise, some groups only existed in only one species. For example, the Group XII was revealed to only exist in the maize ERF family, but not in *Arabidopsis* or rice ERF families, indicating that this group had evolved or been lost in some species during evolution. Furthermore, multiple sequence alignment of AP2/ERF domains in ZmERF family proteins was accomplished, indicating that Ala-14 and Asp-19 residues were conserved in AP2/ERF domains from the ZmERF subfamily proteins, while Val-14 and Glu-19 residues were conserved in AP2/ERF domains from the ZmDREB subfamily proteins. These amino acid residues were thought to be vital for the classification of these two subfamilies (the ERF and DREB subfamilies) genes [18]. A few members without these conserved amino acid residues were classified according to their position in the phylogenetic tree. Thus, the comparative analysis of amino acid residues of AP2/ERF domains in maize ERF family proteins suggested the AP2/ERF domains were well conserved. These conserved amino acid residues indicated that they might play crucial roles in involving the ERF family proteins in different forms of physical contact with DNA. According to Allen et al. [27], the AP2/ERF domain was revealed to recognize its target DNA via some conserved arginine and tryptophan residues in β-sheet. Moreover, the Ala-37 residue in α-helix might also play a crucial role in DNA binding or stability of the AP2/ERF domain [44].

The number of introns was also predicted in this study (Fig. S2). Remarkably, the number of the introns in the AP2 family genes was larger than that in the genes of two other families (the ERF and RAV families), whereas no introns were discovered in the RAV family genes except for *ZmRAV2* containing one intron (Fig. S2B), and most of the *ZmERF* genes have no or one intron in their open reading frame regions (Fig. S2A). This typical pattern of gene structure is in accord with those of the previous studies in *A. thaliana* [18], Chinese jujube [45] and grape [46]. In addition, to further confirm the reliability of the AP2/ERF superfamily classification, this investigation also showed that the AP2/ERF superfamily genes have similar characteristics either in dicots or in monocots (Fig. S3), which may be responsible for their functional similarity among the same families from different species.

Moreover, the similar motif distribution has been frequently revealed in most AP2/ERF proteins from the same group, indicating that these proteins were likely to share similar functions in plants. Therefore, the conserved motifs of ZmAP2/ERF proteins were further confirmed in this study. A total of 25 conserved motifs were investigated in the 214 ZmAP2/ERF proteins (Fig. S4). Apart from the Motif-1, Motif-2 and Motif-4, which only existed in the AP2 domain and were found in almost all AP2/ERF superfamily members, most motifs were revealed to exhibit a group-specific distribution, implying that these conserved motifs might play vital roles in function (Fig. S4). For example, the Motif-24 was consisted of an “EDLL” motif and existed in members of the Group VII. The “EDLL” motif was previously demonstrated to be a transcriptional activation domain in AP2/ERF proteins [28], suggesting its involvement in activating the function of members of the Group VII. In addition, the Motif-15 was only present in N-terminal regions of most ZmAP2/ERF proteins of the Group VII and contained MCCGAI residues, which was essential for ethylene transcriptional activation [47]. Moreover, the Motif-10 that mainly existed in members of the Groups II, III, V and X shared four conserved residue features: [L/R]AAA, LPR[P/A] and D[I/V] QAA. In *Arabidopsis*, they have been demonstrated to serve as the essential functional motifs for Auxin response factor-19, CBL-interacting serine/threonine-protein kinase-12 and Ethylene-responsive transcription factor ERF037, respectively [48]. The Motif-14 was specific in proteins of the Group Iand contained four more conserved residues (LDF[S/T] E, APWDE, KYPS and EIDWD). Previous studies have demonstrated the importance of LDF[S/T] E residue in disease resistance [49]. The APWDE residue has also been identified to be involved in transcriptional regulation and mediating the acetylating of histone H3 and H4 of target loci [50]. The K[Y/F] PS residue has been revealed to be involved in DNA methylation during plant development to regulate gene expression [51]. The EIDWD residue has been involved in the ethylene response in *A. thaliana* [52]*.* Furthermore, the cytokinin responsive factor (CRF) domain has been revealed in the Motif-23 and only existed in the CRF proteins of the Group VI [53]. In addition to these commonly conserved motifs discovered in *Arabidopsis* and rice, some maize-specific motifs of the AP2/ERF superfamily proteins have been revealed in this study, which may play a pivotal role in biological regulation of maize. However, the function on these motifs still remains to be further elucidated, and more work should be done to explore their regulatory functions. In summary, these results suggested that the occurrence of these conserved motifs might play a crucial functional role in evolutionarily diverse organisms, while maize specific motifs might have been evolved later to meet specific functions.

The ERF family genes have been widely illustrated to be involved in hormonal stimuli responses and developmental processes regulation in various plants. At the same time, some members of the AP2 family have also been illuminated to play an important role in regulating developmental processes including meristem determinacy and flower development [54]. Therefore, to further reveal underlying functions of the maize AP2/ERF superfamily members during plant growth and development processes, the expression patterns of 214 *ZmAP2/ERF* genes were systematically analyzed in 26 different tissues and organs according to microarray data. As shown in Fig. 5, the heat map revealed that 173 *ZmAP2/ERF* genes were expressed in at least one investigated tissue, suggesting that they were widely involved in growth and development. Compared with the AP2 family members, the ERF family genes were revealed to show higher expression levels in these tissues, which might be resulted from the fewer intron content of the ERF family. Due to the small number of introns, the ERF family genes can respond more quickly and exhibit higher expression levels during development. Meanwhile, most *ZmAP2/ERF* genes also exhibited obvious spatial and temporal expression profiles (Fig. 5). For example, some genes such as *ZmERF60* and *ZmERF133* were specifically expressed in embryos, whereas *ZmERF93* exhibited preferential expression in primary roots and leaves. Furthermore, the analysis of co-expression network indicated that a total of 195 genes were divided into six modules according to their associated patterns (Figs. 6 and 7). Some closely-related genes such as *ZmAP2–12*, *ZmAP2–17*, *ZmAP2–18* and *ZmAP2–19*, shared high expression levels in some specific tissues, indicating that these genes might coordinately participate in developmental regulation, and other genes such as *ZmAP2–20*, *ZmAP2–38* and *ZmAP2–39*, also showed the same effect, indicating functional redundancy among these genes. In addition, the AP2/ERF transcription factors have been demonstrated to extensively regulate gene expression, which might be involved in diverse signal transduction pathways such as SA, JA /ET, and ABA-mediated pathways [55]. Moreover, PPI network analysis further indicated that some ZmAP2 proteins such as ZmAP2–11, ZmAP2–15, ZmAP2–20 and ZmAP2–44, might play crucial roles in crosstalk of different signal transduction pathways through interacting with various ZmERF proteins (Fig. 8).

What’s more, to further investigate possible functions of the *ZmAP2/ERF* genes in abiotic stress responses, the expression patterns of twenty-seven selected genes from the maize *ERF* family were profiled in roots, leaves and stems under three different stress conditions (heat, drought and salt) through qRT-PCR analysis. Thus, these results further uncovered that authenticity of these genes as well as their possible functions in response to heat, drought and salt stresses. Interestingly, almost all 27 *ZmAP2/ERF* genes were involved in three stress responses, whereas most of their expression profiles were different (Figs. 9, 10 and 11). In this study, the majority of 27 selected *ZmAP2/ERF* genes were remarkably induced or repressed when subjected to these three stresses. Seven genes (*ZmERF21, ZmERF41, ZmERF57, ZmERF91, ZmERF108, ZmERF135* and *ZmERF137*) were significantly up-regulated after heat stress treatment, indicating that they were correlated with heat stress response (Fig. 9). Among these genes, *ZmERF135* was greatly up-regulated after heat treatment, and the two homologous genes, *OsDREB2A* in rice and *AtDREB2A* in *Arabidopsis* have been revealed previously to exhibit similar expression levels [22]. This further indicated that homologous genes from various species share many similarities in either gene structure or function. Interestingly, the expression level of *ZmERF91* was up-regulated at more than 700-fold greater levels after heat treatment in leaves compared with the control. Moreover, following drought stress treatment, thirteen genes (*ZmERF41, ZmERF49, ZmERF52, ZmERF53, ZmERF57, ZmERF73, ZmERF74, ZmERF77, ZmERF84, ZmERF91, ZmERF123, ZmERF153* and *ZmERF137*) were highly up-regulated after drought stress treatment at one or more time points (Fig. 10). In contrast, only two members (*ZmERF52* and *ZmERF164*) of these 27 selected genes were significantly induced by salt stress treatment in this study. Therefore, most *ZmERF* genes were significantly down-regulated under salt stress condition at all time points (Fig. 11). These results showed that the *ZmERF* genes were essential for maize in response to heat, drought or salt stresses. Moreover, we have also realized that most of selected *ZmERF* genes were extensively involved in various stress responses, indicating a crosstalk among different stress signaling pathways. However, the functions of these putative genes remain to be further investigated.

## Conclusion

In this study, a total of 214 genes encoding ZmAP2/ERF proteins with complete AP2/ERF domain were identified, which were unevenly distributed on ten chromosomes. On the basis of the number of AP2/ERF domain and the similarities of amino acid sequences, the maize AP2/ERF superfamily genes were mainly divided into three distinct families. The exon/intron distribution, conserved AP2/ERF domain, and motif composition were also analyzed and compared. The close phylogenetic relationship among ZmAP2/ERF, OsAP2/ERF and AtAP2/ERF proteins in the same group or subgroup provided insights into their potential functions. These identified *ZmERF* genes by co-expression network analysis could be considered as candidates for a further investigation on their performance in plant growth, development and stress responses. What’s more, the analysis performed by Protein Protein Interaction network between ZmAP2 and ZmERF proteins enabled us to distinguish components involved in the regulatory network associated with ZmAP2 and ZmERF complex. In addition, the gene expression patterns of *ZmERF* genes in different tissues (roots, stems and leaves) under heat, drought and salt stresses were revealed, respectively. This study of the maize AP2/ERF superfamily in organization, structure, evolution and expression profiling facilitates the functional analysis of *ZmAP2/ERF* genes and lays an important foundation for a better comprehension of the molecular mechanism of abiotic stress tolerance in maize.

## Methods

### Identification and analysis of AP2/ERF superfamily genes in maize

The Hidden Markov Model (HMM) profile of the AP2/ERF domain (PF00847) was obtained from Pfam v27.0 database (http://Pfam.sanger.ac.uk/) [56] and was used to screened the Maize Genetics and Genomics Database (MaizeGDB, http://www.maizegdb.org/), and National Centre for Biotechnology Information (NCBI; http://www.ncbi.nlm.nih.gov/). All hits with expected (E) values less than 1.0 were extracted. The maize AP2/ERF proteins were also obtained from the Plant Transcription Factor Database (PlantTFDB, http://planttfdb.cbi.pku.edu.cn/). The conserved AP2/ERF domain (s) in the non-redundant sequences were examined using NCBI-CDD web server (https://www.ncbi.nlm.nih.gov/cdd) and SMART database [57] (http://smart.embl-heidel berg.de/) to examine each putative protein. The basic information about the putative *ZmAP2/ERF* genes containing the number of amino acids, ORF lengths and chromosome locations were acquired from the B73 maize sequence database. Online server PROTPARAM (http://web. expay.org/protparam/) was utilized to analyze the theoretical isoelectric point (pi), molecular weight (MW), and hydrophilic coefficient (Gravy) of all AP2/ERF protein sequences.

### Multiple sequence alignment of amino acid sequences of the AP2/ERF domain in maize

The alignment of the amino acid sequences of the AP2/ERF domains was performed using Clustal-W and then visualized using Easy Sequencing in PostScript 3.0 [58] (http://espript.ibcp.fr) with the multiple sequence alignment results and the PDB entry 3GCC [27].

### Phylogenetic, gene structure and conserved motif analysis of maize AP2/ERF superfamily

Clustal-W software was utilized to perform multiple sequence alignment under its default setting. The *Arabidopsis* and rice AP2/ERF superfamily protein sequences were acquired from PlantTFDB (http://planttfdb.cbi.pku.edu.cn/). MEGA 7.0 program was used to construct the different phylogenetic trees with neighbor-joining method, using the pairwise deletion option and 1000 bootstrap replicates. The exon-intron structures of the predicted *ZmAP2/ERF* superfamily genes were displayed through GSDS (Gene structure display server; http://gsds.cbi.pku.edu.cn/) database. The conserved motifs in maize AP2/ERF protein sequences were analyzed using the motif analysis tool MEME Suite version 4.0.0 [59] (http://meme.sdsc.edu/meme4/cgi-bin/ meme.cgi), with the following parameters: (1) the maximum number of motifs was set to identify 25 motifs; (2) optimum motif width was set to ≥10 and ≤ 200; (3) occurrences of a single motif distributed among the sequences with model: zero or one per sequence (−mod zoops).

### Chromosomal localization, gene duplication and genome synteny analysis

The chromosomal distribution image of *ZmAP2/ERF* genes was accomplished by MG2C (MapGene2Chromosome v2; http://mg2c.iask.in/mg2c_v2.0/) based on their starting and ending positions in maize chromosomes. For synteny analysis, a BLASTP alignment (E < 1e-5, top 5 matches) was perform across the whole genome. Then, the potential *AP2/ERF* homologous gene pairs were analyzed and identified using the software MCScanX [60] and visualized using Circos v0.55 [61].

### Calculation of Ka/Ks values

The Dual Systeny Plotter software (https://github.com/CJ-Chen/TBtools) [62] was used to calculate the synonymous (Ks) and non-synonymous (Ka) substitution rates of the candidate homologous gene pairs. On the basis of a rate of 6.5 × 10^− 9^ substitutions per site per year, divergence time (T) was calculated using the Ks value with the formula: T = Ks/2λ (λ =6.5 × 10^− 9^) Mya [63].

### Analysis of cis-acting elements in the promoters of maize AP2/ERF genes

The 2 kb upstream sequences of each putative *ZmAP2/ERF* gene were downloaded from Online database phytozome v12.1(https://phytozome.jgi.doe.gov/pz/portal.html), and then were analyzed by PLACE database (http://www.dna.affrc.go.jp/PLACE/) [64] to survey the potential stress response and hormone-related cis-regulatory elements in the promoter regions.

### Expression profile analysis of maize AP2/ERF superfamily genes

The published transcriptome data of maize AP2/ERF superfamily genes was obtain from previous study [33] and was used to analyze the expression patterns of *ZmAP2/ERF* genes during different developmental stages. Normalized gene expression values expressed as FPKM (fragments per kilobase of exon per million fragments mapped) were transformed using log2 (FPKM + 1), and then loaded into the Dual Systeny Plotter software (https://github.com/CJ-Chen/TBtools) [62] for further expression analysis.

### Weighted gene co-expression network and protein protein interaction network analyses

Weighted gene co-expression network analysis (WGCNA) was applied to construct the co-expression network among *ZmAP2*, *ZmRAV* and *ZmERF* genes. The co-expression association of all genes was extracted from RNA-seq data in a non-targeted way, and the highly interconnected modules of co-expression genes were detected. The RNA-seq data was obtained from articles published [65].The normalized FPKM(Fragments per thousand base transcripts per million labeled readings) expression values of 32 *ZmAP2*, 4 *ZmRAV* and 158 *ZmERF* genes were used as input data to construct the co-expression network, while they are in different tissues and organs. Pearson correlation coefficient β = 9(soft threshold power) is used to calculate the adjacency matrix. The Protein-Protein Interaction network (PPI network) was carried out using the STRING database (https://string-db.org/) to output the interaction value data between the ZmAP2 and ZmERF proteins. The results of the analysis were visualized with the software of Cytoscape.

### Plant materials and diverse abiotic stress treatments

The maize seeds (*Zea mays L*. inbred line B73) were supplied from National Engineering Laboratory of Crop Stress Resistance Breeding, Anhui Agricultural University, Hefei, China. The maize inbred line B73 was cultured in a growth chamber with a 16-h light/8-h dark cycle at 28 °C from Anhui Normal University, Wuhu, China. These methods were carried out in accordance with relevant guidelines and regulations. We confirm that all experimental protocols were approved by Anhui Agriculture University and Anhui Normal University. The three-week-old seedlings (three-leaf stage) were subjected to three different abiotic stresses. For heat stress treatment (HTP), the maize seedlings were treated in a 42 °C incubator in the dark for heat stress treatment. To impose the drought treatment, we gently pulled the whole maize seedlings out of the soil. For the salt stress treatment, the maize seedling roots were submerged in 200 mM NaCl solution. The treated seedling roots, leaves and stems were sampled at 0, 1, 2, 4, and 8 h after three diverse abiotic stress treatments, respectively and frozen in liquid nitrogen, and finally stored at − 80 °C for RNA extraction.

### Quantitative real-time PCR analysis (qRT-PCR)

Total RNA was isolated using Trizol reagent (Invitrogen, USA), and then subject to DNase I treatment to remove any genomic DNA contamination. About 3 μg of total RNA was used to synthesize the first-strand cDNA using FastKing RT Kit (TIANGEN, China). The PRIMER EXPRESS version 2.0 software (PE Applied Biosystems, USA) was used to design the qPCR Primers under default parameters (Table S7). The qRT-PCR was performed using a BIO-Rad CFX96™ Real-Time PCR system (BIO RAD, USA) under the following setting condition: 95 °C 30s for pre-incubation, 40 cycles at 95 °C for 5 s, 55 °C for 20 s, and 72 °C for 30 s. The maize *Actin1* gene was used as internal control for normalization with the following primers: ZmActin1-F (5′-CCTCACCGACCACCTAATG-3′) and ZmActin1-R (5′-CCATCAGGCATCT CGTAGC-3′). 96-well optical reaction plates (BIO RAD, USA) were used to perform PCR reactions. Reaction was constructed using 50 ng of cDNA template, 400 nM of each primer, and iTaq™ Universal SYBR Green Supermix (BIO RAD, USA). Therefore, at least three biological replicates have been carried out to make sure the reliability of quantitative analysis. 2^-ΔΔCT^ method was used to analyze the qRT-PCR data [66]. Unstressed sample was used as the control to calculate the relative expression values under various stress conditions. Data points in qPCR time course were plotted as means ± SD of three biological replicates. Afterwards, the significant difference analysis was accomplished by Student’s *t* test (http://www.physics.csbsju.edu/stats/ t-test_bulk_ form.html), at significance level of *P* < 0.05. By using HemI 1.0 software (http://hemi.biocuckoo.org/), we analyzed the corresponding gene expression patterns in maize seedling roots, stems and leaves under different abiotic stresses, respectively.

## Supplementary Information

**Additional file 1.**

**Additional file 2: Figure S1.** Comparison of the deduced amino acid sequences of AP2/ERF domains from ERF family in maize. **Figure S2.** Phylogenetic relationship and gene structure of *AP2/ERF* genes in maize. **Figure S3.** Comparison of gene structure of representative *AP2/ERF* genes in maize and their orthologs in *Arabidopsis*, rice and sorghum. **Figure S4.** Distribution of conserved motifs within each AP2/ERF clade in maize. **Figure S5.** Distribution of major stress-related *cis*-elements in the promoter sequences of the 214 *ZmAP2/ERF* genes. **Figure S6.** Comparative physical mapping showing the degree of orthologous relationships of *ZmAP2/ERF* genes with (A) rice and (B) sorghum. **Table S1.** Complete list of ERF/AP2 transcription factors identified in the maize genome and their predicted features. **Table S2.** Distribution and annotation of conserved motifs in *AP2/ERF* family genes. **Table S3.** The *Ka/Ks* ratios and estimated divergence time for tandem duplicated *ZmAP2/ERF* genes. **Table S4.** The *Ka/Ks* ratios and estimated divergence time for segmentally duplicated *ZmAP2/ERF* genes. **Table S5.** The *Ka/Ks* ratios and estimated divergence time for orthologous ZmAP2/ERF proteins between maize and rice. **Table S6.** The *Ka/Ks* ratios and estimated divergence time for orthologous ZmAP2/ERF proteins between maize and sorghum. **Table S7.** Primers for qPCR.

## Data Availability

The datasets generated during the current study are available in the GenBank repository, http://www.ncbi.nlm.nih.gov/Genbank and the MaizeGDB, http://www.maizegdb.org and their public access to these databases are open. All data generated or analyzed during this study are included in this article and its supplementary information files.

## Abbreviations

TFs: Transcription factor (s)
qRT-PCR: Quantitative real-time PCR
HT: Heat stress
ROS: Reactive oxygen species
AP2/ERF: APETALA2/Ethylene Responsive Factor
AP2: APETALA2
ABA: Abscisic acid
Gly: Glycine
Arg: Arginine
Trp: Tryptophan
Val: Valine
Glu: Glutamic acid
Pro: Proline
Leu: Leucine
Ala: Alanine
Asp: Aspartic acid
Asn: Asparagine
W: Tryptophan
L: Leucine
G: Glycine
Y: Tyrosine
R: Arginine
I: Isoleucine
V: Valine
Ile: Isoleucine
Try: Tryptophan
Thr: Threonine
Phe: Phenylalanine
GSDS: Gene Structure Display Server
ABRE: ABA-responsive element
AuxRR-core: Auxin-responsive element
ERE: Ethylene-responsive element
MBS: MYB binding site
LTR: Low-temperature responsive element
WUN-motif: Wound-responsive element
MeJA: Methyl Jasmonate
Ka: Nonsynonymous substitutions
Ks: Synonymous substitutions
Mya: Million years old
HTP: Heat stress treatment
CK: Normal condition
CRF: Cytokinin responsive factor
ORF: Open reading frame
PI: Theoretical isoelectric point
MW: Molecular weight
SD: Standard Deviation

## References

[1] Boyer JS (1982). Plant productivity and environment. Science.

[2] Bray EA, Bailey-Serres J, Weretilnyk E. Biochemistry and Molecular Biology of Plants, Responses to abiotic stresses. 2000.

[3] Dimova DK, Stevaux O, Frolov MV, Dyson NJ (2003). Cell cycle-dependent and cell cycle-independent control of transcription by the drosophila E2F/RB pathway. Genes Dev.

[4] Sharma M, Kumar R, Solanke A, Sharma R, Tyagi A, Sharma A (2010). Identification, phylogeny, and transcript profiling of ERF family genes during development and abiotic stress treatments in tomato. Mol Gen Genomics.

[5] Susan RW (2005). Homing into the origin of the AP2 DNA binding domain. Trends Plant Sci.

[6] Mizoi J, Shinozaki K, Yamaguchi-Shinozaki K (1819). AP2/ERF family transcription factors in plant abiotic stress responses. Biochimica et Biophysica Acta (BBA) - Gene Regul Mechan.

[7] Riechmann JL, Heard J, Martin G, Reuber L, Jiang C-Z, Keddie J (2000). Arabidopsis transcription factors: genome-wide comparative analysis among eukaryotes. Science.

[8] Kagaya Y, Ohmiya K, Hattori T (1999). RAV1, a novel DNA-binding protein, binds to bipartite recognition sequence through two distinct DNA-binding domains uniquely found in higher plants. Nucleic Acids Res.

[9] Nakano T, Suzuki K, Fujimura T, Shinshi H (2006). Genome-wide analysis of the ERF gene family in Arabidopsis and rice. Plant Physiol.

[10] Riechmann JL, Meyerowitz EM (1998). The AP2/EREBP family of plant transcription factors. Biol Chem.

[11] Jofuku KD, Boer BG, Den Montagu M, Van Okamuro JK (1994). Control of Arabidopsis flower and seed development by the homeotic gene APETALA2. Plant Cell.

[12] Elliott RC, Betzner AS, Huttner E, Oakes MP, Tucker WQ, Gerentes D (1996). AINTEGUMENTA, an APETALA2-like gene of Arabidopsis with pleiotropic roles in ovule development and floral organ growth. Plant Cell.

[13] Klucher KM, Chow H, Reiser L, Fischer RL (1996). The AINTEGUMENTA gene of Arabidopsis required for ovule and female gametophyte development is related to the floral homeotic gene APETALA2. Plant Cell.

[14] Mizukami Y (2001). A matter of size: developmental control of organ size in plants. Curr Opin Plant Biol.

[15] Mizukami Y, Fischer RL (2000). Plant organ size control: AINTEGUMENTA regulates growth and cell numbers during organogenesis. Proc Natl Acad Sci U S A.

[16] Alonso JM, Stepanova AN, Leisse TJ, Kim CJ, Chen H, Shinn P (2003). Genome-wide insertional mutagenesis of Arabidopsis thaliana. Science.

[17] Kee Hoon S, Sung Chul L, Ho Won J, Jeum Kyu H, Byung Kook H (2006). Expression and functional roles of the pepper pathogen-induced transcription factor RAV1 in bacterial disease resistance, and drought and salt stress tolerance. Plant Mol Biol.

[18] Sakuma Y, Liu Q, Dubouzet JG, Abe H, Shinozaki K, Yamaguchi-Shinozaki K (2002). DNA-binding specificity of the ERF/AP2 domain of Arabidopsis DREBs, transcription factors involved in dehydration- and cold-inducible gene expression. Biochem Biophys Res Commun.

[19] Ohme-Takagi M, Shinshi H (1995). Ethylene-inducible DNA binding proteins that interact with an ethylene-responsive element. Plant Cell.

[20] Jiang C, Iu B, Singh J (1996). Requirement of a CCGAC cis-acting element for cold induction of the BN115 gene from winter Brassica napus. Plant Mol Biol.

[21] Yamaguchi-Shinozaki K, Shinozaki K (1994). A novel cis-acting element in an Arabidopsis gene is involved in responsiveness to drought, low-temperature, or high-salt stress. Plant Cell.

[22] Sakuma Y (2006). Dual function of an Arabidopsis transcription factor DREB2A in water-stress-responsive and heat-stress-responsive gene expression. Proc Natl Acad Sci U S A.

[23] Qin F, Kakimoto M, Sakuma Y, Maruyama K, Osakabe Y, Tran L (2007). Regulation and functional analysis of ZmDREB2A in response to drought and heat stresses in Zea mays L. Plant J.

[24] Li D, Zhang Y, Hu X, Shen X, Ma L, Su Z (2011). Transcriptional profiling of Medicago truncatula under salt stress identified a novel CBF transcription factor MtCBF4 that plays an important role in abiotic stress responses. BMC Plant Biol.

[25] Hong E, Lim CW, Han SW, Lee SC (2017). Functional analysis of the pepper ethylene-responsive transcription factor, CaAIEF1, in enhanced ABA sensitivity and drought tolerance. Front Plant Sci.

[26] Cao ZF, Li J, Chen F, Li YQ, Zhou HM, Liu Q (2001). Effect of two conserved amino acid residues on DREB1A function. Biochemistry.

[27] Allen MD, Yamasaki K, Ohme-Takagi M, Tateno M, Suzuki M (1998). A novel mode of DNA recognition by a beta-sheet revealed by the solution structure of the GCC-box binding domain in complex with DNA. EMBO J.

[28] Tiwari S, Belachew A, Ma S, Young M, Ade J, Shen Y (2012). The EDLL motif: a potent plant transcriptional activation domain from AP2/ERF transcription factors. Plant J.

[29] Liu L, White MJ, MacRae TH (1999). Transcription factors and their genes in higher plants functional domains, evolution and regulation. Eur J Biochem.

[30] Song X, Li Y, Hou X (2013). Genome-wide analysis of the AP2/ERF transcription factor superfamily in Chinese cabbage (Brassica rapa ssp. pekinensis). BMC Genomics.

[31] Liu M, Sun W, Ma Z, Zheng T, Huang L, Wu Q (2019). Genome-wide investigation of the AP2/ERF gene family in tartary buckwheat (Fagopyum Tataricum). BMC Plant Biol.

[32] Dubouzet JG, Sakuma Y, Ito Y, Kasuga M, Dubouzet EG, Miura S (2003). OsDREB genes in rice, Oryza sativa L., encode transcription activators that function in drought-, high-salt- and cold-responsive gene expression. Plant J.

[33] Stelpflug SC, Sekhon RS, Vaillancourt B, Hirsch CN, Buell CR, de Leon N, Kaeppler SM. An Expanded Maize Gene Expression Atlas based on RNA Sequencing and its Use to Explore Root Development. Plant Genome. 2016;9(1). 10.3835/plantgenome2015.04.0025. 10.3835/plantgenome2015.04.0025 27898762

[34] Zhu YN, Shi DQ, Ruan MB, Zhang LL, Meng ZH, Liu J (2013). Transcriptome analysis reveals crosstalk of responsive genes to multiple abiotic stresses in cotton (Gossypium hirsutum L.). PLoS One.

[35] Jiang F, Guo M, Yang F, Duncan K, Jackson D, Rafalski A (2012). Mutations in an AP2 transcription factor-like gene affect internode length and leaf shape in maize. PLoS One.

[36] Muller M, Munne-Bosch S (2015). Ethylene response factors: a key regulatory hub in hormone and stress signaling. Plant Physiol.

[37] Qiuyun W (2008). Overexpression of a rice OsDREB1F gene increases salt, drought, and low temperature tolerance in both Arabidopsis and rice. Plant Mol Biol.

[38] Schmidt R, Mieulet D, Hubberten H-M, Obata T, Hoefgen R, Fernie AR (2013). SALT-RESPONSIVE ERF1 regulates reactive oxygen species–dependent signaling during the initial response to salt stress in Rice. Plant Cell.

[39] Wolfe SA, Nekludova L, Pabo CO (2000). DNA recognition by Cys2His2 zinc finger proteins. Annu Rev Biophys Biomol Struct.

[40] Yang CY, Hsu FC, Li JP, Wang NN, Shih MC (2011). The AP2/ERF transcription factor AtERF73/HRE1 modulates ethylene responses during hypoxia in Arabidopsis. Plant Physiol.

[41] Du HW, Huang M, Zhang ZX, Cheng SY (2014). Genome-wide analysis of the AP2/ERF gene family in maize waterlogging stress response. Euphytica.

[42] Hao L, Shi S, Guo H, Li M, Hu P, Wei Y (2020). Genome-wide identification and expression profiles of ERF subfamily transcription factors in *Zea mays*. Peer J.

[43] Liu S, Wang X, Wang H, Xin H, Yang X, Yan J (2013). Genome-wide analysis of ZmDREB genes and their association with natural variation in drought tolerance at seedling stage of Zea mays L. PLoS Genet.

[44] Liu Q, Kasuga M, Sakuma Y, Abe H, Miura S, Yamaguchi-Shinozaki K (1998). Two transcription factors, DREB1 and DREB2, with an EREBP/AP2 DNA binding domain separate two cellular signal transduction pathways in drought- and low-temperature-responsive gene expression, respectively, in Arabidopsis. Plant Cell.

[45] Zhang Z, Li X (2018). Genome-wide identification of AP2/ERF superfamily genes and their expression during fruit ripening of Chinese jujube. Sci Rep.

[46] Zhang CH, Shangguan LF, Ma RJ, Sun X, Tao R, Guo L (2012). Genome-wide analysis of the AP2/ERF superfamily in peach (Prunus persica). Genet Mol Res.

[47] Büttner M, Singh KB (1997). Arabidopsis thaliana ethylene-responsive element binding protein (AtEBP), an ethylene-inducible, GCC box DNA-binding protein interacts with an ocs element binding protein. Proc Natl Acad Sci.

[48] Rashid M, Guangyuan H, Guangxiao Y, Hussain J, Xu Y (2012). AP2/ERF transcription factor in Rice: genome-wide canvas and Syntenic relationships between monocots and Eudicots. Evol Bioinformatics Online.

[49] Meyers BC, Kozik A, Griego A, Kuang H, Michelmore RW (2003). Genome-wide analysis of NBS-LRR-encoding genes in Arabidopsis. Plant Cell.

[50] Yaish MW, Peng M, Rothstein SJ (2009). AtMBD9 modulates Arabidopsis development through the dual epigenetic pathways of DNA methylation and histone acetylation. Plant J.

[51] Swarbreck D, Wilks C, Lamesch P, Berardini TZ, Garcia-Hernandez M, Foerster H (2008). The Arabidopsis information resource (TAIR): gene structure and function annotation. Nucleic Acids Res.

[52] Weber H, Hellmann H (2009). Arabidopsis thaliana BTB/ POZ-MATH proteins interact with members of the ERF/AP2 transcription factor family. FEBS J.

[53] Rashotte AM, Mason MG, Hutchison CE, Ferreira FJ, Schaller GE, Kieber JJ (2006). A subset of Arabidopsis AP2 transcription factors mediates cytokinin responses in concert with a two-component pathway. Proc Natl Acad Sci U S A.

[54] Krizek B (2009). AINTEGUMENTA and AINTEGUMENTA-LIKE6 act redundantly to regulate Arabidopsis floral growth and patterning. Plant Physiol.

[55] Zhang JY, Wang QJ, Guo ZR (2012). Progresses on plant AP2/ERF transcription factors. Yi Chuan.

[56] Finn RD, Bateman A, Clements J, Coggill P, Eberhardt RY, Eddy SR (2014). Pfam: the protein families database. Nucleic Acids Res.

[57] Letunic I, Copley RR, Schmidt S, Ciccarelli FD, Doerks T, Schultz J (2004). SMART 4.0: towards genomic data integration. Nucleic Acids Res.

[58] Gouet P, Robert X, Courcelle E (2003). ESPript/ENDscript: extracting and rendering sequence and 3D information from atomic structures of proteins. Nucleic Acids Res.

[59] Bailey TL, Elkan C (1995). The value of prior knowledge in discovering motifs with MEME. Proc Int Confer Intell Syst Mol Biol.

[60] Haibao T, Bowers JE, Xiyin W, Ray M, Maqsudul A, Paterson AH (2008). Synteny and collinearity in plant genomes. Science.

[61] Krzywinski M, Schein JI (2009). Circos: an information aesthetic for comparative genomics. Genome Res.

[62] Chen C, Chen H, Zhang Y, Thomas HR, Frank MH, He Y, Xia R. TBtools: An Integrative Toolkit Developed for Interactive Analyses of Big Biological Data. Mol Plant. 2020;13(8):1194-202. 10.1016/j.molp.2020.06.009 32585190

[63] Gaut BS, Morton BR, McCaig BC, Clegg MT (1996). Substitution rate comparisons between grasses and palms: synonymous rate differences at the nuclear gene Adh parallel rate differences at the plastid gene rbcL. Proc Natl Acad Sci.

[64] Lescot M, Déhais P, Thijs G, Marchal K, Moreau Y, Van de Peer Y (2002). PlantCARE, a database of plant cis-acting regulatory elements and a portal to tools for in silico analysis of promoter sequences. Nucleic Acids Res.

[65] Walley JW, Sartor RC, Shen Z, Schmitz RJ, Wu KJ, Urich MA (2016). Integration of omic networks in a developmental atlas of maize. Science.

[66] Livak KJ, Schmittgen TD (2001). Analysis of relative gene expression data using real-time quantitative PCR and the 2(−Delta Delta C(T)) method. Methods (San Diego, Calif).

