# Supplementary figures and images for: Genome-wide identification and expression profiling analysis of maize AP2/ERF superfamily genes reveal essential roles in abiotic stress tolerance
Source: BMC Genomics. 2022 Feb 12;23:125. doi: 10.1186/s12864-022-08345-7 (PMC8841118; doi:10.1186/s12864-022-08345-7)

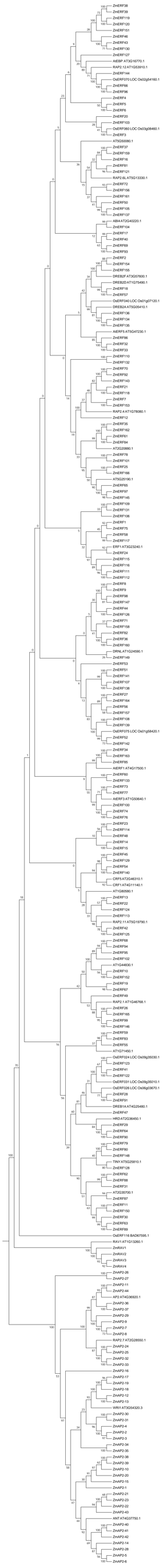

Supplement: Supplementary file 1 — Additional file 1. [file 12864_2022_8345_MOESM1_ESM.pdf]
