# Supplementary material for: Genome-wide identification and expression profiling analysis of maize AP2/ERF superfamily genes reveal essential roles in abiotic stress tolerance
Source: BMC Genomics. 2022 Feb 12;23:125. doi: 10.1186/s12864-022-08345-7 (PMC8841118; doi:10.1186/s12864-022-08345-7)
Supplement: Supplementary file 2 — Additional file 2: Figure S1. Comparison of the deduced amino acid sequences of AP2/ERF domains from ERF family in maize. Figure S2. Phylogenetic relationship and gene structure of AP2/ERF genes in maize. Figure S3. Comparison of gene structure of representative AP2/ERF genes in maize and their orthologs in Arabidopsis, rice and sorghum. Figure S4. Distribution of conserved motifs within each AP2/ERF clade in maize. Figure S5. Distribution of major stress-related cis-elements in the promoter sequences of the 214 ZmAP2/ERF genes. Figure S6. Comparative physical mapping showing the degree of orthologous relationships of ZmAP2/ERF genes with (A) rice and (B) sorghum. Table S1. Complete list of ERF/AP2 transcription factors identified in the maize genome and their predicted features. Table S2. Distribution and annotation of conserved motifs in AP2/ERF family genes. Table S3. The Ka/Ks ratios and estimated divergence time for tandem duplicated ZmAP2/ERF genes. Table S4. The Ka/Ks ratios and estimated divergence time for segmentally duplicated ZmAP2/ERF genes. Table S5. The Ka/Ks ratios and estimated divergence time for orthologous ZmAP2/ERF proteins between maize and rice. Table S6. The Ka/Ks ratios and estimated divergence time for orthologous ZmAP2/ERF proteins between maize and sorghum. Table S7. Primers for qPCR. [file 12864_2022_8345_MOESM2_ESM.docx]

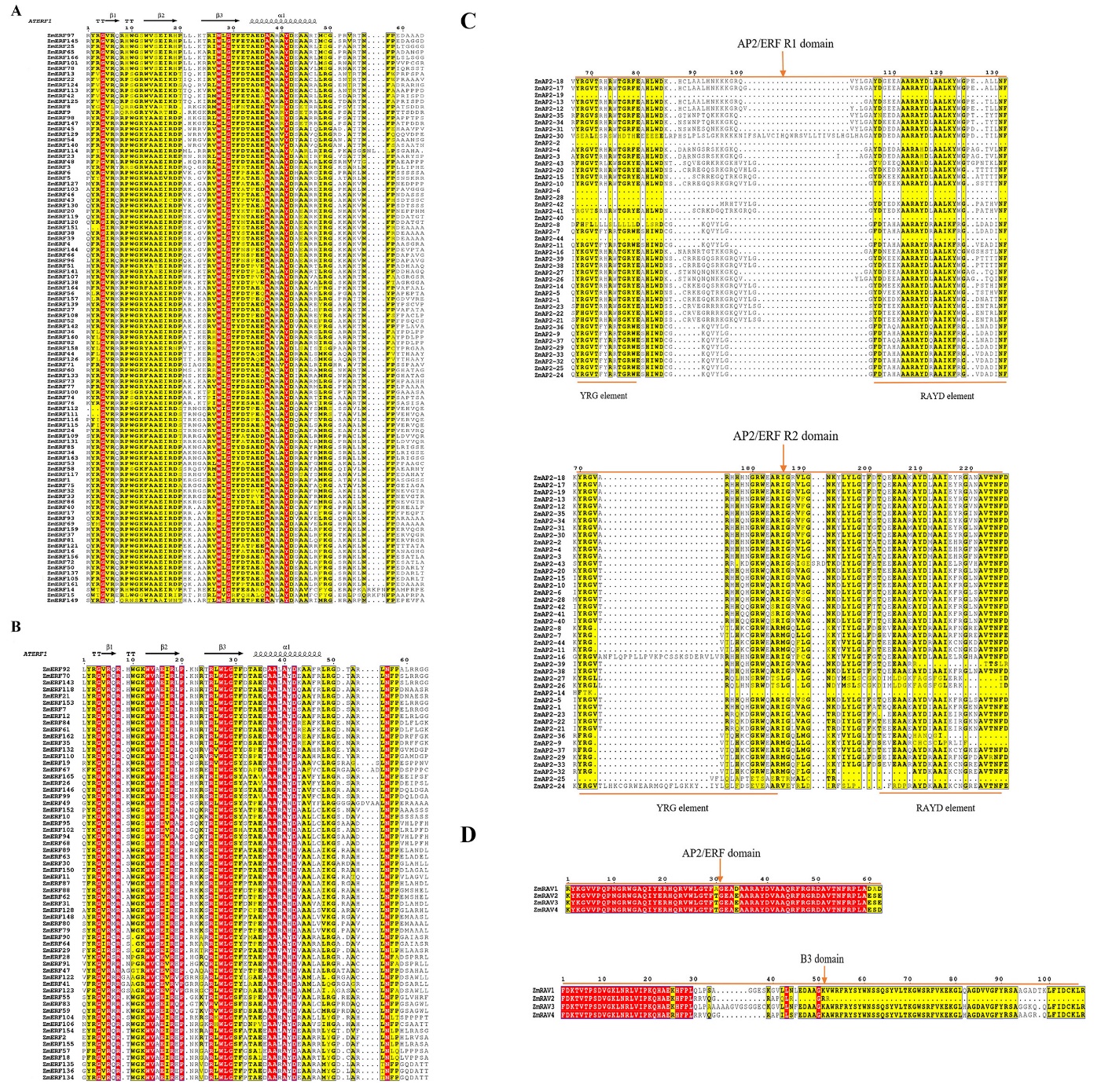


**Fig. S1 Comparison of the deduced amino acid sequences of AP2/ERF domains from ERF family in maize.**

Red and yellow shading indicate identical and conserved amino acid residues, respectively. Secondary structure elements are predicted and presented on top: helices with squiggles, beta strands with arrows, and turns with TT letters. (A) The comparison of the deduced amino acid sequences of the AP2/ERF domains from the ERF subfamily in maize. (B) The comparison of the deduced amino acid sequences of the AP2/ERF domains from the DREB subfamily in maize. (C) the comparison of the deduced amino acid sequences of AP2/ERF domain from the AP2 family in maize. (D) the comparison of the deduced amino acid sequences of AP2/ERF domain and B3 domain from the RAV family in maize.


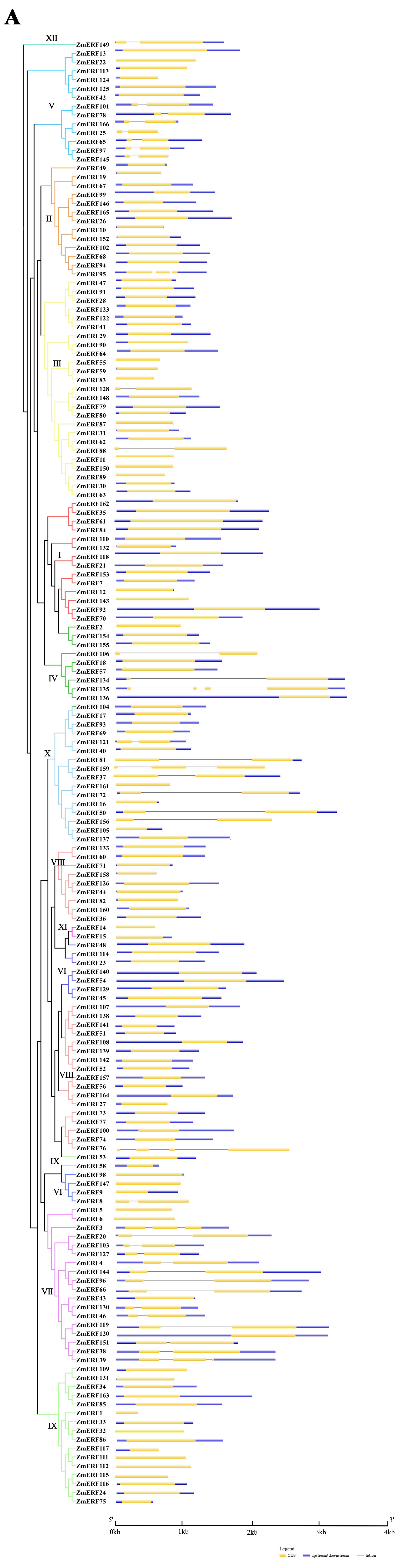

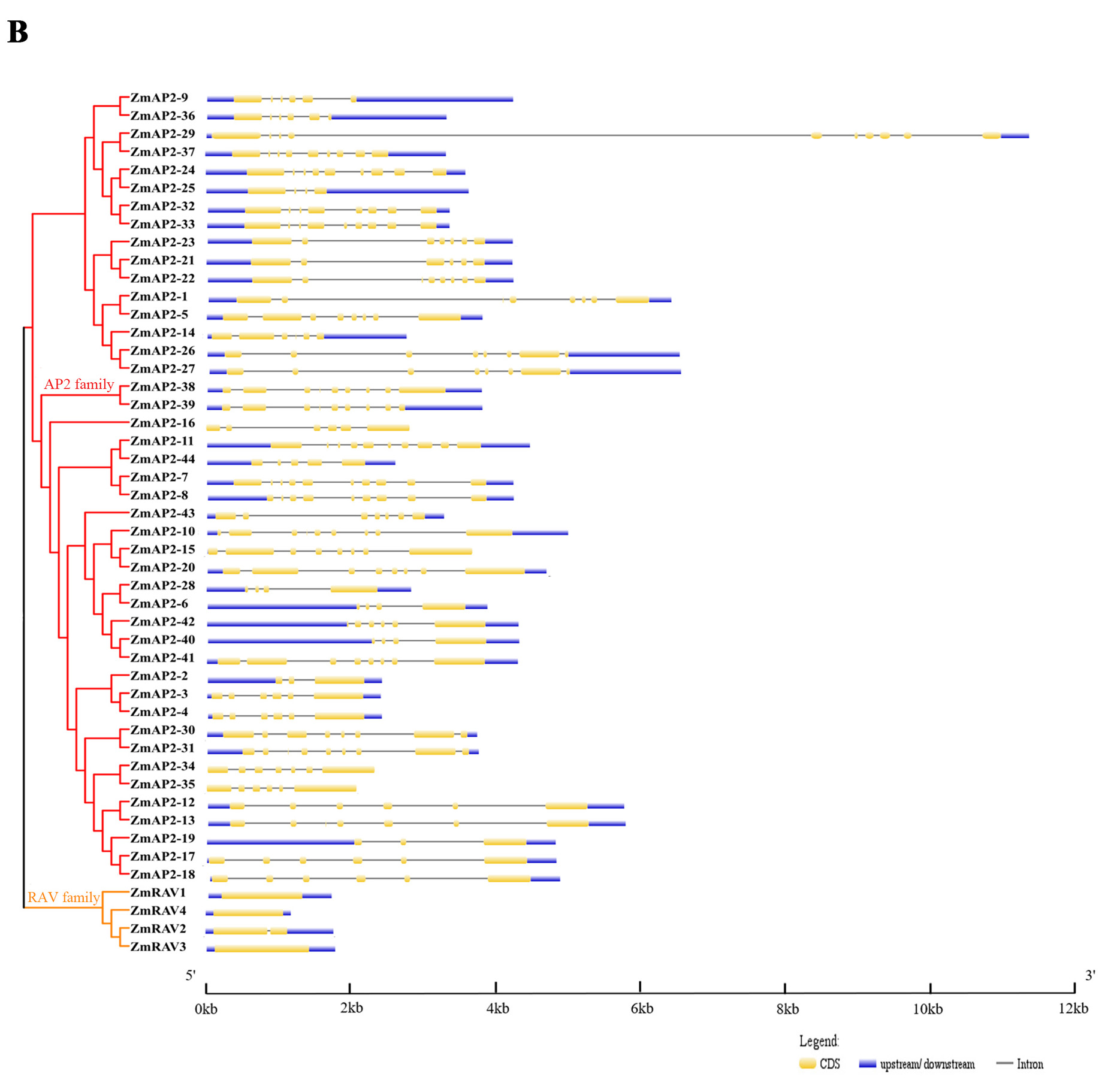


**Fig. S2 Phylogenetic relationship and gene structure of *AP2/ERF* genes in maize**

The unrooted tree was constructed with MEGA 7.0 program using the full-length sequences of the 214 maize AP2/ERF proteins. Exons and introns are indicated by yellow boxes and single lines, respectively. Thick blue lines represent the untranslated regions (UTRs). The length of each *AP2/ERF* gene can be estimated using the scale at the bottom. (A) Gene structure of ERF family in maize. (B) Gene structure of AP2 and RAV families in maize.


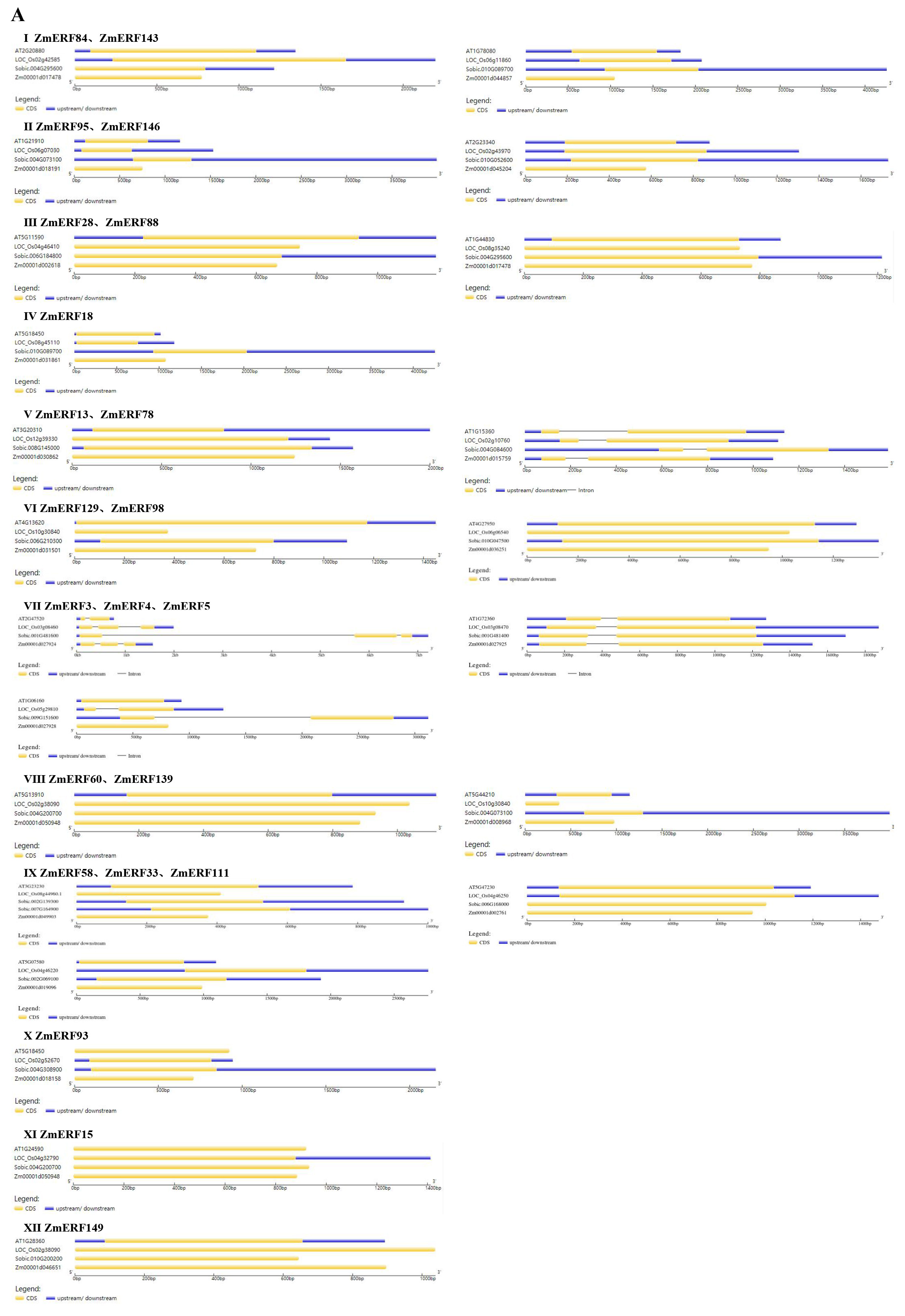

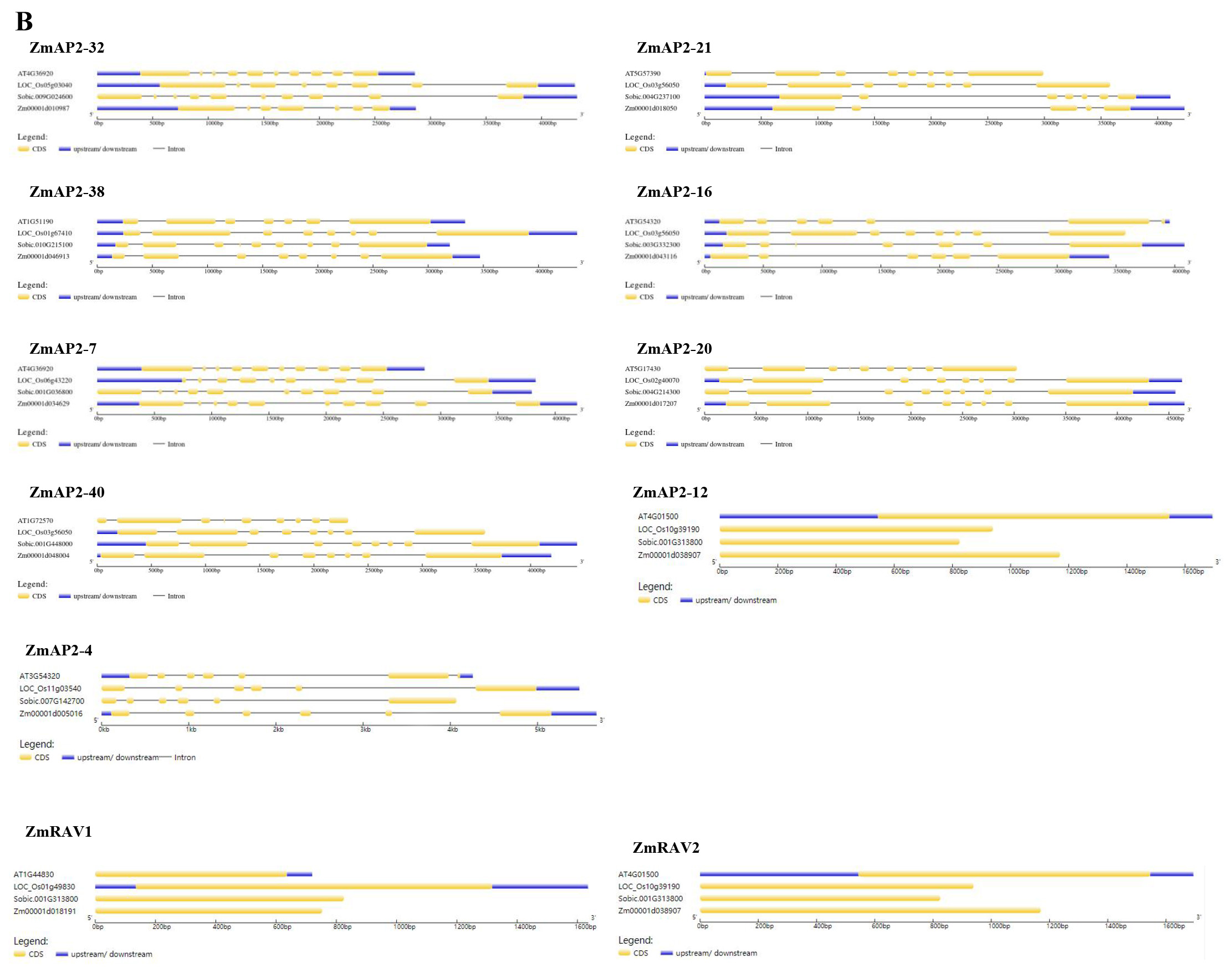


**Fig. S3 Comparison of gene structure of representative *AP2/ERF* genes in maize and their orthologs in *Arabidopsis*, rice and sorghum**

Exons and introns are indicated by yellow boxes and single lines, respectively. Thick blue lines represent the untranslated regions (UTRs). The length of each *AP2/ERF* gene can be estimated using the scale at the bottom. (A) Gene structure of representative *AP2/ERF* genes of the ERF family among different species. (B) Gene structure of representative *AP2/ERF* genes of the AP2 and RAV families among different species.


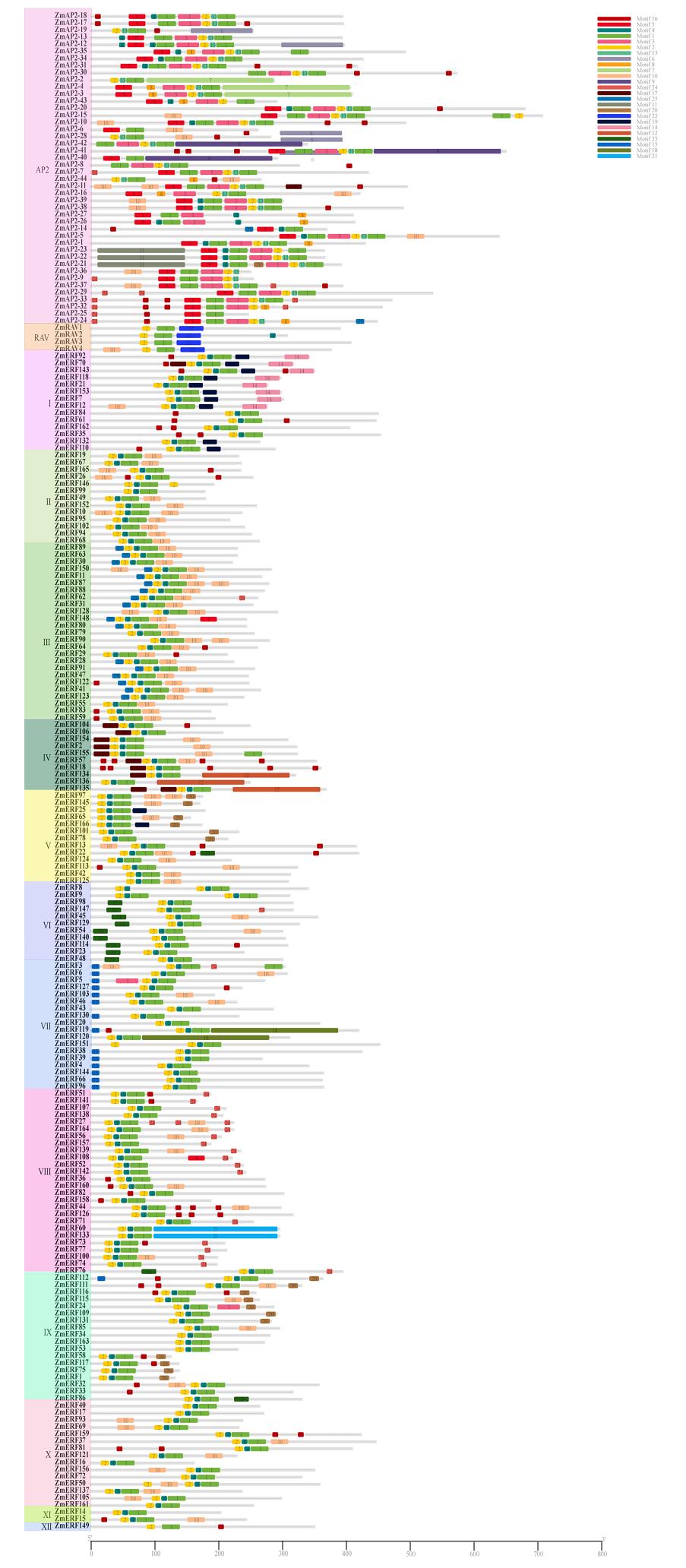


**Fig. S4 Distribution of conserved motifs within each AP2/ERF clade in maize**

Each motif is represented by a number in a colored box. Box length corresponds to motif length and their relative positions are displayed. The amino acid sequences of the conserved motifs are summarized in Table S1.


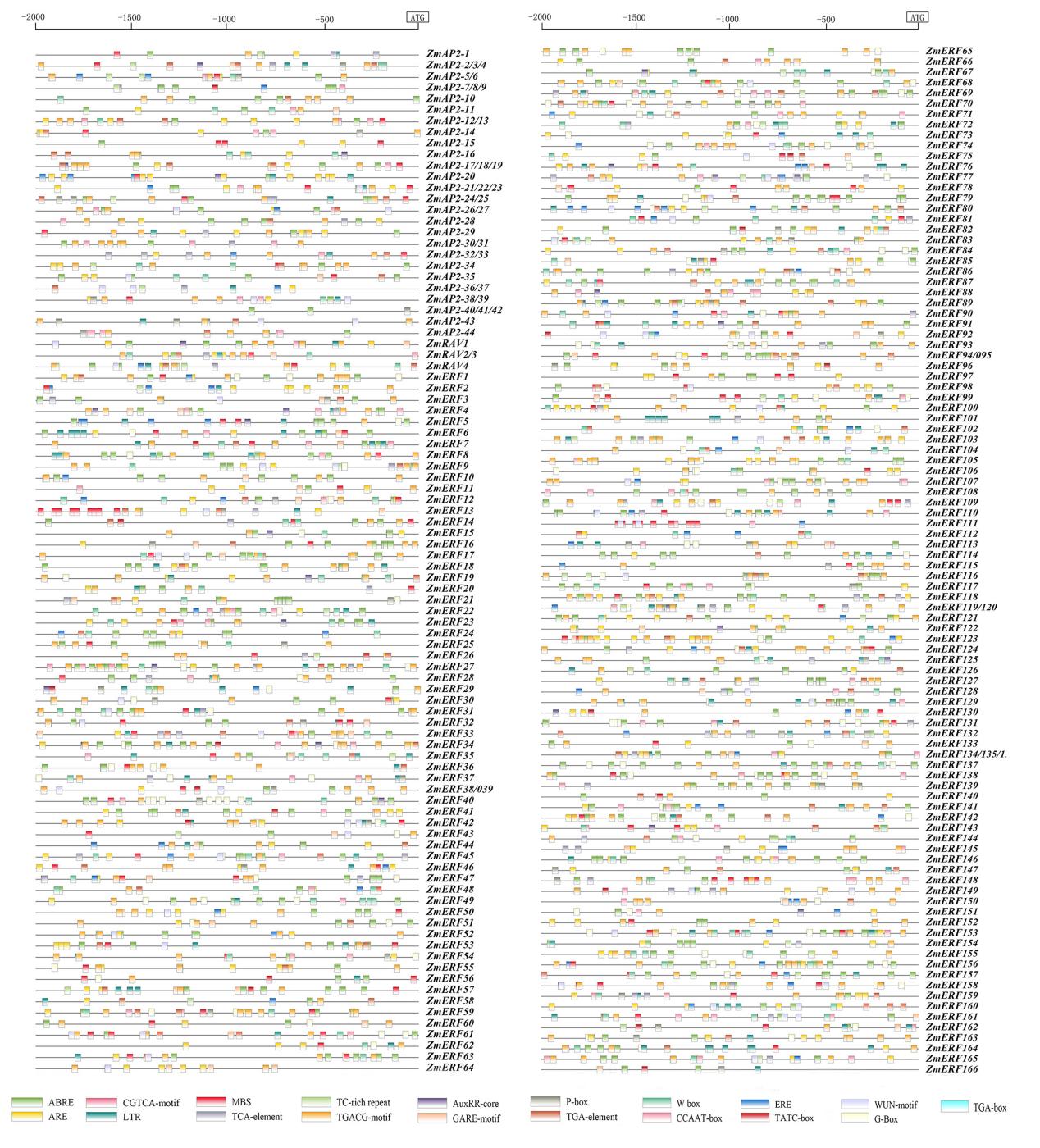


**Fig. S5 Distribution of major stress-related *cis*-elements in the promoter sequences of the 214 *ZmAP2/ERF* genes.**

Putative ABRE, G-Box, CGTCA-motif, TGACG-motif, TCA-element, TGA-box, TGA-element, AuxRR-core, GARE-motif, P-box, TATC-box, ERE, ARE, TC-rich repeats, W box, MBS, LTR, WUN-motif and CCAAT-box core sequences are represented by different colors as indicated in figure key at the bottom. ABRE and G-Box: cis-acting element involved in the abscisic acid responsiveness; CGTCA-motif and TGACG-motif: cis-acting regulatory element involved in the MeJA responsiveness; TCA-element: cis-acting element involved in salicylic acid responsiveness; TGA-box and TGA-element and AuxRR-core: auxin-responsive element; GARE-motif: gibberellin-responsive element; P-box and TATC-box: gibberellin-responsive element; ERE: ethylene-responsive element; ARE: cis-acting regulatory element essential for the anaerobic induction; TC-rich repeats: cis-acting element involved in defense and stress responsiveness; W box: elicitation; wounding and pathogen responsiveness; MBS: MYB binding site involved in drought-inducibility; LTR: cis-acting element involved in low-temperature responsiveness; WUN-motif: wound-responsive element; CCAAT-box: cis-acting element involved in heat stress responsiveness; The 1.5 kb sequences upstream of the initiation codon (ATG) of the *JHDM* genes can be estimated using the scale per 250bp at the above. The 2.0 kb sequences upstream of the initiation codon (ATG) of the *AP2/ERF* genes can be estimated using the scale per 500 bp at the above


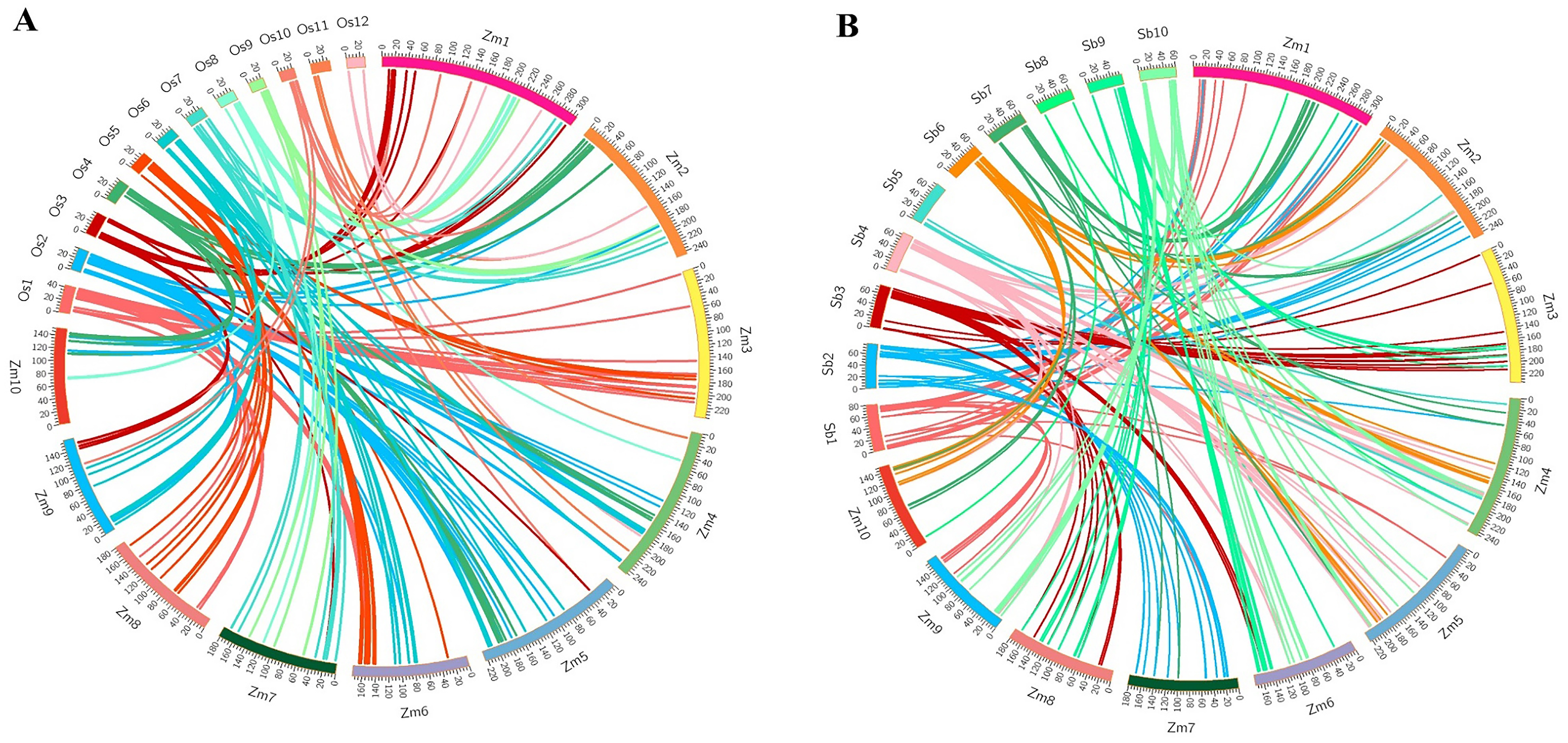


**Fig. S6 Comparative physical mapping showing the degree of orthologous relationships of *ZmAP2/ERF* genes with (A) rice and (B) sorghum**

**Table S1 Complete list of ERF/AP2 transcription factors identified in the maize genome and their predicted features.**

| Serial | | Gene Name | Accession Number | Genome Location | ORF | Protein | | | Chr | Type |
| --- | --- | --- | --- | --- | --- | --- | --- | --- | --- | --- |
| No. | |  | Ensemble transcript | Coordinates (5'-3') | Length | Length | Mol.Wt | PI |  |  |
|  | |  |  |  | (bp) | (a.a.) | (Da) |  |  |  |
| 1 | | ZmAP2-1 | Zm00001d028919_P001 | 51465066-51470982 | 1287 | 428 | 46321.9 | 8.04 | 1 | AP2 |
| 2 | | ZmAP2-2 | Zm00001d032144_P001 | 214378154-214380573 | 1218 | 405 | 44871.7 | 6.12 | 1 | AP2 |
| 3 | | ZmAP2-3 | Zm00001d032144_P002 | 214378154-214380573 | 1227 | 408 | 45113 | 6.12 | 1 | AP2 |
| 4 | | ZmAP2-4 | Zm00001d032144_P003 | 214378154-214380573 | 861 | 286 | 31644.1 | 4.605 | 1 | AP2 |
| 5 | | ZmAP2-5 | Zm00001d034204_P002 | 286726372-286730435 | 1917 | 638 | 66852.12 | 7.046 | 1 | AP2 |
| 6 | | ZmAP2-6 | Zm00001d034204_P001 | 286727282-286730431 | 1503 | 500 | 53,130.03 | 8.255 | 1 | AP2 |
| 7 | | ZmAP2-7 | Zm00001d034629_P003 | 298422859-298427046 | 1215 | 404 | 44,327.41 | 7.31 | 1 | AP2 |
| 8 | | ZmAP2-8 | Zm00001d034629_P001 | 298422859-298427050 | 1302 | 433 | 47604.7 | 6.842 | 1 | AP2 |
| 9 | | ZmAP2-9 | Zm00001d034629_P004 | 298422902-298427037 | 762 | 253 | 28072.7 | 8.785 | 1 | AP2 |
| 10 | | ZmAP2-10 | Zm00001d002025_P003 | 4629747-4634984 | 1479 | 492 | 52568.1 | 6.511 | 2 | AP2 |
| 11 | | ZmAP2-11 | Zm00001d002075_P001 | 5456239-5459958 | 1602 | 533 | 56,053.20 | 8.683 | 2 | AP2 |
| 12 | | ZmAP2-12 | Zm00001d005016_P003 | 153920645-153926320 | 1179 | 392 | 43112.5 | 4.599 | 2 | AP2 |
| 13 | | ZmAP2-13 | Zm00001d005016_P007 | 153920655-153926268 | 1188 | 395 | 43,327.74 | 4.599 | 2 | AP2 |
| 14 | | ZmAP2-14 | Zm00001d007840_P001 | 240624534-240627667 | 1803 | 600 | 63318.89 | 7.35 | 2 | AP2 |
| 15 | | ZmAP2-15 | Zm00001d042492_P001 | 169433935-169438362 | 2121 | 706 | 73216.7 | 6.912 | 3 | AP2 |
| 16 | | ZmAP2-16 | Zm00001d043116_P002 | 189581664-189585104 | 1389 | 462 | 50517.88 | 9.696 | 3 | AP2 |
| 17 | | ZmAP2-17 | Zm00001d052405_P001 | 189190071-189194803 | 762 | 253 | 27842.52 | 4.066 | 4 | AP2 |
| 18 | | ZmAP2-18 | Zm00001d052405_P002 | 189190071-189194808 | 1308 | 435 | 48021.19 | 4.691 | 4 | AP2 |
| 19 | | ZmAP2-19 | Zm00001d052405_P005 | 189190260-189194805 | 762 | 253 | 27,842.52 | 4.066 | 4 | AP2 |
| 20 | | ZmAP2-20 | Zm00001d017207_P001 | 188933076-188937726 | 2031 | 676 | 71413.85 | 6.29 | 5 | AP2 |
| 21 | | ZmAP2-21 | Zm00001d018050_P002 | 213161651-213165819 | 660 | 219 | 23,257.44 | 5.055 | 5 | AP2 |
| 22 | | ZmAP2-22 | Zm00001d018050_P003 | 213161651-213165860 | 1161 | 386 | 42697.78 | 6.935 | 5 | AP2 |
| 23 | | ZmAP2-23 | Zm00001d018050_P006 | 213161673-213165819 | 726 | 241 | 25,700.51 | 5.693 | 5 | AP2 |
| 24 | | ZmAP2-24 | Zm00001d035512_P001 | 30481710-30486777 | 1536 | 511 | 55327.64 | 9.765 | 6 | AP2 |
| 25 | | ZmAP2-25 | Zm00001d035512_P002 | 30481769-30486777 | 1440 | 479 | 51528.96 | 8.504 | 6 | AP2 |
| 26 | | ZmAP2-26 | Zm00001d038087_P004 | 147379164-147385725 | 978 | 325 | 36279.07 | 8.569 | 6 | AP2 |
| 27 | | ZmAP2-27 | Zm00001d038087_P005 | 147379318-147385725 | 948 | 315 | 35098.74 | 5.06 | 6 | AP2 |
| 28 | | ZmAP2-28 | Zm00001d018731_P001 | 3330940-3335717 | 1842 | 613 | 64676.45 | 7.287 | 7 | AP2 |
| 29 | | ZmAP2-29 | Zm00001d019230_P002 | 23054461-23065589 | 1608 | 535 | 57,112.04 | 8.56 | 7 | AP2 |
| 30 | | ZmAP2-30 | Zm00001d020540_P002 | 121545625-121549331 | 1236 | 411 | 44756.83 | 8.769 | 7 | AP2 |
| 31 | | ZmAP2-31 | Zm00001d020540_P001 | 121545625-121549321 | 1182 | 393 | 42854.56 | 9.066 | 7 | AP2 |
| 32 | | ZmAP2-32 | Zm00001d010987_P001 | 136009216-136012084 | 1161 | 386 | 42744.7 | 8.821 | 8 | AP2 |
| 33 | | ZmAP2-33 | Zm00001d010987_P002 | 136009945-136012084 | 1026 | 341 | 37359.49 | 8.298 | 8 | AP2 |
| 34 | | ZmAP2-34 | Zm00001d011134_P001 | 140017045-140019085 | 1263 | 420 | 45,594.05 | 9.212 | 8 | AP2 |
| 35 | | ZmAP2-35 | Zm00001d045378_P001 | 20099934-20101949 | 1476 | 491 | 51510.1 | 7.308 | 9 | AP2 |
| 36 | | ZmAP2-36 | Zm00001d046621_P001 | 95739582-95742689 | 1182 | 393 | 43352.4 | 8.795 | 9 | AP2 |
| 37 | | ZmAP2-37 | Zm00001d046621_P002 | 95739338-95742681 | 750 | 249 | 27613.2 | 10.31 | 9 | AP2 |
| 38 | | ZmAP2-38 | Zm00001d046913_P002 | 107621912-107625433 | 903 | 300 | 32850.2 | 8.938 | 9 | AP2 |
| 39 | | ZmAP2-39 | Zm00001d046913_P001 | 107621912-107625729 | 1458 | 485 | 51,921.89 | 6.19 | 9 | AP2 |
| 40 | | ZmAP2-40 | Zm00001d048004_P001 | 147698120-147702310 | 1977 | 658 | 71189.92 | 7.295 | 9 | AP2 |
| 41 | | ZmAP2-41 | Zm00001d048004_P003 | 147698236-147698112 | 876 | 291 | 31374.1 | 8.308 | 9 | AP2 |
| 42 | | ZmAP2-42 | Zm00001d048004_P002 | 147698236-147702310 | 960 | 319 | 34,276.69 | 7.523 | 9 | AP2 |
| 43 | | ZmAP2-43 | Zm00001d024468_P003 | 72229237-72232453 | 873 | 290 | 31276.8 | 7.174 | 10 | AP2 |
| 44 | | ZmAP2-44 | Zm00001d026448_P001 | 146059085-146062110 | 1242 | 413 | 43497.12 | 9.239 | 10 | AP2 |
| 45 | | ZmRAV1 | Zm00001d043782_P001 | 210069089-210070258 | 1170 | 389 | 40493.6 | 9.919 | 3 | RAV |
| 46 | | ZmRAV2 | Zm00001d038907_P001 | 107621912-107625433 | 1221 | 406 | 42894.4 | 10.32 | 6 | RAV |
| 47 | | ZmRAV3 | Zm00001d038907_P002 | 107621912-107625729 | 921 | 306 | 32931.1 | 11.38 | 6 | RAV |
| 48 | | ZmRAV4 | Zm00001d009468_P001 | 65782410-65783537 | 1128 | 375 | 40278.5 | 10.48 | 8 | RAV |
| 49 | | ZmERF1 | Zm00001d027686_P001 | 11155767-11156159 | 393 | 130 | 13886.3 | 7.961 | 1 | ERF-Ⅸ |
| 50 | | ZmERF2 | Zm00001d027870_P001 | 16005938-16006903 | 966 | 321 | 34078.2 | 6.423 | 1 | ERF-Ⅳ |
| 51 | | ZmERF3 | Zm00001d027924_P001 | 17775779-17777344 | 909 | 302 | 32685.7 | 5.148 | 1 | ERF-Ⅶ |
| 52 | | ZmERF4 | Zm00001d027925_P001 | 17807124-17808643 | 1023 | 340 | 36889.4 | 4.907 | 1 | ERF-Ⅶ |
| 53 | | ZmERF5 | Zm00001d027928_P001 | 17833120-17833935 | 816 | 271 | 28943 | 4.593 | 1 | ERF-Ⅶ |
| 54 | | ZmERF6 | Zm00001d027929_P001 | 17839868-17840788 | 921 | 306 | 33108.2 | 4.496 | 1 | ERF-Ⅶ |
| 55 | | ZmERF7 | Zm00001d028017_P001 | 20410911-20411813 | 903 | 300 | 31910.8 | 7.766 | 1 | DREB-Ⅰ |
| 56 | | ZmERF8 | Zm00001d028066_P001 | 21903337-21911942 | 1020 | 339 | 36429.9 | 7.183 | 1 | ERF-Ⅵ |
| 57 | | ZmERF9 | Zm00001d028070_P001 | 21975168-21977063 | 1812 | 603 | 65,437.47 | 11.16 | 1 | ERF-Ⅵ |
| 58 | | ZmERF10 | Zm00001d028524_P001 | 38080422-38081126 | 705 | 234 | 24242.5 | 4.778 | 1 | DREB-Ⅱ |
| 59 | | ZmERF11 | Zm00001d029884_P001 | 91990667-91991467 | 801 | 266 | 27690.8 | 4.811 | 1 | DREB-Ⅲ |
| 60 | | ZmERF12 | Zm00001d030513_P001 | 140679257-140680084 | 828 | 275 | 29904 | 8.127 | 1 | DREB-Ⅰ |
| 61 | | ZmERF13 | Zm00001d030862_P001 | 164035614-164036858 | 1245 | 414 | 43934.8 | 4.37 | 1 | ERF-Ⅴ |
| 62 | | ZmERF14 | Zm00001d031499_P001 | 192022614-192023222 | 609 | 202 | 21963.9 | 4.834 | 1 | ERF-Ⅺ |
| 63 | | ZmERF15 | Zm00001d031501_P001 | 192063299-192064027 | 729 | 242 | 25489.8 | 7.927 | 1 | ERF-Ⅺ |
| 64 | | ZmERF16 | Zm00001d031673_P001 | 198259709-198260293 | 585 | 194 | 20,631.95 | 10.28 | 1 | ERF-Ⅹ |
| 65 | | ZmERF17 | Zm00001d031796_P001 | 202103550-202104359 | 810 | 269 | 29024.4 | 6.366 | 1 | ERF-Ⅹ |
| 66 | | ZmERF18 | Zm00001d031861_P001 | 204458557-204459636 | 1080 | 359 | 38275.8 | 6.9 | 1 | ERF-Ⅳ |
| 67 | | ZmERF19 | Zm00001d032077_P001 | 211750252-211750944 | 693 | 230 | 24689.3 | 4.532 | 1 | DREB-Ⅱ |
| 68 | | ZmERF20 | Zm00001d032095_P001 | 212364322-212366865 | 1143 | 380 | 42,653.77 | 9.471 | 1 | ERF-Ⅶ |
| 69 | | ZmERF21 | Zm00001d032295_P001 | 220776886-220777674 | 789 | 262 | 28,167.62 | 5.756 | 1 | DREB-Ⅰ |
| 70 | | ZmERF22 | Zm00001d033353_P001 | 260045773-260047029 | 1257 | 418 | 44437.4 | 4.772 | 1 | ERF-Ⅴ |
| 71 | | ZmERF23 | Zm00001d034605_P001 | 297620879-297621595 | 717 | 238 | 25624.3 | 11.43 | 1 | ERF-Ⅵ |
| 72 | | ZmERF24 | Zm00001d034920_P001 | 305637480-305638337 | 858 | 285 | 30759.4 | 9.946 | 1 | ERF-Ⅸ |
| 73 | | ZmERF25 | Zm00001d002011_P001 | 4324615-4325127 | 513 | 170 | 18,214.13 | 4.952 | 2 | ERF-Ⅴ |
| 74 | | ZmERF26 | Zm00001d002079_P001 | 5505054-5505812 | 759 | 252 | 27,243.32 | 10.12 | 2 | DREB-Ⅱ |
| 75 | | ZmERF27 | Zm00001d002364_P001 | 10906553-10907221 | 669 | 222 | 22931.2 | 10.23 | 2 | ERF-Ⅷ |
| 76 | | ZmERF28 | Zm00001d002618_P001 | 16960148-16960816 | 669 | 222 | 24031.7 | 5.159 | 2 | DREB-Ⅲ |
| 77 | | ZmERF29 | Zm00001d002620_P001 | 16974028-16974666 | 639 | 212 | 22205.3 | 7.61 | 2 | DREB-Ⅲ |
| 78 | | ZmERF30 | Zm00001d002744_P001 | 21315391-21316053 | 663 | 220 | 22889.4 | 5.681 | 2 | DREB-Ⅲ |
| 79 | | ZmERF31 | Zm00001d002747_P001 | 21380521-21381279 | 759 | 252 | 26853.8 | 4.583 | 2 | DREB-Ⅲ |
| 80 | | ZmERF32 | Zm00001d002760_P001 | 21850071-21851141 | 1071 | 356 | 37783.3 | 4.584 | 2 | ERF-Ⅸ |
| 81 | | ZmERF33 | Zm00001d002761_P001 | 21893444-21894391 | 948 | 315 | 33823 | 5.019 | 2 | ERF-Ⅸ |
| 82 | | ZmERF34 | Zm00001d002762_P001 | 21962709-21963548 | 840 | 279 | 29881.5 | 5.183 | 2 | ERF-Ⅸ |
| 83 | | ZmERF35 | Zm00001d002867_P001 | 25029642-25031000 | 1359 | 452 | 48127.2 | 9.28 | 2 | DREB-Ⅰ |
| 84 | | ZmERF36 | Zm00001d003871_P001 | 64226755-64227570 | 816 | 271 | 28260.4 | 4.231 | 2 | ERF-Ⅷ |
| 85 | | ZmERF37 | Zm00001d003884_P001 | 65022354-65024176 | 747 | 248 | 26,519.91 | 11.76 | 2 | ERF-Ⅹ |
| 86 | | ZmERF38 | Zm00001d005798_P001 | 189023745-189025630 | 1272 | 423 | 44525.3 | 4.57 | 2 | ERF-Ⅶ |
| 87 | | ZmERF39 | Zm00001d005798_P002 | 189023745-189025631 | 804 | 267 | 28503.1 | 9.919 | 2 | ERF-Ⅶ |
| 88 | | ZmERF40 | Zm00001d005892_P001 | 191897839-191898630 | 792 | 263 | 27923.3 | 6.281 | 2 | ERF-Ⅹ |
| 89 | | ZmERF41 | Zm00001d006169_P001 | 200397718-200398512 | 795 | 264 | 27628.6 | 5.143 | 2 | DREB-Ⅲ |
| 90 | | ZmERF42 | Zm00001d006653_P001 | 213547650-213548585 | 936 | 311 | 33506.4 | 7.976 | 2 | ERF-Ⅴ |
| 91 | | ZmERF43 | Zm00001d007033_P001 | 220493755-220494609 | 855 | 284 | 30655.4 | 9.008 | 2 | ERF-Ⅶ |
| 92 | | ZmERF44 | Zm00001d007119_P001 | 222724965-222725855 | 891 | 296 | 32160 | 7.062 | 2 | ERF-Ⅷ |
| 93 | | ZmERF45 | Zm00001d039324_P001 | 1876337-1877401 | 1065 | 354 | 38189.4 | 4.491 | 3 | ERF-Ⅵ |
| 94 | | ZmERF46 | Zm00001d040651_P001 | 56282555-56283595 | 714 | 237 | 25,480.60 | 7.946 | 3 | ERF-Ⅶ |
| 95 | | ZmERF47 | Zm00001d041981_P001 | 146542859-146543596 | 738 | 245 | 26300.4 | 6.782 | 3 | DREB-Ⅲ |
| 96 | | ZmERF48 | Zm00001d042588_P001 | 172856158-172857057 | 900 | 299 | 31729.8 | 7.327 | 3 | ERF-Ⅵ |
| 97 | | ZmERF49 | Zm00001d042593_P001 | 173057147-173057683 | 537 | 178 | 18669.8 | 9.972 | 3 | DREB-Ⅱ |
| 98 | | ZmERF50 | Zm00001d042717_P001 | 178106747-178109443 | 960 | 319 | 33,086.15 | 6.236 | 3 | ERF-Ⅹ |
| 99 | | ZmERF51 | Zm00001d043204_P001 | 191471796-191472356 | 561 | 186 | 19649.3 | 10.73 | 3 | ERF-Ⅷ |
| 100 | | ZmERF52 | Zm00001d043205_P001 | 191580135-191580848 | 714 | 237 | 24428.5 | 9.004 | 3 | ERF-Ⅷ |
| 101 | | ZmERF53 | Zm00001d043491_P001 | 201681540-201682688 | 690 | 229 | 23282.7 | 9.051 | 3 | ERF-Ⅸ |
| 102 | | ZmERF54 | Zm00001d044004_P001 | 216213660-216214559 | 900 | 299 | 31719 | 4.506 | 3 | ERF-Ⅵ |
| 103 | | ZmERF55 | Zm00001d048991_P001 | 11781874-11782518 | 645 | 214 | 23,087.93 | 4.393 | 4 | DREB-Ⅲ |
| 104 | | ZmERF56 | Zm00001d049364_P001 | 27940728-27941339 | 612 | 203 | 20753.4 | 6.983 | 4 | ERF-Ⅷ |
| 105 | | ZmERF57 | Zm00001d049889_P001 | 50775492-50776550 | 1059 | 352 | 37881.3 | 7.717 | 4 | ERF-Ⅳ |
| 106 | | ZmERF58 | Zm00001d049903_P001 | 51462096-51462470 | 375 | 124 | 13231.6 | 8.917 | 4 | ERF-Ⅸ |
| 107 | | ZmERF59 | Zm00001d050787_P001 | 122810261-122810842 | 582 | 193 | 20659.8 | 4.758 | 4 | DREB-Ⅲ |
| 108 | | ZmERF60 | Zm00001d050948_P001 | 133138782-133139666 | 885 | 294 | 31336.2 | 8.058 | 4 | ERF-Ⅷ |
| 109 | | ZmERF61 | Zm00001d051239_P001 | 149655139-149656476 | 1338 | 445 | 47986.8 | 9.673 | 4 | DREB-Ⅰ |
| 110 | | ZmERF62 | Zm00001d051350_P001 | 155610012-155610749 | 738 | 245 | 26,364.17 | 4.897 | 4 | DREB-Ⅲ |
| 111 | | ZmERF63 | Zm00001d051355_P001 | 155723238-155723924 | 687 | 228 | 23694.1 | 5.295 | 4 | DREB-Ⅲ |
| 112 | | ZmERF64 | Zm00001d051451_P001 | 159111690-159112469 | 780 | 259 | 27019.7 | 6.294 | 4 | DREB-Ⅲ |
| 113 | | ZmERF65 | Zm00001d052026_P001 | 176839153-176840184 | 465 | 154 | 16837.9 | 7.511 | 4 | ERF-Ⅴ |
| 114 | | ZmERF66 | Zm00001d052087_P001 | 179070181-179072822 | 1086 | 361 | 39,462.75 | 4.443 | 4 | ERF-Ⅶ |
| 115 | | ZmERF67 | Zm00001d052102_P001 | 179564558-179565262 | 705 | 234 | 24662.2 | 5.664 | 4 | DREB-Ⅱ |
| 116 | | ZmERF68 | Zm00001d052152_P001 | 181353441-181354229 | 789 | 262 | 26970.9 | 4.408 | 4 | DREB-Ⅱ |
| 117 | | ZmERF69 | Zm00001d052167_P001 | 181560436-181561128 | 693 | 230 | 23962.7 | 8.354 | 4 | ERF-Ⅹ |
| 118 | | ZmERF70 | Zm00001d052229_P001 | 183995749-183996699 | 951 | 316 | 33955.9 | 7.104 | 4 | DREB-Ⅰ |
| 119 | | ZmERF71 | Zm00001d052365_P001 | 188309258-188309908 | 651 | 216 | 22,874.55 | 6.61 | 4 | ERF-Ⅷ |
| 120 | | ZmERF72 | Zm00001d053195_P002 | 219203452-219206136 | 990 | 329 | 34299.9 | 7.148 | 4 | ERF-Ⅹ |
| 121 | | ZmERF73 | Zm00001d053707_P001 | 239645670-239646296 | 627 | 208 | 21684.3 | 7.984 | 4 | ERF-Ⅷ |
| 122 | | ZmERF74 | Zm00001d053859_P001 | 242396086-242396676 | 591 | 196 | 19753 | 5.295 | 4 | ERF-Ⅷ |
| 123 | | ZmERF75 | Zm00001d014113_P001 | 32981437-32981850 | 414 | 137 | 14095.4 | 7.019 | 5 | ERF-Ⅸ |
| 124 | | ZmERF76 | Zm00001d015305_P001 | 81761119-81763618 | 1182 | 393 | 42578.3 | 9.476 | 5 | ERF-Ⅷ |
| 125 | | ZmERF77 | Zm00001d015639_P001 | 102583546-102584181 | 636 | 211 | 22154.9 | 7.668 | 5 | ERF-Ⅷ |
| 126 | | ZmERF78 | Zm00001d015759_P001 | 116972852-116973938 | 642 | 213 | 23355.2 | 10.02 | 5 | ERF-Ⅴ |
| 127 | | ZmERF79 | Zm00001d016260_P001 | 154200894-154201658 | 765 | 254 | 26432.5 | 4.732 | 5 | DREB-Ⅲ |
| 128 | | ZmERF80 | Zm00001d016262_P001 | 154574992-154575720 | 729 | 242 | 26053.3 | 5.964 | 5 | DREB-Ⅲ |
| 129 | | ZmERF81 | Zm00001d016616_P001 | 170223307-170225790 | 768 | 255 | 26489.61 | 7.473 | 5 | ERF-Ⅹ |
| 130 | | ZmERF82 | Zm00001d016623_P001 | 170440595-170441392 | 798 | 265 | 28,944.31 | 4.341 | 5 | ERF-Ⅷ |
| 131 | | ZmERF83 | Zm00001d016848_P001 | 178257482-178258778 | 582 | 193 | 20,976.65 | 9.124 | 5 | DREB-Ⅲ |
| 132 | | ZmERF84 | Zm00001d017366_P001 | 193684947-193686296 | 1350 | 449 | 48039.8 | 9.48 | 5 | DREB-Ⅰ |
| 133 | | ZmERF85 | Zm00001d017462_P001 | 196589211-196590059 | 849 | 282 | 29,532.08 | 6.257 | 5 | ERF-Ⅸ |
| 134 | | ZmERF86 | Zm00001d017466_P001 | 196743195-196744184 | 990 | 329 | 34500.6 | 6.098 | 5 | ERF-Ⅸ |
| 135 | | ZmERF87 | Zm00001d017477_P001 | 197146884-197147717 | 834 | 277 | 28835 | 5.552 | 5 | DREB-Ⅲ |
| 136 | | ZmERF88 | Zm00001d017478_P001 | 197222832-197223605 | 774 | 257 | 27,318.25 | 4.718 | 5 | DREB-Ⅲ |
| 137 | | ZmERF89 | Zm00001d017480_P001 | 197251323-197252009 | 687 | 228 | 23649.2 | 5.299 | 5 | DREB-Ⅲ |
| 138 | | ZmERF90 | Zm00001d017591_P001 | 200872270-200873106 | 837 | 278 | 28693.7 | 6.233 | 5 | DREB-Ⅲ |
| 139 | | ZmERF91 | Zm00001d017592_P001 | 201022603-201023298 | 696 | 231 | 24,603.16 | 4.65 | 5 | DREB-Ⅲ |
| 140 | | ZmERF92 | Zm00001d018081_P001 | 213912954-213913979 | 1026 | 341 | 36569.7 | 6.605 | 5 | DREB-Ⅰ |
| 141 | | ZmERF93 | Zm00001d018158_P001 | 215716411-215717121 | 711 | 236 | 25015.3 | 8.936 | 5 | ERF-Ⅹ |
| 142 | | ZmERF94 | Zm00001d018191_P001 | 216112775-216113527 | 753 | 250 | 25392.2 | 4.424 | 5 | DREB-Ⅱ |
| 143 | | ZmERF95 | Zm00001d018191_P003 | 216112775-216113527 | 651 | 216 | 22931.1 | 12.57 | 5 | DREB-Ⅱ |
| 144 | | ZmERF96 | Zm00001d018305_P001 | 218213866-218216075 | 849 | 282 | 30,430.35 | 4.006 | 5 | ERF-Ⅶ |
| 145 | | ZmERF97 | Zm00001d035835_P001 | 54257239-54258228 | 522 | 173 | 18802.2 | 6.959 | 6 | ERF-Ⅴ |
| 146 | | ZmERF98 | Zm00001d036251_P001 | 79944763-79945710 | 948 | 315 | 33154.7 | 4.499 | 6 | ERF-Ⅵ |
| 147 | | ZmERF99 | Zm00001d036298_P001 | 82562696-82563229 | 534 | 177 | 18561.9 | 9.674 | 6 | DREB-Ⅱ |
| 148 | | ZmERF100 | Zm00001d036536_P001 | 91341686-91342279 | 594 | 197 | 20603.6 | 6.354 | 6 | ERF-Ⅷ |
| 149 | ZmERF101 | Zm00001d036889_P001 | 105660981-105662401 | 693 | 230 | 23823.9 | 10.47 | 6 | ERF-Ⅴ |  |
| 150 | ZmERF102 | Zm00001d037165_P001 | 114596733-114597452 | 720 | 239 | 24445.8 | 4.425 | 6 | DREB-Ⅱ |  |
| 151 | ZmERF103 | Zm00001d037941_P001 | 142909977-142911255 | 582 | 193 | 19,931.55 | 8.498 | 6 | ERF-Ⅶ |  |
| 152 | ZmERF104 | Zm00001d038001_P001 | 144781755-144782501 | 747 | 248 | 26797.8 | 8.771 | 6 | ERF-Ⅳ |  |
| 153 | ZmERF105 | Zm00001d038320_P001 | 154382390-154383061 | 672 | 223 | 23,327.30 | 7.39 | 6 | ERF-Ⅹ |  |
| 154 | ZmERF106 | Zm00001d038446_P001 | 157407857-157411040 | 840 | 279 | 30,711.66 | 7.236 | 6 | ERF-Ⅳ |  |
| 155 | ZmERF107 | Zm00001d038584_P001 | 160527296-160527928 | 633 | 210 | 21726.6 | 10.92 | 6 | ERF-Ⅷ |  |
| 156 | ZmERF108 | Zm00001d038585_P001 | 160539493-160540155 | 663 | 220 | 22995.7 | 8.023 | 6 | ERF-Ⅷ |  |
| 157 | ZmERF109 | Zm00001d039019_P001 | 168819645-168820517 | 873 | 290 | 31026.5 | 6.943 | 6 | ERF-Ⅸ |  |
| 158 | ZmERF110 | Zm00001d039077_P001 | 169626430-169627293 | 864 | 287 | 31114.9 | 9.513 | 6 | DREB-Ⅰ |  |
| 159 | ZmERF111 | Zm00001d019096_P001 | 16182358-16183347 | 990 | 329 | 35072.3 | 6.332 | 7 | ERF-Ⅸ |  |
| 160 | ZmERF112 | Zm00001d019098_P001 | 16383295-16384383 | 1089 | 362 | 38182.7 | 5.785 | 7 | ERF-Ⅸ |  |
| 161 | ZmERF113 | Zm00001d019116_P001 | 17758679-17759647 | 969 | 322 | 34366.3 | 9.627 | 7 | ERF-Ⅴ |  |
| 162 | ZmERF114 | Zm00001d019216_P001 | 22130022-22130944 | 786 | 261 | 28,558.41 | 8.499 | 7 | ERF-Ⅵ |  |
| 163 | ZmERF115 | Zm00001d019475_P001 | 35728209-35728997 | 789 | 262 | 27056.5 | 10.25 | 7 | ERF-Ⅸ |  |
| 164 | ZmERF116 | Zm00001d019734_P001 | 54535486-54536259 | 774 | 257 | 27607.4 | 6.492 | 7 | ERF-Ⅸ |  |
| 165 | ZmERF117 | Zm00001d019744_P001 | 55350694-55351104 | 411 | 136 | 15183.6 | 8.056 | 7 | ERF-Ⅸ |  |
| 166 | ZmERF118 | Zm00001d020267_P001 | 103377384-103378274 | 891 | 296 | 31450 | 7.413 | 7 | DREB-Ⅰ |  |
| 167 | ZmERF119 | Zm00001d020595_P001 | 124130947-124133914 | 1257 | 418 | 44156.7 | 5.172 | 7 | ERF-Ⅶ |  |
| 168 | ZmERF120 | Zm00001d020595_P002 | 124130955-124133900 | 600 | 199 | 20,211.23 | 4.632 | 7 | ERF-Ⅶ |  |
| 169 | ZmERF121 | Zm00001d021089_P001 | 142530712-142532073 | 684 | 227 | 23914.6 | 9.77 | 7 | ERF-Ⅹ |  |
| 170 | ZmERF122 | Zm00001d021205_P001 | 146027044-146027784 | 741 | 246 | 25948.1 | 6.935 | 7 | DREB-Ⅲ |  |
| 171 | ZmERF123 | Zm00001d021207_P001 | 146034693-146035409 | 717 | 238 | 25345 | 4.808 | 7 | DREB-Ⅲ |  |
| 172 | ZmERF124 | Zm00001d021214_P001 | 146343053-146343709 | 657 | 218 | 22964.5 | 10.42 | 7 | ERF-Ⅴ |  |
| 173 | ZmERF125 | Zm00001d021892_P001 | 165528436-165529362 | 927 | 308 | 33021.4 | 7.095 | 7 | ERF-Ⅴ |  |
| 174 | ZmERF126 | Zm00001d022488_P001 | 178605958-178606905 | 948 | 315 | 33332.4 | 7.076 | 7 | ERF-Ⅷ |  |
| 175 | ZmERF127 | Zm00001d022461_P001 | 178147095-178148306 | 708 | 235 | 25733.6 | 6.289 | 7 | ERF-Ⅶ |  |
| 176 | ZmERF128 | Zm00001d008872_P001 | 23393783-23394550 | 768 | 255 | 26,090.99 | 6.588 | 8 | DREB-Ⅲ |  |
| 177 | ZmERF129 | Zm00001d008968_P001 | 27760205-27761182 | 978 | 325 | 35763.7 | 4.432 | 8 | ERF-Ⅵ |  |
| 178 | ZmERF130 | Zm00001d009103_P001 | 36535940-36537052 | 693 | 230 | 24762.8 | 8.702 | 8 | ERF-Ⅶ |  |
| 179 | ZmERF131 | Zm00001d009573_P001 | 71470305-71471572 | 828 | 275 | 29,098.74 | 8.376 | 8 | ERF-Ⅸ |  |
| 180 | ZmERF132 | Zm00001d009622_P001 | 73601361-73602152 | 792 | 263 | 28486.1 | 10.3 | 8 | DREB-Ⅰ |  |
| 181 | ZmERF133 | Zm00001d050948_P001 | 133138782-133139666 | 885 | 294 | 31336.2 | 8.058 | 8 | ERF-Ⅷ |  |
| 182 | ZmERF134 | Zm00001d010048_P003 | 96823514-96826858 | 963 | 320 | 34704.57 | 4.673 | 8 | ERF-Ⅳ |  |
| 183 | ZmERF135 | Zm00001d010048_P004 | 96823544-96826850 | 1104 | 367 | 39579.1 | 4.83 | 8 | ERF-Ⅳ |  |
| 184 | ZmERF136 | Zm00001d010048_P001 | 96823511-96826849 | 747 | 248 | 26545.2 | 4.045 | 8 | ERF-Ⅳ |  |
| 185 | ZmERF137 | Zm00001d010175_P002 | 103046673-103049001 | 1074 | 357 | 36209.27 | 5.802 | 8 | ERF-Ⅹ |  |
| 186 | ZmERF138 | Zm00001d010676_P001 | 123949136-123949753 | 618 | 205 | 21615.6 | 10.69 | 8 | ERF-Ⅷ |  |
| 187 | ZmERF139 | Zm00001d000339_P001 | 120960110-120961302 | 702 | 233 | 23879.8 | 8.329 | 8 | ERF-Ⅷ |  |
| 188 | ZmERF140 | Zm00001d011499_P001 | 152569157-152570068 | 912 | 303 | 31768.9 | 4.412 | 8 | ERF-Ⅵ |  |
| 189 | ZmERF141 | Zm00001d012584_P001 | 176883525-176884022 | 498 | 165 | 17835.9 | 10.29 | 8 | ERF-Ⅷ |  |
| 190 | ZmERF142 | Zm00001d012585_P001 | 176892933-176893658 | 726 | 241 | 24937.4 | 9.362 | 8 | ERF-Ⅷ |  |
| 191 | ZmERF143 | Zm00001d044857_P001 | 5518177-5519226 | 1050 | 349 | 37128.2 | 6.797 | 9 | DREB-Ⅰ |  |
| 192 | ZmERF144 | Zm00001d045044_P001 | 10942420-10945088 | 1092 | 363 | 39301.9 | 4.969 | 9 | ERF-Ⅶ |  |
| 193 | ZmERF145 | Zm00001d045120_P001 | 13432181-13432923 | 510 | 169 | 18025.2 | 6.234 | 9 | ERF-Ⅴ |  |
| 194 | ZmERF146 | Zm00001d045204_P001 | 15996069-15996644 | 576 | 191 | 19587 | 10.56 | 9 | DREB-Ⅱ |  |
| 195 | ZmERF147 | Zm00001d045262_P001 | 17416763-17417710 | 948 | 315 | 32986.8 | 4.754 | 9 | ERF-Ⅵ |  |
| 196 | ZmERF148 | Zm00001d046292_P001 | 78537231-78537959 | 729 | 242 | 25626.1 | 4.683 | 9 | DREB-Ⅲ |  |
| 197 | ZmERF149 | Zm00001d046651_P001 | 100458973-100459869 | 897 | 298 | 31,575.32 | 8.476 | 9 | ERF-ⅩⅡ |  |
| 198 | ZmERF150 | Zm00001d000179_P001 | 115592012-115592857 | 846 | 281 | 29346.6 | 4.628 | 9 | DREB-Ⅲ |  |
| 199 | ZmERF151 | Zm00001d047339_P001 | 126963041-126964782 | 1356 | 451 | 48586.6 | 5.55 | 9 | ERF-Ⅶ |  |
| 200 | ZmERF152 | Zm00001d047860_P001 | 143638759-143639535 | 777 | 258 | 26660.1 | 4.948 | 9 | DREB-Ⅱ |  |
| 201 | ZmERF153 | Zm00001d048208_P001 | 152517945-152518838 | 894 | 297 | 31697.7 | 7.808 | 9 | DREB-Ⅰ |  |
| 202 | ZmERF154 | Zm00001d048296_P001 | 154056208-154057131 | 924 | 307 | 32716.4 | 5.948 | 9 | ERF-Ⅳ |  |
| 203 | ZmERF155 | Zm00001d048297_P001 | 154094049-154095017 | 969 | 322 | 34300.6 | 7.296 | 9 | ERF-Ⅳ |  |
| 204 | ZmERF156 | Zm00001d023535_P001 | 9153462-9156390 | 1299 | 432 | 43,068.50 | 9.85 | 10 | ERF-Ⅹ |  |
| 205 | ZmERF157 | Zm00001d024324_P001 | 64620725-64621285 | 561 | 186 | 19908.3 | 7.637 | 10 | ERF-Ⅷ |  |
| 206 | ZmERF158 | Zm00001d024436_P001 | 70790076-70790636 | 561 | 186 | 19834 | 7.104 | 10 | ERF-Ⅷ |  |
| 207 | ZmERF159 | Zm00001d025281_P001 | 112504950-112507200 | 429 | 142 | 14,764.37 | 10.55 | 10 | ERF-Ⅹ |  |
| 208 | ZmERF160 | Zm00001d025298_P001 | 113224465-113225283 | 819 | 272 | 28369.6 | 4.348 | 10 | ERF-Ⅷ |  |
| 209 | ZmERF161 | Zm00001d025409_P001 | 117748319-117749080 | 762 | 253 | 26051.9 | 9.867 | 10 | ERF-Ⅹ |  |
| 210 | ZmERF162 | Zm00001d025910_P001 | 133559160-133560374 | 1215 | 404 | 43075.4 | 9.363 | 10 | DREB-Ⅰ |  |
| 211 | ZmERF163 | Zm00001d026191_P001 | 140619551-140620363 | 813 | 270 | 29005.4 | 5.726 | 10 | ERF-Ⅸ |  |
| 212 | ZmERF164 | Zm00001d026271_P001 | 142611393-142612061 | 669 | 222 | 22827.9 | 10.52 | 10 | ERF-Ⅷ |  |
| 213 | ZmERF165 | Zm00001d026447_P001 | 145983350-145983982 | 633 | 210 | 23,045.92 | 10.21 | 10 | DREB-Ⅱ |  |
| 214 | ZmERF166 | Zm00001d026486_P001 | 146894287-146895167 | 519 | 172 | 18380.4 | 6.637 | 10 | ERF-Ⅴ |  |

Table S2 Distribution and annotation of conserved motifs in *AP2/ERF* family genes.

| Motif ID | Length | NSites | Evalue | Motif sequence |
| --- | --- | --- | --- | --- |
| Motif-1 | 29 | 214 | 0 | GTFDTAEEAARAYDAAARRLRGANARLNF |
| Motif-2 | 14 | 214 | 0 | GVRQRPWGKWAAEI |
| Motif-3 | 36 | 36 | 0 | DYEDELEEMKNMSREEFVASLRRKSSGFSRGASKYR |
| Motif-4 | 10 | 203 | 0 | DPRKKARVWL |
| Motif-6 | 98 | 5 | 7.90E-271 | DBSVEESLWSPCMDYELDTMSRSNFGSSINLSEWFTDADFDSDJGCLFDGRSAVDGGSKGGVGVADFSLFEAGDGQLKDVLSDMEEGIQPPTIISVCN |
| Motif-7 | 200 | 3 | 3.60E-238 | HYINHWHRHCHGPCDGSLGAMDVAPNVSLELDLLECPATVGLGLEETTGDDEFHNREDYLGHLFGVQQLPDEMGPPAHQMAPASSALDLVLQSPRFKELMQQVSAAGASETNGGSMRSSPSTSLCSFSPSPLELPSPPLQQPTEFIDGAPPRCSFPDDVQSFFDFKNDNDMSFVYAEVDTFLFGDLGAYAPPMFDFDLYE |
| Motif-8 | 14 | 31 | 7.70E-220 | AARAYDLAALKYWG |
| Motif-9 | 200 | 3 | 6.20E-218 | IARYDVDKIMESSTLLAVEEARKVKAVEAASSAPMTHTHSGGKEQLNATTAEETSSAGWRMVLHGSPHQLEAARCPEAADLQSAIMNNDSHPRPSLHGIAGLDIECAVHDHHDHLDVPAGSRTTAAGSINFSNSSSQVTSLGNSREGSPERLGLAMMYGKQPSSAVSLAATMSPWTPVAAQTVAHVLKQQPNVVVSHRPV |
| Motif-10 | 28 | 23 | 4.30E-162 | PGLAHSLPRPASAAPKDVQAAAALAAAA |
| Motif-11 | 138 | 3 | 1.90E-159 | LGKQTEEDKAAGESGDEGKIERSQSINLNRVPAVAVEAMSTQGNCETHGAAVPGTKDAGTGKAGQSSGDRQEEKLPKCEQVDYGSEVEGCADDENPGKRAALVTLVGNEYCGDEDERVQVLTIVKKDEHADDIVDRIN |
| Motif-12 | 138 | 3 | 1.60E-160 | CESTTTSNHSDIASTSHKLEASDISSYLKEKCPAGSCGIQDGTPIVADKEVFGPLEPITNLPDGGDGFDIGEMLRMMESDPHNAGGADAGMGQPWYLDELDSSVLESMLQPEPEPEPEPFLMSEEPDMFLAGFESAGF |
| Motif-13 | 10 | 28 | 1.20E-144 | RVAGNKDLYL |
| Motif-14 | 39 | 8 | 1.00E-137 | PPVPEMQKLDFSEAPWDESEAFHLRKYPSLEIDWDSILS |
| Motif-15 | 13 | 14 | 2.20E-76 | MCGGAILAELIPP |
| Motif-16 | 10 | 49 | 2.10E-74 | HQHQHQQQQQ |
| Motif-17 | 26 | 7 | 7.20E-64 | YGPKRASKKGCMKGKGGPDNAQCEYR |
| Motif-18 | 200 | 2 | 2.50E-62 | DDATGTRHRPTTDEIFNNLKNDDNNNNDDLFAMFAFGDNKKKVPAAKPAAAEGGSGSGSFLVPAPAVAVVPGNKRRSSATNTMLSVSDDQRSNSYGSGSSDLVGSWSWDDDAAAAAMTSDYTSSVFAPDNAVLPAASYTQGGAPKRMRSSYGGAPPSLAHDAAMPGFGLDKVSYHHYQALPPYYVGSSNASVGNLGLLQQ |
| Motif-19 | 23 | 10 | 6.80E-52 | GGAHLAGPLHAAVDAKLQAICQS |
| Motif-20 | 16 | 14 | 9.40E-52 | VIELEDLGAEYLEELL |
| Motif-21 | 195 | 2 | 4.00E-51 | HATAGYWPWGAPQPAAVAHPINPFLLHNLIMSSSNHGCRLLNHAGHGHVHSAAPRPPAPAADATSTTIAAPFPVAAHPAVAMDEDVDDWDGVLRSEPADAGLLQDALHDFYPFTRPRAGGGRRGLSAAGTDARAAAALVAPVKPDAFVVPSPFAGVEGDGEYPMMPQGLLEDVIHSPAFVEVVAAPPSVPTRRGR |
| Motif-22 | 39 | 4 | 6.10E-48 | DPDAAVELRFLASRSKAEVVDMLRKHTYGEELAQNRRAF |
| Motif-23 | 24 | 9 | 6.10E-46 | RPRTVRVFCDDADATDSSGDEEEA |
| Motif-24 | 10 | 27 | 2.90E-45 | LPFDLNLPPP |
| Motif-25 | 14 | 15 | 1.1E-44 | GRRKFRETRHPTYR |

**Table S3 The *Ka/Ks* ratios and estimated divergence time for tandem duplicated *ZmAP2/ERF* genes.**

| Paralogous pairs | Ka | Ks | Ka_Ks | Selective type | Duplication date (MY) | Duplicate type |
| --- | --- | --- | --- | --- | --- | --- |
| ZmERF32-ZmERF33 | 0.22096854 | 0.50803244 | 0.43494967 | Purifying | 39.08 | tandem |
| ZmERF87-ZmERF88 | 0.53909871 | 1.17882734 | 0.45731779 | Purifying | 90.68 | tandem |
| ZmERF111-ZmERF112 | 0.09669059 | 0.16761491 | 0.57686148 | Purifying | 12.89 | tandem |
| Average |  |  | 0.48970965 |  | 47.55 |  |

**Table S4 The *Ka/Ks* ratios and estimated divergence time for segmentally duplicated *ZmAP2/ERF* genes.**

| Paralogous pairs | Ka | Ks | Ka_Ks | Selective type | Duplication date (MY) | Duplicate type |
| --- | --- | --- | --- | --- | --- | --- |
| ZmAP2-5-ZmAP2-14 | 0.247446069 | 0.57692694 | 0.42890365 | Purifying | 44.38 | Segmental |
| ZmAP2-5-ZmAP2-28 | 0.198765965 | 0.58383357 | 0.3404497 | Purifying | 44.91 | Segmental |
| ZmAP2-13-ZmAP2-18 | 0.066896684 | 0.26402602 | 0.25337156 | Purifying | 20.31 | Segmental |
| ZmAP2-24-ZmAP2-32 | 0.105232608 | 0.37763952 | 0.27865888 | Purifying | 29.05 | Segmental |
| ZmAP2-44-ZmAP2-11 | 0.049291822 | 0.13974334 | 0.35273109 | Purifying | 10.75 | Segmental |
| ZmRAV1-ZmRAV2 | 0.287969177 | 0.67051357 | 0.42947554 | Purifying | 51.58 | Segmental |
| ZmRAV1-ZmRAV4 | 0.149636664 | 0.50312111 | 0.29741679 | Purifying | 38.70 | Segmental |
| ZmRAV2-ZmRAV4 | 0.190880859 | 0.35880919 | 0.53198431 | Purifying | 27.60 | Segmental |
| ZmERF1-ZmERF75 | 0.197454442 | 0.66689622 | 0.29607972 | Purifying | 51.30 | Segmental |
| ZmERF2-ZmERF154 | 0.05449765 | 0.16161051 | 0.33721601 | Purifying | 12.43 | Segmental |
| ZmERF7-ZmERF153 | 0.071842784 | 0.17751115 | 0.40472264 | Purifying | 13.65 | Segmental |
| ZmERF10-ZmERF152 | 0.096339144 | 0.18281472 | 0.52697696 | Purifying | 14.06 | Segmental |
| ZmERF11-ZmERF150 | 0.079141697 | 0.20063397 | 0.3944581 | Purifying | 15.43 | Segmental |
| ZmERF12-ZmERF153 | 0.451869609 | 0.93324307 | 0.48419283 | Purifying | 71.79 | Segmental |
| ZmERF17-ZmERF40 | 0.364338972 | 0.50750716 | 0.71789917 | Purifying | 39.04 | Segmental |
| ZmERF18-ZmERF57 | 0.10966139 | 0.27906537 | 0.3929595 | Purifying | 21.47 | Segmental |
| ZmERF21-ZmERF118 | 0.277137426 | 0.59693496 | 0.46426738 | Purifying | 45.92 | Segmental |
| ZmERF28-ZmERF91 | 0.229660618 | 0.49199264 | 0.46679686 | Purifying | 37.85 | Segmental |
| ZmERF29-ZmERF64 | 0.314366828 | 0.63762124 | 0.49303067 | Purifying | 49.05 | Segmental |
| ZmERF29-ZmERF90 | 0.273547984 | 0.7459072 | 0.36673193 | Purifying | 57.38 | Segmental |
| ZmERF30-ZmERF62 | 0.465591169 | 0.63998208 | 0.72750657 | Purifying | 49.23 | Segmental |
| ZmERF30-ZmERF88 | 0.445103645 | 0.61292163 | 0.72619993 | Purifying | 47.15 | Segmental |
| ZmERF32-ZmERF85 | 0.750995634 | 1.16918166 | 0.64232588 | Purifying | 89.94 | Segmental |
| ZmERF35-ZmERF61 | 0.378531222 | 0.75918684 | 0.49860087 | Purifying | 58.40 | Segmental |
| ZmERF35-ZmERF84 | 0.3972761 | 0.86744966 | 0.45798174 | Purifying | 66.73 | Segmental |
| ZmERF36-ZmERF82 | 0.289624548 | 0.55947465 | 0.51767233 | Purifying | 43.04 | Segmental |
| ZmERF37-ZmERF81 | 0.706377472 | 1.02597685 | 0.68849261 | Purifying | 78.92 | Segmental |
| ZmERF38-ZmERF119 | 0.199155829 | 0.47953218 | 0.41531275 | Purifying | 36.89 | Segmental |
| ZmERF41-ZmERF122 | 0.1315538 | 0.26475469 | 0.49688941 | Purifying | 20.37 | Segmental |
| ZmERF42-ZmERF125 | 0.05038832 | 0.17750027 | 0.28387743 | Purifying | 13.65 | Segmental |
| ZmERF44-ZmERF126 | 0.091536816 | 0.15715215 | 0.58247256 | Purifying | 12.09 | Segmental |
| ZmERF45-ZmERF129 | 0.195836284 | 0.21271483 | 0.92065175 | Purifying | 16.36 | Segmental |
| ZmERF50-ZmERF105 | 0.449236728 | 0.78414095 | 0.57290303 | Purifying | 60.32 | Segmental |
| ZmERF51-ZmERF107 | 0.430654208 | 0.92709837 | 0.46451835 | Purifying | 71.32 | Segmental |
| ZmERF51-ZmERF141 | 0.093430935 | 0.16675886 | 0.56027568 | Purifying | 12.83 | Segmental |
| ZmERF54-ZmERF140 | 0.07426267 | 0.21436688 | 0.34642791 | Purifying | 16.49 | Segmental |
| ZmERF59-ZmERF83 | 0.114975135 | 0.26202349 | 0.43879705 | Purifying | 20.16 | Segmental |
| ZmERF61-ZmERF84 | 0.099516028 | 0.1970342 | 0.50506981 | Purifying | 15.16 | Segmental |
| ZmERF62-ZmERF87 | 0.532706307 | 0.99967877 | 0.53287748 | Purifying | 76.90 | Segmental |
| ZmERF64-ZmERF90 | 0.088365541 | 0.20243971 | 0.436503 | Purifying | 15.57 | Segmental |
| ZmERF65-ZmERF145 | 0.138484363 | 0.43789428 | 0.31625068 | Purifying | 33.68 | Segmental |
| ZmERF66-ZmERF96 | 0.072375844 | 0.16593758 | 0.43616308 | Purifying | 12.76 | Segmental |
| ZmERF66-ZmERF144 | 0.270382252 | 1.12585616 | 0.24015701 | Purifying | 86.60 | Segmental |
| ZmERF68-ZmERF94 | 0.100772659 | 0.20293618 | 0.49657316 | Purifying | 15.61 | Segmental |
| ZmERF68-ZmERF102 | 0.336332757 | 0.45476151 | 0.73958052 | Purifying | 34.98 | Segmental |
| ZmERF69-ZmERF93 | 0.144110123 | 0.20539071 | 0.70163895 | Purifying | 15.80 | Segmental |
| ZmERF70-ZmERF92 | 0.056135918 | 0.11802072 | 0.47564461 | Purifying | 9.08 | Segmental |
| ZmERF70-ZmERF143 | 0.238664886 | 0.4860746 | 0.49100464 | Purifying | 37.39 | Segmental |
| ZmERF73-ZmERF77 | 0.057221779 | 0.28328176 | 0.20199599 | Purifying | 21.79 | Segmental |
| ZmERF74-ZmERF76 | 0.046506502 | 0.20144969 | 0.23085914 | Purifying | 15.50 | Segmental |
| ZmERF76-ZmERF100 | 0.233621935 | 0.54809867 | 0.42624066 | Purifying | 42.16 | Segmental |
| ZmERF94-ZmERF102 | 0.325216459 | 0.44433142 | 0.73192316 | Purifying | 34.18 | Segmental |
| ZmERF96-ZmERF144 | 0.28690909 | 1.19849219 | 0.23939171 | Purifying | 92.19 | Segmental |
| ZmERF98-ZmERF147 | 0.197719199 | 0.43438835 | 0.45516691 | Purifying | 33.41 | Segmental |
| ZmERF99-ZmERF146 | 0.056604591 | 0.17183529 | 0.32941191 | Purifying | 13.22 | Segmental |
| ZmERF101-ZmERF137 | 0.91651966 | 1.0945926 | 0.83731578 | Purifying | 84.20 | Segmental |
| ZmERF105-ZmERF138 | 0.839537571 | 1.45560997 | 0.57675997 | Purifying | 111.97 | Segmental |
| ZmERF105-ZmERF141 | 0.771050551 | 1.29457591 | 0.59560088 | Purifying | 99.58 | Segmental |
| ZmERF108-ZmERF139 | 0.090530413 | 0.28623632 | 0.31627857 | Purifying | 22.02 | Segmental |
| ZmERF108-ZmERF142 | 0.314087121 | 0.75282276 | 0.41721257 | Purifying | 57.91 | Segmental |
| ZmERF109-ZmERF131 | 0.15383476 | 0.17980362 | 0.85557097 | Purifying | 13.83 | Segmental |
| ZmERF110-ZmERF132 | 0.123187251 | 0.18265952 | 0.67440914 | Purifying | 14.05 | Segmental |
| ZmERF138-ZmERF141 | 0.348987165 | 0.80873027 | 0.4315248 | Purifying | 62.21 | Segmental |
| ZmERF139-ZmERF142 | 0.236572532 | 0.83517438 | 0.28326124 | Purifying | 64.24 | Segmental |
| ZmERF157-ZmERF164 | 0.404998237 | 0.56053386 | 0.72252234 | Purifying | 43.12 | Segmental |
| ZmERF157-ZmERF56 | 0.338437119 | 0.43689651 | 0.77463909 | Purifying | 33.61 | Segmental |
| ZmERF159-ZmERF37 | 0.12344325 | 0.20879788 | 0.59120931 | Purifying | 16.06 | Segmental |
| ZmERF159-ZmERF81 | 0.582876385 | 1.01606736 | 0.5736592 | Purifying | 78.16 | Segmental |
| ZmERF160-ZmERF36 | 0.056175981 | 0.19454646 | 0.28875355 | Purifying | 14.97 | Segmental |
| ZmERF160-ZmERF82 | 0.304436847 | 0.59243717 | 0.51387195 | Purifying | 45.57 | Segmental |
| ZmERF162-ZmERF35 | 0.051709167 | 0.19373908 | 0.26690107 | Purifying | 14.90 | Segmental |
| ZmERF162-ZmERF61 | 0.357089312 | 0.90905037 | 0.39281576 | Purifying | 69.93 | Segmental |
| ZmERF162-ZmERF84 | 0.329363042 | 0.83710257 | 0.39345602 | Purifying | 64.39 | Segmental |
| ZmERF163-ZmERF32 | 0.793242393 | 0.8714203 | 0.91028679 | Purifying | 67.03 | Segmental |
| ZmERF163-ZmERF85 | 0.169847063 | 0.56446638 | 0.30089846 | Purifying | 43.42 | Segmental |
| ZmERF164-ZmERF27 | 0.089375948 | 0.15866018 | 0.56331681 | Purifying | 12.20 | Segmental |
| ZmERF165-ZmERF26 | 0.056679321 | 0.148672 | 0.38123735 | Purifying | 11.44 | Segmental |
| Average |  |  | 0.48372965 |  | 39.76 |  |

**Table S5 The *Ka/Ks* ratios and estimated divergence time for orthologous ZmAP2/ERF proteins between maize and rice.**

| NO. | Paralogous pairs | Ka | Ks | Ka_Ks | Selective type | Duplication date (MY) |
| --- | --- | --- | --- | --- | --- | --- |
| 1 | ZmAP2-1-LOC_Os03g19900 | 0.306476 | 0.64924481 | 0.472049664 | Purifying | 49.94 |
| 2 | ZmAP2-3-LOC_Os08g34360 | 0.309833 | 0.92535672 | 0.334825936 | Purifying | 71.18 |
| 3 | ZmAP2-5-LOC_Os03g56050 | 0.176921 | 0.47761095 | 0.370428792 | Purifying | 36.74 |
| 4 | ZmAP2-5-LOC_Os07g03250 | 0.201914 | 0.65918817 | 0.306306988 | Purifying | 50.71 |
| 5 | ZmAP2-8-LOC_Os03g60430 | 0.155887 | 0.47066567 | 0.331205946 | Purifying | 36.21 |
| 6 | ZmAP2-8-LOC_Os07g13170 | 0.199878 | 0.88016046 | 0.227092913 | Purifying | 67.70 |
| 7 | ZmAP2-10-LOC_Os04g55970 | 0.173462 | 0.69522634 | 0.249504542 | Purifying | 53.48 |
| 8 | ZmAP2-11-LOC_Os04g55560 | 0.116561 | 0.44648075 | 0.261065179 | Purifying | 34.34 |
| 9 | ZmAP2-13-LOC_Os11g03540 | 0.22871 | 0.95278911 | 0.240042559 | Purifying | 73.29 |
| 10 | ZmAP2-13-LOC_Os12g03290 | 0.227288 | 0.96535397 | 0.235444806 | Purifying | 74.26 |
| 11 | ZmAP2-15-LOC_Os01g67410 | 0.156829 | 0.5831256 | 0.268944877 | Purifying | 44.86 |
| 12 | ZmAP2-16-LOC_Os01g59780 | 0.274398 | 0.58877884 | 0.466046158 | Purifying | 45.29 |
| 13 | ZmAP2-18-LOC_Os11g03540 | 0.233704 | 0.91764715 | 0.254677576 | Purifying | 70.59 |
| 14 | ZmAP2-18-LOC_Os12g03290 | 0.232082 | 0.95512415 | 0.242986136 | Purifying | 73.47 |
| 15 | ZmAP2-20-LOC_Os02g40070 | 0.08079 | 0.4470601 | 0.180714608 | Purifying | 34.39 |
| 16 | ZmAP2-20-LOC_Os04g42570 | 0.178278 | 0.68494876 | 0.26027914 | Purifying | 52.69 |
| 17 | ZmAP2-22-LOC_Os02g51300 | 0.400965 | 0.90838231 | 0.441405904 | Purifying | 69.88 |
| 18 | ZmAP2-24-LOC_Os05g03040 | 0.170252 | 0.71032584 | 0.239681241 | Purifying | 54.64 |
| 19 | ZmAP2-26-LOC_Os05g32270 | 0.111817 | 0.54064395 | 0.206822444 | Purifying | 41.59 |
| 20 | ZmAP2-28-LOC_Os07g03250 | 0.159654 | 0.46746144 | 0.341534884 | Purifying | 35.96 |
| 21 | ZmAP2-29-LOC_Os03g60430 | 0.234652 | 1.01055943 | 0.232199661 | Purifying | 77.74 |
| 22 | ZmAP2-29-LOC_Os07g13170 | 0.161174 | 0.65169024 | 0.247316196 | Purifying | 50.13 |
| 23 | ZmAP2-30-LOC_Os08g34360 | 0.337804 | 0.77622696 | 0.435186808 | Purifying | 59.71 |
| 24 | ZmAP2-32-LOC_Os05g03040 | 0.170666 | 0.59622373 | 0.286245085 | Purifying | 45.86 |
| 25 | ZmAP2-35-LOC_Os06g05340 | 0.220045 | 0.57671175 | 0.38155191 | Purifying | 44.36 |
| 26 | ZmAP2-36-LOC_Os06g43220 | 0.091842 | 0.54745808 | 0.167761421 | Purifying | 42.11 |
| 27 | ZmAP2-39-LOC_Os06g44750 | 0.117219 | 0.48049933 | 0.243953219 | Purifying | 36.96 |
| 28 | ZmAP2-40-LOC_Os03g12950 | 0.162592 | 0.82171706 | 0.197868857 | Purifying | 63.21 |
| 29 | ZmAP2-43-LOC_Os08g07440 | 0.328963 | 0.93047272 | 0.353543935 | Purifying | 71.57 |
| 30 | ZmAP2-44-LOC_Os04g55560 | 0.108995 | 0.44780247 | 0.243399165 | Purifying | 34.45 |
| 31 | ZmRAV1-LOC_Os01g49830 | 0.095988 | 0.4270843 | 0.224750823 | Purifying | 32.85 |
| 32 | ZmRAV1-LOC_Os05g47650 | 0.175476 | 0.48568884 | 0.361293491 | Purifying | 37.36 |
| 33 | ZmRAV2-LOC_Os01g49830 | 0.278634 | 0.73051163 | 0.381422782 | Purifying | 56.19 |
| 34 | ZmRAV2-LOC_Os05g47650 | 0.202647 | 0.5554852 | 0.364810175 | Purifying | 42.73 |
| 35 | ZmRAV4-LOC_Os01g49830 | 0.159971 | 0.52998993 | 0.301837979 | Purifying | 40.77 |
| 36 | ZmRAV4-LOC_Os05g47650 | 0.134554 | 0.42407953 | 0.317284556 | Purifying | 32.62 |
| 37 | ZmERF1-LOC_Os10g30840 | 0.255832 | 0.5841889 | 0.437926291 | Purifying | 44.94 |
| 38 | ZmERF1-LOC_Os03g05590 | 0.217161 | 0.55684442 | 0.389984306 | Purifying | 42.83 |
| 39 | ZmERF2-LOC_Os03g07830 | 0.139737 | 0.42637287 | 0.327734109 | Purifying | 32.80 |
| 40 | ZmERF3-LOC_Os03g08460 | 0.400955 | 0.64849147 | 0.618288932 | Purifying | 49.88 |
| 41 | ZmERF4-LOC_Os03g08470 | 0.310246 | 0.46803198 | 0.662873613 | Purifying | 36.00 |
| 42 | ZmERF5-LOC_Os10g25170 | 0.579844 | 0.54537604 | 1.063200602 | Purifying | 41.95 |
| 43 | ZmERF5-LOC_Os03g08490 | 0.387099 | 0.56226416 | 0.688463887 | Purifying | 43.25 |
| 44 | ZmERF7-LOC_Os03g09170 | 0.179387 | 0.55231646 | 0.324789583 | Purifying | 42.49 |
| 45 | ZmERF10-LOC_Os03g15660 | 0.246503 | 0.44999518 | 0.547789252 | Purifying | 34.62 |
| 46 | ZmERF11-LOC_Os10g41130 | 0.242136 | 0.53715864 | 0.450771484 | Purifying | 41.32 |
| 47 | ZmERF12-LOC_Os03g09170 | 0.43921 | 0.76280578 | 0.575781821 | Purifying | 58.68 |
| 48 | ZmERF12-LOC_Os10g22600 | 0.295616 | 0.67450467 | 0.438270675 | Purifying | 51.88 |
| 49 | ZmERF13-LOC_Os12g39330 | 0.312787 | 0.60663493 | 0.515609554 | Purifying | 46.66 |
| 50 | ZmERF17-LOC_Os08g36920 | 0.192878 | 0.47113385 | 0.409391 | Purifying | 36.24 |
| 51 | ZmERF17-LOC_Os09g28440 | 0.35793 | 0.60019435 | 0.596356569 | Purifying | 46.17 |
| 52 | ZmERF18-LOC_Os08g45110 | 0.371826 | 0.55982735 | 0.664179456 | Purifying | 43.06 |
| 53 | ZmERF19-LOC_Os08g35240 | 0.272795 | 0.98673486 | 0.276462412 | Purifying | 75.90 |
| 54 | ZmERF21-LOC_Os08g31580 | 0.244239 | 0.44377614 | 0.550364746 | Purifying | 34.14 |
| 55 | ZmERF21-LOC_Os09g20350 | 0.328214 | 0.56565263 | 0.580239384 | Purifying | 43.51 |
| 56 | ZmERF22-LOC_Os12g39330 | 0.374005 | 0.79811109 | 0.468612543 | Purifying | 61.39 |
| 57 | ZmERF23-LOC_Os03g60120 | 0.260374 | 0.64224545 | 0.405411638 | Purifying | 49.40 |
| 58 | ZmERF23-LOC_Os07g12510 | 0.557573 | 0.95637761 | 0.583005336 | Purifying | 73.57 |
| 59 | ZmERF24-LOC_Os03g64260 | 0.376555 | 0.85156486 | 0.442192202 | Purifying | 65.50 |
| 60 | ZmERF25-LOC_Os04g56150 | 0.228805 | 0.37496696 | 0.610201386 | Purifying | 28.84 |
| 61 | ZmERF26-LOC_Os04g55520 | 0.143274 | 0.4016488 | 0.356714637 | Purifying | 30.90 |
| 62 | ZmERF27-LOC_Os04g52090 | 0.172236 | 0.3406535 | 0.505603647 | Purifying | 26.20 |
| 63 | ZmERF28-LOC_Os02g45450 | 0.229773 | 0.57094902 | 0.402440952 | Purifying | 43.92 |
| 64 | ZmERF28-LOC_Os04g48350 | 0.109546 | 0.32202176 | 0.340183 | Purifying | 24.77 |
| 65 | ZmERF29-LOC_Os10g38000 | 0.399857 | 0.55700604 | 0.717868554 | Purifying | 42.85 |
| 66 | ZmERF29-LOC_Os02g45420 | 0.240347 | 0.70014075 | 0.343283549 | Purifying | 53.86 |
| 67 | ZmERF29-LOC_Os04g48330 | 0.171021 | 0.47508651 | 0.359978689 | Purifying | 36.55 |
| 68 | ZmERF30-LOC_Os02g43940 | 0.422885 | 0.49465751 | 0.854903973 | Purifying | 38.05 |
| 69 | ZmERF30-LOC_Os04g46410 | 0.392767 | 0.71357429 | 0.550421994 | Purifying | 54.89 |
| 70 | ZmERF32-LOC_Os04g46240 | 0.258059 | 0.61386974 | 0.420380338 | Purifying | 47.22 |
| 71 | ZmERF33-LOC_Os02g43820 | 0.356131 | 0.69659402 | 0.511246413 | Purifying | 53.58 |
| 72 | ZmERF33-LOC_Os04g46220 | 0.885649 | 0.87081541 | 1.017033801 | Purifying | 66.99 |
| 73 | ZmERF35-LOC_Os02g42585 | 0.310331 | 0.83265826 | 0.372699622 | Purifying | 64.05 |
| 74 | ZmERF35-LOC_Os04g44670 | 0.192712 | 0.57829985 | 0.333238287 | Purifying | 44.48 |
| 75 | ZmERF36-LOC_Os02g32040 | 0.228918 | 0.48497037 | 0.472024359 | Purifying | 37.31 |
| 76 | ZmERF36-LOC_Os04g32790 | 0.230102 | 0.516269 | 0.445702407 | Purifying | 39.71 |
| 77 | ZmERF37-LOC_Os04g32620 | 0.329658 | 0.50837471 | 0.648455382 | Purifying | 39.11 |
| 78 | ZmERF38-LOC_Os09g26420 | 0.291429 | 1.11331556 | 0.261766856 | Purifying | 85.64 |
| 79 | ZmERF40-LOC_Os08g36920 | 0.313841 | 0.59561333 | 0.52692084 | Purifying | 45.82 |
| 80 | ZmERF40-LOC_Os09g28440 | 0.318076 | 0.54066709 | 0.588303105 | Purifying | 41.59 |
| 81 | ZmERF40-LOC_Os02g52670 | 0.500897 | 0.53162533 | 0.942199831 | Purifying | 40.89 |
| 82 | ZmERF41-LOC_Os08g43200 | 0.268343 | 0.63347452 | 0.42360425 | Purifying | 48.73 |
| 83 | ZmERF41-LOC_Os09g35010 | 0.359348 | 0.66722507 | 0.538570208 | Purifying | 51.33 |
| 84 | ZmERF42-LOC_Os07g38750 | 0.184571 | 0.45616798 | 0.404612806 | Purifying | 35.09 |
| 85 | ZmERF44-LOC_Os07g47330 | 0.158293 | 0.39028488 | 0.405583755 | Purifying | 30.02 |
| 86 | ZmERF45-LOC_Os01g12440 | 0.215389 | 0.45487003 | 0.473518319 | Purifying | 34.99 |
| 87 | ZmERF46-LOC_Os01g21120 | 0.173031 | 0.497377 | 0.347887338 | Purifying | 38.26 |
| 88 | ZmERF47-LOC_Os01g73770 | 0.145175 | 0.55437256 | 0.261872303 | Purifying | 42.64 |
| 89 | ZmERF49-LOC_Os01g66270 | 0.246436 | 0.41473272 | 0.594204169 | Purifying | 31.90 |
| 90 | ZmERF49-LOC_Os05g34730 | 0.345963 | 0.50333831 | 0.687336589 | Purifying | 38.72 |
| 91 | ZmERF50-LOC_Os01g64790 | 0.229631 | 0.52827909 | 0.434676834 | Purifying | 40.64 |
| 92 | ZmERF50-LOC_Os05g36100 | 0.348776 | 0.69491042 | 0.501900074 | Purifying | 53.45 |
| 93 | ZmERF51-LOC_Os05g41760 | 0.327809 | 0.74668505 | 0.439018502 | Purifying | 57.44 |
| 94 | ZmERF52-LOC_Os01g58420 | 0.112221 | 1.19984729 | 0.093529663 | Purifying | 92.30 |
| 95 | ZmERF53-LOC_Os01g54890 | 0.421672 | 0.5475388 | 0.770121861 | Purifying | 42.12 |
| 96 | ZmERF54-LOC_Os01g46870 | 0.140859 | 0.51191179 | 0.275162115 | Purifying | 39.38 |
| 97 | ZmERF55-LOC_Os11g13840 | 0.201758 | 0.44411989 | 0.454287797 | Purifying | 34.16 |
| 98 | ZmERF57-LOC_Os08g45110 | 0.353869 | 0.57118724 | 0.619532806 | Purifying | 43.94 |
| 99 | ZmERF59-LOC_Os02g35240 | 0.142563 | 0.61347768 | 0.232385561 | Purifying | 47.19 |
| 100 | ZmERF60-LOC_Os02g38090 | 0.285907 | 0.50136138 | 0.570260831 | Purifying | 38.57 |
| 101 | ZmERF61-LOC_Os02g42585 | 0.184414 | 0.40360608 | 0.456916337 | Purifying | 31.05 |
| 102 | ZmERF61-LOC_Os04g44670 | 0.299914 | 0.76826511 | 0.390377977 | Purifying | 59.10 |
| 103 | ZmERF62-LOC_Os02g43970 | 0.366893 | 0.76206056 | 0.48144878 | Purifying | 58.62 |
| 104 | ZmERF62-LOC_Os04g46400 | 0.505408 | 1.05644654 | 0.47840362 | Purifying | 81.27 |
| 105 | ZmERF64-LOC_Os02g45420 | 0.195952 | 0.69866865 | 0.280464276 | Purifying | 53.74 |
| 106 | ZmERF64-LOC_Os04g48330 | 0.273992 | 0.59237314 | 0.462532787 | Purifying | 45.57 |
| 107 | ZmERF65-LOC_Os06g08340 | 0.126362 | 0.53505627 | 0.236165621 | Purifying | 41.16 |
| 108 | ZmERF66-LOC_Os02g54160 | 0.14857 | 0.52407388 | 0.283490374 | Purifying | 40.31 |
| 109 | ZmERF66-LOC_Os06g09390 | 0.279627 | 0.93921619 | 0.297723617 | Purifying | 72.25 |
| 110 | ZmERF67-LOC_Os02g54050 | 0.208267 | 0.47710867 | 0.436519038 | Purifying | 36.70 |
| 111 | ZmERF67-LOC_Os06g09717 | 0.409092 | 0.81984285 | 0.498988474 | Purifying | 63.06 |
| 112 | ZmERF68-LOC_Os06g10780 | 0.298677 | 0.45594069 | 0.655078423 | Purifying | 35.07 |
| 113 | ZmERF69-LOC_Os02g52670 | 0.234389 | 0.40206397 | 0.582965064 | Purifying | 30.93 |
| 114 | ZmERF70-LOC_Os02g51670 | 0.118614 | 0.40220048 | 0.294913421 | Purifying | 30.94 |
| 115 | ZmERF70-LOC_Os06g11860 | 0.218017 | 0.57603637 | 0.378478179 | Purifying | 44.31 |
| 116 | ZmERF72-LOC_Os11g06770 | 0.397499 | 0.58000642 | 0.685336199 | Purifying | 44.62 |
| 117 | ZmERF73-LOC_Os02g09650 | 0.142696 | 0.58356023 | 0.244525913 | Purifying | 44.89 |
| 118 | ZmERF74-LOC_Os02g06330 | 0.184492 | 0.3557701 | 0.518571941 | Purifying | 27.37 |
| 119 | ZmERF74-LOC_Os06g47590 | 0.196212 | 0.46516275 | 0.421812818 | Purifying | 35.78 |
| 120 | ZmERF75-LOC_Os10g30840 | 0.202414 | 0.87151035 | 0.232256613 | Purifying | 67.04 |
| 121 | ZmERF75-LOC_Os03g05590 | 0.29401 | 0.84419854 | 0.348270773 | Purifying | 64.94 |
| 122 | ZmERF76-LOC_Os02g06330 | 0.168078 | 0.49008548 | 0.342955827 | Purifying | 37.70 |
| 123 | ZmERF76-LOC_Os06g47590 | 0.218242 | 0.6315203 | 0.34558134 | Purifying | 48.58 |
| 124 | ZmERF77-LOC_Os02g09650 | 0.131412 | 0.52499063 | 0.250313982 | Purifying | 40.38 |
| 125 | ZmERF78-LOC_Os02g10760 | 0.096352 | 0.60496071 | 0.159270232 | Purifying | 46.54 |
| 126 | ZmERF78-LOC_Os06g40150 | 0.139599 | 0.96821297 | 0.144182262 | Purifying | 74.48 |
| 127 | ZmERF79-LOC_Os02g13710 | 0.226202 | 0.54696413 | 0.413558522 | Purifying | 42.07 |
| 128 | ZmERF79-LOC_Os06g36000 | 0.364973 | 0.59730388 | 0.611034658 | Purifying | 45.95 |
| 129 | ZmERF81-LOC_Os04g32620 | 0.481972 | 1.03478356 | 0.465771041 | Purifying | 79.60 |
| 130 | ZmERF82-LOC_Os02g32040 | 0.222729 | 0.45307155 | 0.491598674 | Purifying | 34.85 |
| 131 | ZmERF82-LOC_Os04g32790 | 0.323409 | 0.56140947 | 0.576066823 | Purifying | 43.19 |
| 132 | ZmERF83-LOC_Os02g35240 | 0.244612 | 0.68610138 | 0.356524569 | Purifying | 52.78 |
| 133 | ZmERF84-LOC_Os02g42585 | 0.142869 | 0.39964974 | 0.357484767 | Purifying | 30.74 |
| 134 | ZmERF84-LOC_Os04g44670 | 0.300659 | 0.66198343 | 0.454179139 | Purifying | 50.92 |
| 135 | ZmERF85-LOC_Os04g46220 | 0.212528 | 0.50867605 | 0.417805536 | Purifying | 39.13 |
| 136 | ZmERF85-LOC_Os02g43790 | 0.146504 | 0.36759046 | 0.39855203 | Purifying | 28.28 |
| 137 | ZmERF87-LOC_Os02g43940 | 0.252154 | 0.60694662 | 0.415447086 | Purifying | 46.69 |
| 138 | ZmERF87-LOC_Os04g46400 | 0.332991 | 0.80119187 | 0.41561991 | Purifying | 61.63 |
| 139 | ZmERF89-LOC_Os04g46440 | 0.235065 | 0.52266915 | 0.449739765 | Purifying | 40.21 |
| 140 | ZmERF90-LOC_Os02g45420 | 0.147191 | 0.76213794 | 0.193128684 | Purifying | 58.63 |
| 141 | ZmERF90-LOC_Os04g48330 | 0.369225 | 0.70228312 | 0.525750209 | Purifying | 54.02 |
| 142 | ZmERF91-LOC_Os02g45450 | 0.166308 | 0.54617004 | 0.304497738 | Purifying | 42.01 |
| 143 | ZmERF91-LOC_Os04g48350 | 0.194158 | 0.59910664 | 0.324078782 | Purifying | 46.09 |
| 144 | ZmERF92-LOC_Os02g51670 | 0.130602 | 0.43655678 | 0.299162721 | Purifying | 33.58 |
| 145 | ZmERF92-LOC_Os06g11860 | 0.247852 | 0.62130421 | 0.398922651 | Purifying | 47.79 |
| 146 | ZmERF93-LOC_Os02g52670 | 0.25439 | 0.41318189 | 0.615686063 | Purifying | 31.78 |
| 147 | ZmERF94-LOC_Os06g10780 | 0.291621 | 0.50239112 | 0.580465936 | Purifying | 38.65 |
| 148 | ZmERF96-LOC_Os02g54160 | 0.155601 | 0.53835375 | 0.2890319 | Purifying | 41.41 |
| 149 | ZmERF96-LOC_Os06g09390 | 0.300785 | 0.92039094 | 0.32680118 | Purifying | 70.80 |
| 150 | ZmERF98-LOC_Os06g06540 | 0.490901 | 0.84205964 | 0.582976771 | Purifying | 64.77 |
| 151 | ZmERF99-LOC_Os06g07030 | 0.183112 | 0.31546613 | 0.58045057 | Purifying | 24.27 |
| 152 | ZmERF100-LOC_Os02g06330 | 0.278261 | 0.55392917 | 0.50234014 | Purifying | 42.61 |
| 153 | ZmERF100-LOC_Os06g47590 | 0.127551 | 0.47829625 | 0.266677209 | Purifying | 36.79 |
| 154 | ZmERF101-LOC_Os02g10760 | 0.227805 | 1.08680071 | 0.209610703 | Purifying | 83.60 |
| 155 | ZmERF101-LOC_Os06g40150 | 0.256409 | 0.42184592 | 0.607826609 | Purifying | 32.45 |
| 156 | ZmERF102-LOC_Os06g10780 | 0.263548 | 0.3838054 | 0.686671977 | Purifying | 29.52 |
| 157 | ZmERF104-LOC_Os05g28350 | 0.237532 | 0.61657946 | 0.385240797 | Purifying | 47.43 |
| 158 | ZmERF105-LOC_Os01g64790 | 0.485772 | 0.67851474 | 0.715933713 | Purifying | 52.19 |
| 159 | ZmERF105-LOC_Os05g36100 | 0.328736 | 0.66226867 | 0.496377876 | Purifying | 50.94 |
| 160 | ZmERF106-LOC_Os05g39590 | 0.34448 | 0.6482101 | 0.531432666 | Purifying | 49.86 |
| 161 | ZmERF107-LOC_Os05g41760 | 0.247404 | 0.42429901 | 0.583089047 | Purifying | 32.64 |
| 162 | ZmERF108-LOC_Os01g58420 | 0.210351 | 1.16272557 | 0.180912087 | Purifying | 89.44 |
| 163 | ZmERF108-LOC_Os05g41780 | 0.165886 | 0.40567123 | 0.408917834 | Purifying | 31.21 |
| 164 | ZmERF109-LOC_Os05g49010 | 0.300633 | 0.63944586 | 0.470145724 | Purifying | 49.19 |
| 165 | ZmERF110-LOC_Os05g49700 | 0.136634 | 0.33893222 | 0.403129982 | Purifying | 26.07 |
| 166 | ZmERF113-LOC_Os07g10410 | 0.357537 | 1.11503167 | 0.3206522 | Purifying | 85.77 |
| 167 | ZmERF114-LOC_Os07g12510 | 0.347045 | 0.61662721 | 0.562812312 | Purifying | 47.43 |
| 168 | ZmERF116-LOC_Os09g39850 | 0.320433 | 0.83330249 | 0.384533245 | Purifying | 64.10 |
| 169 | ZmERF117-LOC_Os09g39810 | 0.391772 | 0.71524838 | 0.547742165 | Purifying | 55.02 |
| 170 | ZmERF118-LOC_Os08g31580 | 0.294552 | 0.50758325 | 0.580303554 | Purifying | 39.04 |
| 171 | ZmERF118-LOC_Os09g20350 | 0.125662 | 0.4111748 | 0.305617913 | Purifying | 31.63 |
| 172 | ZmERF122-LOC_Os08g43200 | 0.40011 | 0.65892097 | 0.607220504 | Purifying | 50.69 |
| 173 | ZmERF122-LOC_Os09g35010 | 0.404633 | 0.60085186 | 0.673431552 | Purifying | 46.22 |
| 174 | ZmERF125-LOC_Os07g38750 | 0.248785 | 0.45961101 | 0.541295065 | Purifying | 35.35 |
| 175 | ZmERF126-LOC_Os07g47330 | 0.159505 | 0.44477616 | 0.35861806 | Purifying | 34.21 |
| 176 | ZmERF127-LOC_Os07g47790 | 0.210928 | 0.31557539 | 0.668390854 | Purifying | 24.28 |
| 177 | ZmERF128-LOC_Os01g10370 | 0.324576 | 0.52739938 | 0.615428095 | Purifying | 40.57 |
| 178 | ZmERF129-LOC_Os01g12440 | 0.246591 | 0.51865695 | 0.475441913 | Purifying | 39.90 |
| 179 | ZmERF131-LOC_Os05g49010 | 0.340501 | 0.65271474 | 0.52166898 | Purifying | 50.21 |
| 180 | ZmERF132-LOC_Os05g49700 | 0.177851 | 0.41275161 | 0.430890203 | Purifying | 31.75 |
| 181 | ZmERF137-LOC_Os01g64790 | 0.302397 | 0.62415012 | 0.484493724 | Purifying | 48.01 |
| 182 | ZmERF137-LOC_Os05g36100 | 0.226412 | 0.5334442 | 0.424434492 | Purifying | 41.03 |
| 183 | ZmERF138-LOC_Os05g41760 | 0.185243 | 0.48273823 | 0.383734249 | Purifying | 37.13 |
| 184 | ZmERF139-LOC_Os01g58420 | 0.186154 | 1.12079366 | 0.166090891 | Purifying | 86.21 |
| 185 | ZmERF139-LOC_Os05g41780 | 0.125742 | 0.50820034 | 0.247426799 | Purifying | 39.09 |
| 186 | ZmERF140-LOC_Os01g46870 | 0.146731 | 0.5106295 | 0.287352881 | Purifying | 39.28 |
| 187 | ZmERF141-LOC_Os05g41760 | 0.310777 | 0.63008011 | 0.493233741 | Purifying | 48.47 |
| 188 | ZmERF142-LOC_Os01g58420 | 0.181895 | 1.03014096 | 0.176573364 | Purifying | 79.24 |
| 189 | ZmERF142-LOC_Os05g41780 | 0.218595 | 0.74589923 | 0.293062978 | Purifying | 57.38 |
| 190 | ZmERF143-LOC_Os02g51670 | 0.26961 | 0.56759618 | 0.475003188 | Purifying | 43.66 |
| 191 | ZmERF143-LOC_Os06g11860 | 0.163741 | 0.41044273 | 0.398936317 | Purifying | 31.57 |
| 192 | ZmERF144-LOC_Os02g54160 | 0.246966 | 1.28022845 | 0.192907932 | Purifying | 98.48 |
| 193 | ZmERF144-LOC_Os06g09390 | 0.205346 | 0.76695127 | 0.267743465 | Purifying | 59.00 |
| 194 | ZmERF145-LOC_Os06g08340 | 0.201882 | 0.43619422 | 0.462825036 | Purifying | 33.55 |
| 195 | ZmERF145-LOC_Os02g55380 | 0.229837 | 0.49523736 | 0.46409501 | Purifying | 38.10 |
| 196 | ZmERF146-LOC_Os06g07030 | 0.213087 | 0.43738377 | 0.487186565 | Purifying | 33.64 |
| 197 | ZmERF147-LOC_Os06g06540 | 0.446421 | 0.79070254 | 0.564588053 | Purifying | 60.82 |
| 198 | ZmERF148-LOC_Os02g13710 | 0.307904 | 0.745375 | 0.413086048 | Purifying | 57.34 |
| 199 | ZmERF150-LOC_Os10g41130 | 0.206338 | 0.51196877 | 0.403028496 | Purifying | 39.38 |
| 200 | ZmERF152-LOC_Os03g15660 | 0.213445 | 0.41028422 | 0.520237248 | Purifying | 31.56 |
| 201 | ZmERF153-LOC_Os03g09170 | 0.212057 | 0.45449225 | 0.466581029 | Purifying | 34.96 |
| 202 | ZmERF153-LOC_Os10g22600 | 0.358815 | 0.62514794 | 0.573967822 | Purifying | 48.09 |
| 203 | ZmERF154-LOC_Os03g07830 | 0.164679 | 0.46986675 | 0.350481009 | Purifying | 36.14 |
| 204 | ZmERF158-LOC_Os08g07700 | 0.285063 | 0.43544013 | 0.654654202 | Purifying | 33.50 |
| 205 | ZmERF159-LOC_Os04g32620 | 0.223501 | 0.49575793 | 0.450826957 | Purifying | 38.14 |
| 206 | ZmERF160-LOC_Os02g32040 | 0.284152 | 0.48388795 | 0.587227796 | Purifying | 37.22 |
| 207 | ZmERF160-LOC_Os04g32790 | 0.208908 | 0.54487615 | 0.383404328 | Purifying | 41.91 |
| 208 | ZmERF161-LOC_Os02g34260 | 0.358678 | 0.71434422 | 0.502108248 | Purifying | 54.95 |
| 209 | ZmERF162-LOC_Os02g42585 | 0.288817 | 0.793027 | 0.364195127 | Purifying | 61.00 |
| 210 | ZmERF162-LOC_Os04g44670 | 0.214606 | 0.63921064 | 0.33573549 | Purifying | 49.17 |
| 211 | ZmERF163-LOC_Os04g46220 | 0.160127 | 0.45594282 | 0.351199374 | Purifying | 35.07 |
| 212 | ZmERF163-LOC_Os02g43790 | 0.229278 | 0.57911558 | 0.395911457 | Purifying | 44.55 |
| 213 | ZmERF164-LOC_Os04g52090 | 0.201433 | 0.40605853 | 0.496069531 | Purifying | 31.24 |
| 214 | ZmERF165-LOC_Os04g55520 | 0.148798 | 0.37456864 | 0.397252387 | Purifying | 28.81 |
| 215 | ZmERF115-LOC_Os07g22730 | 0.329489 | 0.5848655 | 0.563358802 | Purifying | 44.99 |
| Average |  |  |  | 0.428242185 |  | 47.08 |

**Table S6 The *Ka/Ks* ratios and estimated divergence time for orthologous ZmAP2/ERF proteins between maize and sorghum.**

| NO. | Paralogous pairs | Ka | Ks | Ka_Ks | Selective type | Duplication date (MY) |
| --- | --- | --- | --- | --- | --- | --- |
| 1 | ZmAP2-1-Sobic.001G392700 | 0.047442284 | 0.119981569 | 0.395413098 | Purifying | 9.23 |
| 2 | ZmAP2-3-Sobic.002G206400 | 0.361662038 | 0.905030574 | 0.399613061 | Purifying | 69.62 |
| 3 | ZmAP2-3-Sobic.007G142700 | 0.098204083 | 0.257741272 | 0.381018073 | Purifying | 19.83 |
| 4 | ZmAP2-5-Sobic.001G075700 | 0.081679232 | 0.222032469 | 0.367870665 | Purifying | 17.08 |
| 5 | ZmAP2-5-Sobic.002G022600 | 0.238543772 | 0.603222116 | 0.395449314 | Purifying | 46.40 |
| 6 | ZmAP2-8-Sobic.001G036800 | 0.022791809 | 0.146295437 | 0.155793028 | Purifying | 11.25 |
| 7 | ZmAP2-8-Sobic.002G083600 | 0.251792689 | 0.8207455 | 0.306785342 | Purifying | 63.13 |
| 8 | ZmAP2-10-Sobic.006G245500 | 0.066606319 | 0.275391019 | 0.241860898 | Purifying | 21.18 |
| 9 | ZmAP2-11-Sobic.006G240700 | 0.080144738 | 0.251764439 | 0.318332239 | Purifying | 19.37 |
| 10 | ZmAP2-13-Sobic.005G021000 | 0.032241745 | 0.202509149 | 0.159211298 | Purifying | 15.58 |
| 11 | ZmAP2-14-Sobic.002G022600 | 0.090001524 | 0.219247231 | 0.410502445 | Purifying | 16.87 |
| 12 | ZmAP2-15-Sobic.003G390600 | 0.028080328 | 0.202812423 | 0.138454676 | Purifying | 15.60 |
| 13 | ZmAP2-16-Sobic.003G332300 | 0.074667353 | 0.189452783 | 0.394121172 | Purifying | 14.57 |
| 14 | ZmAP2-18-Sobic.005G021000 | 0.057231145 | 0.247329033 | 0.231396792 | Purifying | 19.03 |
| 15 | ZmAP2-20-Sobic.004G214300 | 0.02055747 | 0.135034447 | 0.152238712 | Purifying | 10.39 |
| 16 | ZmAP2-22-Sobic.004G237100 | 0.033073332 | 0.251383932 | 0.131565018 | Purifying | 19.34 |
| 17 | ZmAP2-24-Sobic.009G024600 | 0.107289885 | 0.355268145 | 0.301996919 | Purifying | 27.33 |
| 18 | ZmAP2-26-Sobic.009G124200 | 0.01280942 | 0.122874726 | 0.104247802 | Purifying | 9.45 |
| 19 | ZmAP2-29-Sobic.001G036800 | 0.26345087 | 0.822362622 | 0.320358516 | Purifying | 63.26 |
| 20 | ZmAP2-29-Sobic.002G083600 | 0.046605733 | 0.158195048 | 0.294609302 | Purifying | 12.17 |
| 21 | ZmAP2-30-Sobic.002G206400 | 0.246822343 | 0.327728049 | 0.753131579 | Purifying | 25.21 |
| 22 | ZmAP2-32-Sobic.009G024600 | 0.079079008 | 0.325499667 | 0.24294651 | Purifying | 25.04 |
| 23 | ZmAP2-34-Sobic.009G212500 | 0.046520927 | 0.300751743 | 0.154682152 | Purifying | 23.13 |
| 24 | ZmAP2-35-Sobic.010G035100 | 0.134258309 | 0.273276036 | 0.491291921 | Purifying | 21.02 |
| 25 | ZmAP2-36-Sobic.010G202700 | 0.120018433 | 0.300803621 | 0.398992647 | Purifying | 23.14 |
| 26 | ZmAP2-39-Sobic.010G215100 | 0.033035498 | 0.224695331 | 0.147023519 | Purifying | 17.28 |
| 27 | ZmAP2-40-Sobic.001G448000 | 0.060401547 | 0.183066722 | 0.329942799 | Purifying | 14.08 |
| 28 | ZmAP2-43-Sobic.007G056700 | 0.048818935 | 0.243815708 | 0.200228833 | Purifying | 18.76 |
| 29 | ZmAP2-44-Sobic.006G240700 | 0.044589652 | 0.176766833 | 0.252251238 | Purifying | 13.60 |
| 30 | ZmRAV1-Sobic.003G265200 | 0.040761129 | 0.191983962 | 0.212315281 | Purifying | 14.77 |
| 31 | ZmRAV1-Sobic.009G221400 | 0.167597921 | 0.503029093 | 0.333177391 | Purifying | 38.69 |
| 32 | ZmRAV3-Sobic.003G265200 | 0.183759001 | 0.539168795 | 0.340819059 | Purifying | 41.47 |
| 33 | ZmRAV3-Sobic.009G221400 | 0.053348845 | 0.139042287 | 0.383687916 | Purifying | 10.70 |
| 34 | ZmRAV4-Sobic.003G265200 | 0.166669725 | 0.508979366 | 0.327458708 | Purifying | 39.15 |
| 35 | ZmRAV4-Sobic.009G221400 | 0.084006844 | 0.181927137 | 0.461760929 | Purifying | 13.99 |
| 36 | ZmERF1-Sobic.001G234500 | 0.209018926 | 0.732563386 | 0.285325379 | Purifying | 56.35 |
| 37 | ZmERF1-Sobic.001G502300 | 0.051888548 | 0.182159097 | 0.284852904 | Purifying | 14.01 |
| 38 | ZmERF2-Sobic.001G486800 | 0.026702449 | 0.092926123 | 0.287351379 | Purifying | 7.15 |
| 39 | ZmERF3-Sobic.001G481600 | 0.133399621 | 0.184837954 | 0.721711199 | Purifying | 14.22 |
| 40 | ZmERF4-Sobic.001G481300 | 0.522860789 | 0.751866578 | 0.695416986 | Purifying | 57.84 |
| 41 | ZmERF4-Sobic.002G375400 | 0.541289089 | 2.104131457 | 0.257250605 | Purifying | 161.86 |
| 42 | ZmERF7-Sobic.001G473900 | 0.060526328 | 0.188017071 | 0.32191932 | Purifying | 14.46 |
| 43 | ZmERF10-Sobic.001G428700 | 0.112152788 | 0.192952593 | 0.581245301 | Purifying | 14.84 |
| 44 | ZmERF11-Sobic.001G298100 | 0.08958189 | 0.182578256 | 0.490649282 | Purifying | 14.04 |
| 45 | ZmERF13-Sobic.001G169400 | 0.342994667 | 0.863629782 | 0.397154747 | Purifying | 66.43 |
| 46 | ZmERF13-Sobic.008G145000 | 0.065140548 | 0.153549854 | 0.42423061 | Purifying | 11.81 |
| 47 | ZmERF16-Sobic.007G188701 | 0.183806468 | 0.343353458 | 0.535327266 | Purifying | 26.41 |
| 48 | ZmERF17-Sobic.002G225700 | 0.383467246 | 0.579839339 | 0.661333614 | Purifying | 44.60 |
| 49 | ZmERF17-Sobic.007G156700 | 0.105860312 | 0.185715223 | 0.570014184 | Purifying | 14.29 |
| 50 | ZmERF18-Sobic.007G162700 | 0.133389742 | 0.240276303 | 0.555151465 | Purifying | 18.48 |
| 51 | ZmERF19-Sobic.007G148500 | 0.093417063 | 0.18763932 | 0.497854409 | Purifying | 14.43 |
| 52 | ZmERF20-Sobic.007G147300 | 0.103826191 | 0.237591339 | 0.436994846 | Purifying | 18.28 |
| 53 | ZmERF21-Sobic.002G184400 | 0.300382886 | 0.642582453 | 0.467462012 | Purifying | 49.43 |
| 54 | ZmERF21-Sobic.007G124100 | 0.087458926 | 0.221582078 | 0.394702165 | Purifying | 17.04 |
| 55 | ZmERF22-Sobic.001G169400 | 0.104000347 | 0.266350561 | 0.390464156 | Purifying | 20.49 |
| 56 | ZmERF22-Sobic.008G145000 | 0.313262848 | 0.869328408 | 0.36035041 | Purifying | 66.87 |
| 57 | ZmERF23-Sobic.001G039800 | 0.081816662 | 0.168851192 | 0.484548913 | Purifying | 12.99 |
| 58 | ZmERF23-Sobic.002G081300 | 0.539698277 | 1.194091097 | 0.451974123 | Purifying | 91.85 |
| 59 | ZmERF24-Sobic.001G002500 | 0.087536422 | 0.215623369 | 0.405969087 | Purifying | 16.59 |
| 60 | ZmERF25-Sobic.006G247000 | 0.11380042 | 0.160952437 | 0.707043781 | Purifying | 12.38 |
| 61 | ZmERF26-Sobic.006G240500 | 0.045382462 | 0.21214709 | 0.213919796 | Purifying | 16.32 |
| 62 | ZmERF27-Sobic.006G210300 | 0.087472186 | 0.173789337 | 0.503323091 | Purifying | 13.37 |
| 63 | ZmERF27-Sobic.007G077300 | 0.442997497 | 0.730086859 | 0.60677369 | Purifying | 56.16 |
| 64 | ZmERF28-Sobic.004G283201 | 0.272216668 | 0.594415576 | 0.457956822 | Purifying | 45.72 |
| 65 | ZmERF28-Sobic.006G184800 | 0.049929937 | 0.166029054 | 0.300730119 | Purifying | 12.77 |
| 66 | ZmERF29-Sobic.004G283300 | 0.28595225 | 0.643257402 | 0.444537831 | Purifying | 49.48 |
| 67 | ZmERF29-Sobic.006G184700 | 0.041568107 | 0.14001935 | 0.296874017 | Purifying | 10.77 |
| 68 | ZmERF30-Sobic.004G295500 | 0.233790978 | 0.344481255 | 0.67867547 | Purifying | 26.50 |
| 69 | ZmERF30-Sobic.006G170100 | 0.09643117 | 0.174564308 | 0.55241058 | Purifying | 13.43 |
| 70 | ZmERF32-Sobic.004G296900 | 0.383673395 | 0.773487396 | 0.496030572 | Purifying | 59.50 |
| 71 | ZmERF32-Sobic.006G167900 | 0.1805169 | 0.544043448 | 0.331806036 | Purifying | 41.85 |
| 72 | ZmERF33-Sobic.006G167800 | 0.802089416 | 0.956222759 | 0.838810213 | Purifying | 73.56 |
| 73 | ZmERF34-Sobic.004G297100 | 0.161929901 | 0.464420416 | 0.348670936 | Purifying | 35.72 |
| 74 | ZmERF35-Sobic.004G227400 | 0.329929558 | 0.834021287 | 0.395588893 | Purifying | 64.16 |
| 75 | ZmERF35-Sobic.006G156100 | 0.047125075 | 0.156305297 | 0.301493783 | Purifying | 12.02 |
| 76 | ZmERF36-Sobic.004G159500 | 0.286045624 | 0.643564318 | 0.444470919 | Purifying | 49.50 |
| 77 | ZmERF36-Sobic.006G059800 | 0.091964079 | 0.204023418 | 0.450752567 | Purifying | 15.69 |
| 78 | ZmERF37-Sobic.004G158901 | 0.635749842 | 0.986126548 | 0.644693973 | Purifying | 75.86 |
| 79 | ZmERF38-Sobic.002G212000 | 0.126988987 | 0.477340733 | 0.266034256 | Purifying | 36.72 |
| 80 | ZmERF38-Sobic.010G071700 | 0.58081312 | 1.950518762 | 0.297773665 | Purifying | 150.04 |
| 81 | ZmERF40-Sobic.002G225700 | 0.1064111 | 0.185071691 | 0.574972321 | Purifying | 14.24 |
| 82 | ZmERF40-Sobic.007G156700 | 0.352464964 | 0.610971241 | 0.576892889 | Purifying | 47.00 |
| 83 | ZmERF40-Sobic.004G308900 | 0.48613838 | 0.666689028 | 0.729183111 | Purifying | 51.28 |
| 84 | ZmERF41-Sobic.002G269100 | 0.119032888 | 0.226641344 | 0.525203767 | Purifying | 17.43 |
| 85 | ZmERF41-Sobic.007G181500 | 0.323649775 | 0.815406492 | 0.39691832 | Purifying | 62.72 |
| 86 | ZmERF42-Sobic.002G350500 | 0.06231186 | 0.168573861 | 0.369641292 | Purifying | 12.97 |
| 87 | ZmERF44-Sobic.002G411000 | 0.089854399 | 0.189944966 | 0.473054912 | Purifying | 14.61 |
| 88 | ZmERF45-Sobic.003G012800 | 0.093630301 | 0.298663606 | 0.313497523 | Purifying | 22.97 |
| 89 | ZmERF46-Sobic.003G148600 | 0.089786796 | 0.186455501 | 0.48154544 | Purifying | 14.34 |
| 90 | ZmERF47-Sobic.003G442100 | 0.065942568 | 0.238378618 | 0.276629544 | Purifying | 18.34 |
| 91 | ZmERF48-Sobic.003G381400 | 0.081981614 | 0.174764201 | 0.469098437 | Purifying | 13.44 |
| 92 | ZmERF49-Sobic.003G380900 | 0.109931201 | 0.295670496 | 0.371803079 | Purifying | 22.74 |
| 93 | ZmERF49-Sobic.009G141500 | 0.273681292 | 0.499510024 | 0.547899499 | Purifying | 38.42 |
| 94 | ZmERF50-Sobic.003G369100 | 0.054706403 | 0.174047556 | 0.314318706 | Purifying | 13.39 |
| 95 | ZmERF50-Sobic.009G151600 | 0.407059595 | 0.68895786 | 0.590833807 | Purifying | 53.00 |
| 96 | ZmERF51-Sobic.003G324400 | 0.528344405 | 0.973555017 | 0.542695991 | Purifying | 74.89 |
| 97 | ZmERF51-Sobic.009G184300 | 0.376637741 | 0.980380182 | 0.384175188 | Purifying | 75.41 |
| 98 | ZmERF53-Sobic.003G297600 | 0.113245108 | 0.164650348 | 0.687791492 | Purifying | 12.67 |
| 99 | ZmERF54-Sobic.003G243500 | 0.0668812 | 0.16514462 | 0.404985643 | Purifying | 12.70 |
| 100 | ZmERF55-Sobic.005G087600 | 0.09561367 | 0.27716015 | 0.344976253 | Purifying | 21.32 |
| 101 | ZmERF56-Sobic.007G077001 | 0.151850632 | 0.2459523 | 0.617398705 | Purifying | 18.92 |
| 102 | ZmERF57-Sobic.007G162700 | 0.117646838 | 0.266828005 | 0.440908885 | Purifying | 20.53 |
| 103 | ZmERF58-Sobic.002G139300 | 0.141342109 | 0.427676518 | 0.330488355 | Purifying | 32.90 |
| 104 | ZmERF58-Sobic.007G164900 | 0.05601358 | 0.192864006 | 0.290430449 | Purifying | 14.84 |
| 105 | ZmERF59-Sobic.004G179900 | 0.048349521 | 0.202305933 | 0.238992104 | Purifying | 15.56 |
| 106 | ZmERF59-Sobic.006G091200 | 0.251828354 | 0.811417118 | 0.310356225 | Purifying | 62.42 |
| 107 | ZmERF60-Sobic.004G200700 | 0.05901808 | 0.186548036 | 0.316369346 | Purifying | 14.35 |
| 108 | ZmERF61-Sobic.004G227400 | 0.091657221 | 0.160513983 | 0.571023282 | Purifying | 12.35 |
| 109 | ZmERF61-Sobic.006G156100 | 0.336892875 | 0.730979605 | 0.460878625 | Purifying | 56.23 |
| 110 | ZmERF62-Sobic.004G295500 | 0.421184497 | 0.637752167 | 0.660420331 | Purifying | 49.06 |
| 111 | ZmERF62-Sobic.006G169900 | 0.577118973 | 1.135815373 | 0.508109845 | Purifying | 87.37 |
| 112 | ZmERF64-Sobic.004G283300 | 0.10550747 | 0.206536261 | 0.510842355 | Purifying | 15.89 |
| 113 | ZmERF64-Sobic.006G184700 | 0.276473283 | 0.595760572 | 0.464067774 | Purifying | 45.83 |
| 114 | ZmERF65-Sobic.004G331300 | 0.043077719 | 0.163716552 | 0.263123788 | Purifying | 12.59 |
| 115 | ZmERF65-Sobic.010G063900 | 0.128691395 | 0.517211142 | 0.24881791 | Purifying | 39.79 |
| 116 | ZmERF66-Sobic.010G071700 | 0.277905816 | 0.763142947 | 0.364159581 | Purifying | 58.70 |
| 117 | ZmERF66-Sobic.004G321300 | 0.05535043 | 0.160277368 | 0.345341519 | Purifying | 12.33 |
| 118 | ZmERF67-Sobic.004G319700 | 0.027331126 | 0.174401376 | 0.156713936 | Purifying | 13.42 |
| 119 | ZmERF68-Sobic.004G310600 | 0.076680506 | 0.155656958 | 0.49262498 | Purifying | 11.97 |
| 120 | ZmERF68-Sobic.010G080400 | 0.335504385 | 0.509194401 | 0.658892527 | Purifying | 39.17 |
| 121 | ZmERF69-Sobic.004G308900 | 0.142107784 | 0.183875151 | 0.772849313 | Purifying | 14.14 |
| 122 | ZmERF70-Sobic.004G233200 | 0.05166068 | 0.095863138 | 0.538900369 | Purifying | 7.37 |
| 123 | ZmERF70-Sobic.010G089700 | 0.207740737 | 0.5315017 | 0.390856203 | Purifying | 40.88 |
| 124 | ZmERF71-Sobic.005G014800 | 0.097752028 | 0.259126233 | 0.377237099 | Purifying | 19.93 |
| 125 | ZmERF72-Sobic.008G050600 | 0.437154488 | 0.820719471 | 0.532647882 | Purifying | 63.13 |
| 126 | ZmERF72-Sobic.005G051200 | 0.186506227 | 0.360201111 | 0.517783596 | Purifying | 27.71 |
| 127 | ZmERF73-Sobic.004G073100 | 0.044432125 | 0.274180334 | 0.162054384 | Purifying | 21.09 |
| 128 | ZmERF74-Sobic.010G240900 | 0.196893175 | 0.579914734 | 0.339520904 | Purifying | 44.61 |
| 129 | ZmERF75-Sobic.001G234500 | 0.12485085 | 0.317042585 | 0.393798361 | Purifying | 24.39 |
| 130 | ZmERF75-Sobic.001G502300 | 0.214363514 | 0.87886177 | 0.243910387 | Purifying | 67.60 |
| 131 | ZmERF76-Sobic.010G240900 | 0.180730393 | 0.690130674 | 0.26187851 | Purifying | 53.09 |
| 132 | ZmERF77-Sobic.004G073100 | 0.035794304 | 0.232227243 | 0.15413482 | Purifying | 17.86 |
| 133 | ZmERF78-Sobic.004G084600 | 0.020794955 | 0.129429647 | 0.160666091 | Purifying | 9.96 |
| 134 | ZmERF78-Sobic.010G185300 | 0.181885963 | 1.205958195 | 0.150822776 | Purifying | 92.77 |
| 135 | ZmERF79-Sobic.004G100500 | 0.213089938 | 0.528438273 | 0.40324471 | Purifying | 40.65 |
| 136 | ZmERF79-Sobic.010G164800 | 0.35542117 | 0.607335692 | 0.585213705 | Purifying | 46.72 |
| 137 | ZmERF82-Sobic.004G159500 | 0.087979992 | 0.210987509 | 0.416991473 | Purifying | 16.23 |
| 138 | ZmERF83-Sobic.004G179900 | 0.114595785 | 0.228397232 | 0.501738938 | Purifying | 17.57 |
| 139 | ZmERF83-Sobic.006G091200 | 0.304328224 | 0.826475997 | 0.368223911 | Purifying | 63.58 |
| 140 | ZmERF84-Sobic.004G227400 | 0.062284473 | 0.189929672 | 0.327934399 | Purifying | 14.61 |
| 141 | ZmERF84-Sobic.006G156100 | 0.34048081 | 0.688187668 | 0.494749943 | Purifying | 52.94 |
| 142 | ZmERF85-Sobic.004G297100 | 0.059953275 | 0.178695154 | 0.335505881 | Purifying | 13.75 |
| 143 | ZmERF85-Sobic.006G167800 | 0.159029602 | 0.495816783 | 0.320742676 | Purifying | 38.14 |
| 144 | ZmERF86-Sobic.004G296900 | 0.087630729 | 0.221721851 | 0.395228206 | Purifying | 17.06 |
| 145 | ZmERF87-Sobic.006G169900 | 0.364198146 | 0.874403757 | 0.416510272 | Purifying | 67.26 |
| 146 | ZmERF87-Sobic.004G295700 | 0.082157805 | 0.213800946 | 0.384272414 | Purifying | 16.45 |
| 147 | ZmERF88-Sobic.004G295500 | 0.375082315 | 0.602481593 | 0.622562281 | Purifying | 46.34 |
| 148 | ZmERF90-Sobic.004G283300 | 0.104977833 | 0.217903048 | 0.481763949 | Purifying | 16.76 |
| 149 | ZmERF90-Sobic.006G184700 | 0.258389323 | 0.647434504 | 0.39909724 | Purifying | 49.80 |
| 150 | ZmERF91-Sobic.004G283201 | 0.067743669 | 0.296043959 | 0.228829763 | Purifying | 22.77 |
| 151 | ZmERF91-Sobic.006G184800 | 0.270910791 | 0.598717711 | 0.452485013 | Purifying | 46.06 |
| 152 | ZmERF92-Sobic.004G233200 | 0.030204245 | 0.13689014 | 0.220645881 | Purifying | 10.53 |
| 153 | ZmERF92-Sobic.010G089700 | 0.22072502 | 0.536408753 | 0.411486611 | Purifying | 41.26 |
| 154 | ZmERF93-Sobic.004G308900 | 0.130602641 | 0.188586763 | 0.692533447 | Purifying | 14.51 |
| 155 | ZmERF94-Sobic.004G310600 | 0.085906156 | 0.165407077 | 0.519362035 | Purifying | 12.72 |
| 156 | ZmERF94-Sobic.010G080400 | 0.359972768 | 0.493587378 | 0.729298974 | Purifying | 37.97 |
| 158 | ZmERF96-Sobic.010G071700 | 0.289517193 | 0.881679997 | 0.328369923 | Purifying | 67.82 |
| 159 | ZmERF96-Sobic.004G321300 | 0.058197501 | 0.147356478 | 0.394943621 | Purifying | 11.34 |
| 160 | ZmERF98-Sobic.010G047500 | 0.212273822 | 0.307955564 | 0.689300167 | Purifying | 23.69 |
| 161 | ZmERF99-Sobic.010G052600 | 0.042878387 | 0.145028545 | 0.295654809 | Purifying | 11.16 |
| 162 | ZmERF100-Sobic.010G240900 | 0.036832278 | 0.161081476 | 0.228656199 | Purifying | 12.39 |
| 163 | ZmERF101-Sobic.004G084600 | 0.23752572 | 1.095649947 | 0.216789788 | Purifying | 84.28 |
| 164 | ZmERF101-Sobic.010G185300 | 0.144866281 | 0.237751946 | 0.609316908 | Purifying | 18.29 |
| 165 | ZmERF102-Sobic.004G310600 | 0.301582894 | 0.476427198 | 0.633009399 | Purifying | 36.65 |
| 166 | ZmERF102-Sobic.010G080400 | 0.109039296 | 0.218498784 | 0.499038458 | Purifying | 16.81 |
| 167 | ZmERF104-Sobic.009G103800 | 0.057043638 | 0.132331243 | 0.431067044 | Purifying | 10.18 |
| 168 | ZmERF105-Sobic.003G369100 | 0.485236652 | 0.816047751 | 0.594617964 | Purifying | 62.77 |
| 169 | ZmERF105-Sobic.009G151600 | 0.229342808 | 0.371167491 | 0.617895731 | Purifying | 28.55 |
| 170 | ZmERF106-Sobic.009G170800 | 0.081160188 | 0.162079668 | 0.500742562 | Purifying | 12.47 |
| 171 | ZmERF107-Sobic.009G184300 | 0.083762099 | 0.211803762 | 0.39547031 | Purifying | 16.29 |
| 172 | ZmERF107-Sobic.003G324500 | 0.401811425 | 0.949556795 | 0.423156811 | Purifying | 73.04 |
| 173 | ZmERF108-Sobic.003G324400 | 0.240022305 | 0.823200107 | 0.291572247 | Purifying | 63.32 |
| 174 | ZmERF108-Sobic.009G184400 | 0.13058204 | 0.253915255 | 0.514274105 | Purifying | 19.53 |
| 175 | ZmERF109-Sobic.009G233100 | 0.102684208 | 0.156126291 | 0.657699655 | Purifying | 12.01 |
| 176 | ZmERF110-Sobic.009G238500 | 0.066050176 | 0.175167714 | 0.377068207 | Purifying | 13.47 |
| 177 | ZmERF111-Sobic.002G069100 | 0.163313642 | 0.238862572 | 0.683713821 | Purifying | 18.37 |
| 178 | ZmERF113-Sobic.002G071600 | 0.236761918 | 0.605835455 | 0.390802348 | Purifying | 46.60 |
| 179 | ZmERF114-Sobic.002G081300 | 0.102221605 | 0.271144671 | 0.377000236 | Purifying | 20.86 |
| 180 | ZmERF116-Sobic.002G139400 | 0.068259904 | 0.234499436 | 0.291087709 | Purifying | 18.04 |
| 181 | ZmERF117-Sobic.002G139300 | 0.088171366 | 0.267693217 | 0.329374673 | Purifying | 20.59 |
| 182 | ZmERF118-Sobic.002G184400 | 0.04557757 | 0.174089094 | 0.261806006 | Purifying | 13.39 |
| 183 | ZmERF118-Sobic.007G124100 | 0.31697971 | 0.567279768 | 0.5587714 | Purifying | 43.64 |
| 184 | ZmERF122-Sobic.002G269100 | 0.108071123 | 0.235159649 | 0.459564912 | Purifying | 18.09 |
| 185 | ZmERF122-Sobic.007G181500 | 0.403881314 | 0.595876219 | 0.677793979 | Purifying | 45.84 |
| 186 | ZmERF124-Sobic.002G269600 | 0.062512923 | 0.179462209 | 0.348334748 | Purifying | 13.80 |
| 187 | ZmERF125-Sobic.002G350500 | 0.061234967 | 0.130181101 | 0.470382928 | Purifying | 10.01 |
| 188 | ZmERF126-Sobic.002G411000 | 0.048095426 | 0.141485151 | 0.339932674 | Purifying | 10.88 |
| 189 | ZmERF127-Sobic.002G415600 | 0.084869557 | 0.147870803 | 0.573943977 | Purifying | 11.37 |
| 190 | ZmERF128-Sobic.003G028000 | 0.122202939 | 0.230839646 | 0.529384537 | Purifying | 17.76 |
| 191 | ZmERF129-Sobic.003G012800 | 0.160138707 | 0.317512382 | 0.504354212 | Purifying | 24.42 |
| 192 | ZmERF131-Sobic.009G233100 | 0.097731706 | 0.197141082 | 0.495745003 | Purifying | 15.16 |
| 193 | ZmERF132-Sobic.009G238500 | 0.110186305 | 0.193147438 | 0.570477692 | Purifying | 14.86 |
| 194 | ZmERF137-Sobic.003G369100 | 0.364213868 | 0.826623518 | 0.44060429 | Purifying | 63.59 |
| 195 | ZmERF137-Sobic.009G151600 | 0.122514699 | 0.227605739 | 0.538275967 | Purifying | 17.51 |
| 196 | ZmERF138-Sobic.009G184300 | 0.069028113 | 0.237041396 | 0.291206999 | Purifying | 18.23 |
| 197 | ZmERF138-Sobic.003G324500 | 0.334281616 | 0.825699356 | 0.404846647 | Purifying | 63.52 |
| 198 | ZmERF139-Sobic.003G324400 | 0.207761478 | 0.830910147 | 0.250040849 | Purifying | 63.92 |
| 199 | ZmERF139-Sobic.009G184400 | 0.075757921 | 0.271995982 | 0.278525881 | Purifying | 20.92 |
| 200 | ZmERF140-Sobic.003G243500 | 0.062108109 | 0.189863654 | 0.327119527 | Purifying | 14.60 |
| 201 | ZmERF141-Sobic.009G184300 | 0.345868678 | 0.78915916 | 0.438274933 | Purifying | 60.70 |
| 202 | ZmERF141-Sobic.003G324500 | 0.118586608 | 0.188485821 | 0.629153999 | Purifying | 14.50 |
| 203 | ZmERF142-Sobic.003G324400 | 0.082054425 | 0.309062449 | 0.265494643 | Purifying | 23.77 |
| 204 | ZmERF142-Sobic.009G184400 | 0.232306192 | 0.841241087 | 0.276146987 | Purifying | 64.71 |
| 205 | ZmERF143-Sobic.004G233200 | 0.212851783 | 0.465017619 | 0.457728427 | Purifying | 35.77 |
| 206 | ZmERF143-Sobic.010G089700 | 0.090664927 | 0.146713245 | 0.617973697 | Purifying | 11.29 |
| 207 | ZmERF144-Sobic.010G071700 | 0.06235332 | 0.31385697 | 0.198667946 | Purifying | 24.14 |
| 208 | ZmERF144-Sobic.004G321300 | 0.284954709 | 1.080601162 | 0.263700169 | Purifying | 83.12 |
| 209 | ZmERF145-Sobic.004G331300 | 0.170737135 | 0.418487351 | 0.407986369 | Purifying | 32.19 |
| 210 | ZmERF145-Sobic.010G063900 | 0.074957665 | 0.243111994 | 0.308325654 | Purifying | 18.70 |
| 211 | ZmERF146-Sobic.010G052600 | 0.086624178 | 0.303496502 | 0.285420681 | Purifying | 23.35 |
| 212 | ZmERF147-Sobic.010G047500 | 0.146013454 | 0.358724523 | 0.407035051 | Purifying | 27.59 |
| 213 | ZmERF148-Sobic.004G100500 | 0.316466603 | 0.846393591 | 0.373900046 | Purifying | 65.11 |
| 214 | ZmERF148-Sobic.010G164800 | 0.111383596 | 0.233987939 | 0.476022811 | Purifying | 18.00 |
| 215 | ZmERF149-Sobic.010G200100 | 0.235703782 | 0.259834243 | 0.907131329 | Purifying | 19.99 |
| 216 | ZmERF150-Sobic.001G298100 | 0.064831473 | 0.206375657 | 0.314143025 | Purifying | 15.88 |
| 217 | ZmERF152-Sobic.001G428700 | 0.082600039 | 0.133972266 | 0.616545806 | Purifying | 10.31 |
| 218 | ZmERF153-Sobic.001G473900 | 0.050729448 | 0.161405429 | 0.314298275 | Purifying | 12.42 |
| 219 | ZmERF154-Sobic.001G486800 | 0.069908945 | 0.140016529 | 0.499290657 | Purifying | 10.77 |
| 220 | ZmERF156-Sobic.008G050600 | 0.188641117 | 0.300715003 | 0.62730863 | Purifying | 23.13 |
| 221 | ZmERF157-Sobic.007G077100 | 0.327245881 | 0.444079745 | 0.73690792 | Purifying | 34.16 |
| 222 | ZmERF158-Sobic.007G058300 | 0.130679168 | 0.376674495 | 0.346928633 | Purifying | 28.97 |
| 223 | ZmERF159-Sobic.004G158901 | 0.648140147 | 1.081831929 | 0.599113531 | Purifying | 83.22 |
| 224 | ZmERF160-Sobic.004G159500 | 0.267538355 | 0.604882216 | 0.442298265 | Purifying | 46.53 |
| 225 | ZmERF160-Sobic.006G059800 | 0.05305618 | 0.212180974 | 0.250051543 | Purifying | 16.32 |
| 226 | ZmERF161-Sobic.004G171300 | 0.424261486 | 1.189207474 | 0.356759855 | Purifying | 91.48 |
| 227 | ZmERF161-Sobic.006G080200 | 0.102164464 | 0.239525295 | 0.426528915 | Purifying | 18.43 |
| 228 | ZmERF162-Sobic.004G227400 | 0.332388803 | 0.725943801 | 0.45787126 | Purifying | 55.84 |
| 229 | ZmERF162-Sobic.006G156100 | 0.055872248 | 0.15094477 | 0.370150272 | Purifying | 11.61 |
| 230 | ZmERF163-Sobic.004G297100 | 0.183891957 | 0.557152565 | 0.330056736 | Purifying | 42.86 |
| 231 | ZmERF163-Sobic.006G167800 | 0.076741386 | 0.1699949 | 0.45143346 | Purifying | 13.08 |
| 232 | ZmERF164-Sobic.006G210300 | 0.068632062 | 0.201833987 | 0.340042144 | Purifying | 15.53 |
| 233 | ZmERF164-Sobic.007G077300 | 0.35603214 | 0.594178562 | 0.599200582 | Purifying | 45.71 |
| 234 | ZmERF165-Sobic.006G240500 | 0.074325855 | 0.194316456 | 0.382499026 | Purifying | 14.95 |
| 235 | ZmERF115-Sobic.002G115400 | 0.104507554 | 0.230355815 | 0.453678819 | Purifying | 17.72 |
| Average |  |  |  | 0.41803222 |  | 31.48 |

Table S7 **Primers for qPCR**

| Gene names | Forward primer sequence (5'→3‘) | Reverse primer sequence (5'→3‘) | Production size (bp) |
| --- | --- | --- | --- |
| ZmERF6 | AAGCGGCGGAAGGCGAAC | CTCGTCCTCCTCCTCCTCCTC | 78 |
| ZmERF21 | CTCAACTTCCCCGACAAC | ATTCTTGGATGACGACGAC | 111 |
| ZmERF28 | GAATTCCCTTACTACCCGGTG | ATCAGTAGCTCCAAAGACTGAC | 163 |
| ZmERF41 | ATCATGAAGACTGCTGTGACTC | GGGTGGTTGTCAGGCTTATTAT | 92 |
| ZmERF49 | GAGAGAGTTGAGCTGTTGAGAA | CCTCATCGTTAAGCTCTCTTCT | 88 |
| ZmERF52 | GAGTTTTTGGCTGACGACTTAG | CATACAGGACCAAAACAACCAC | 85 |
| ZmERF53 | CTGGAAGCCAAGTGAACAAC | GGACTGGACAATGCTTAGAAAC | 91 |
| ZmERF56 | GAGATGGTGATGTGATTGCTG | CTGCCTTGATCGATTAGCTACT | 87 |
| ZmERF57 | ACCAGCTACTTCGTTGCTAG | TGTGTAAGTAACTCACGAACCA | 96 |
| ZmERF68 | AACCACTGTGTTGACCTACTC | ATGTCGATGGTTAGGAGTGTAC | 86 |
| ZmERF73 | TGGTTCTTCTCTTCTCTCGTTG | GCCAGGATTCAGGCAAATAGTA | 100 |
| ZmERF74 | AAGTGCTATCGTGCTATTTTGC | GAGTAATTACCGAGTCTTGGCA | 98 |
| ZmERF77 | GCTGCTTAGAAAGAGGAAAAGG | CATCGTAGTGGATGGAGAGTTC | 84 |
| ZmERF84 | GAACACGAACCGAATTCTCAG | GTTGTCCATGTCGTAGTCCC | 171 |
| ZmERF91 | GCAAATAACTTCCCTGTACAGC | TCTTCTGCTTCTCCTGAATACC | 147 |
| ZmERF100 | GAGAAACCGAAGCACAAAAGAA | CTAGGGTTTGGCCACTAGTTTA | 103 |
| ZmERF103 | AGCAGCTAAGATAATTGCAAGC | AGGTAGCCTAACAGATTATCGC | 184 |
| ZmERF108 | CACCCGTATTACTTCTTCGGG | CAGTCATTCAGCAAAACTCCG | 243 |
| ZmERF122 | GACATGGACTTGGACTTGTACT | TGCTATAGCTTGCTAGTAGCTC | 150 |
| ZmERF123 | CCACAACATCCACTCTACTACT | CTTCTTGGTCGGACACATCTTT | 81 |
| ZmERF135 | AGTGGGTTGCTGAAATAAGAGA | GAGTGATTTGATGTCGTTGTCG | 265 |
| ZmERF137 | CATGAGCAGCTCCTACTCTTTC | TTGACGGTGGATACTCATACTG | 194 |
| ZmERF139 | CACCCAACACATACAAACACAC | CATGGTTTTCTCTGACGTTCTC | 114 |
| ZmERF142 | CCCACATCAGTACTTCCTCTAC | CCAGTTCAGATCCAGTTCGAAG | 202 |
| ZmERF153 | ACTGATTTCATAATGCGATGGC | CTAGACATGATCAAACCCGAGA | 155 |
| ZmERF157 | GAATGCCAAGCTAGCAAGAGAG | ATATGGAAAGAGCTGCCTGTAG | 87 |
| ZmERF164 | GAAGAGGAGCAGAGCGACAC | CGGCGGCATGTTGAGGTC | 90 |
